# One-year cardiovascular outcomes after coronavirus disease 2019: The cardiovascular COVID-19 registry

**DOI:** 10.1371/journal.pone.0279333

**Published:** 2022-12-30

**Authors:** Luis Ortega-Paz, Victor Arévalos, Diego Fernández-Rodríguez, Víctor Jiménez-Díaz, Jordi Bañeras, Gianluca Campo, Miguel Rodríguez-Santamarta, José Francisco Díaz, Claudia Scardino, Zaira Gómez-Álvarez, Alberto Pernigotti, Fernando Alfonso, Ignacio J. Amat-Santos, Antonio Silvestro, Lorenzo Rampa, José M. de la Torre Hernández, Gabriela Bastidas, Josep Gómez-Lara, Behnood Bikdeli, Hector M. García-García, Dominick J. Angiolillo, Josep Rodés-Cabau, Manel Sabaté, Salvatore Brugaletta, on behalf of the CV COVID-19 registry investigators

**Affiliations:** 1 Department of Cardiology, Clinic Cardiovascular Institute, Hospital Universitari Clinic, Barcelona, Spain; 2 Division of Cardiology, University of Florida College of Medicine, Jacksonville, Florida, United States of America; 3 Department of Cardiology, Hospital Universitari Arnau de Vilanova, Lérida, Spain; 4 Department of Cardiology, Hospital Universitario de Vigo, Vigo, Spain; 5 Department of Cardiology, Hospital Universitari Vall d’Hebron, Barcelona, Spain; 6 Department of Cardiology, Azienda Ospedaliero-Universitaria di Ferrara, Ferrara, Italy; 7 Department of Cardiology, Hospital Universitario de León, León, Spain; 8 Department of Cardiology, Hospital Juan Ramón Jiménez, Huelva, Spain; 9 Department of Cardiology, Hospital Universitario Joan XXIII, Tarragona, Spain; 10 Department of Cardiology, Hospital Clínico San Carlos, Madrid, Spain; 11 Department of Cardiology, Hospital de Tortosa Verge de la Cinta, Tarragona, Spain; 12 Department of Cardiology, Hospital Universitario La Princesa, Madrid, Spain; 13 Department of Cardiology, Hospital Clínico Universitario de Valladolid, Valladolid, Spain; 14 Department of Cardiology, Azienda Ospedaliera Bolognini Seriate, Bérgamo, Italy; 15 Department of Cardiology, Clinical Institute Saint Ambrogio, Milano, Italy; 16 Department of Cardiology, Hospital Marqués de Valdecilla, Santander, Spain; 17 Department of Cardiology, Hospital Universitario Sagrat Cor, Barcelona, Spain; 18 Department of Cardiology, Hospital de Bellvitge, Barcelona, Spain; 19 Cardiovascular Medicine Division, Brigham and Women’s Hospital, Harvard Medical School, Boston, Massachusetts, United States of America; 20 Center for Outcomes Research and Evaluation (CORE), Yale School of Medicine, New Haven, Connecticut, United States of America; 21 Cardiovascular Research Foundation (CRF), New York, New York, United States of America; 22 Section of Interventional Cardiology, MedStar Washington Hospital Center, Washington, DC, United States of America; Ataturk University Faculty of Medicine, TURKEY

## Abstract

**Background:**

The long-term cardiovascular (CV) outcomes of COVID-19 have not been fully explored.

**Methods:**

This was an international, multicenter, retrospective cohort study conducted between February and December 2020. Consecutive patients ≥18 years who underwent a real-time reverse transcriptase-polymerase chain reaction (RT-PCR) for SARS-CoV2 were included. Patients were classified into two cohorts depending on the nasopharyngeal swab result and clinical status: confirmed COVID-19 (positive RT-PCR) and control (without suggestive symptoms and negative RT-PCR). Data were obtained from electronic records, and clinical follow-up was performed at 1-year. The primary outcome was CV death at 1-year. Secondary outcomes included arterial thrombotic events (ATE), venous thromboembolism (VTE), and serious cardiac arrhythmias. An independent clinical event committee adjudicated events. A Cox proportional hazards model adjusted for all baseline characteristics was used for comparing outcomes between groups. A prespecified landmark analysis was performed to assess events during the post-acute phase (31–365 days).

**Results:**

A total of 4,427 patients were included: 3,578 (80.8%) in the COVID-19 and 849 (19.2%) control cohorts. At one year, there were no significant differences in the primary endpoint of CV death between the COVID-19 and control cohorts (1.4% vs. 0.8%; HR_adj_ 1.28 [0.56–2.91]; p = 0.555), but there was a higher risk of all-cause death (17.8% vs. 4.0%; HR_adj_ 2.82 [1.99–4.0]; p = 0.001). COVID-19 cohort had higher rates of ATE (2.5% vs. 0.8%, HR_adj_ 2.26 [1.02–4.99]; p = 0.044), VTE (3.7% vs. 0.4%, HR_adj_ 9.33 [2.93–29.70]; p = 0.001), and serious cardiac arrhythmias (2.5% vs. 0.6%, HR_adj_ 3.37 [1.35–8.46]; p = 0.010). During the post-acute phase, there were no significant differences in CV death (0.6% vs. 0.7%; HR_adj_ 0.67 [0.25–1.80]; p = 0.425), but there was a higher risk of deep vein thrombosis (0.6% vs. 0.0%; p = 0.028). Re-hospitalization rate was lower in the COVID-19 cohort compared to the control cohort (13.9% vs. 20.6%; p = 0.001).

**Conclusions:**

At 1-year, patients with COVID-19 experienced an increased risk of all-cause death and adverse CV events, including ATE, VTE, and serious cardiac arrhythmias, but not CV death.

**Study registration:**

URL: https://www.clinicaltrials.gov. Unique identifier: NCT04359927.

## Introduction

Coronavirus disease 2019 (COVID-19) has been associated with multi-organ involvement, including the cardiovascular (CV) system [1, 2]. A high rate of thromboembolic complications has been consistently found in hospitalized patients with COVID-19 [3–6]. In particular, critically ill patients are at higher risk of major adverse CV outcomes, including in-hospital mortality [7]. These adverse CV outcomes include venous thromboembolism (VTE), arterial thrombotic events (ATE) such as myocardial infarction (MI), stroke or systemic embolism, and other cardiac conditions such as myocarditis and arrhythmias [1, 4, 8].

Although the higher rates of adverse CV outcomes during the acute phase are well described, there are limited data beyond the acute setting [7, 9, 10]. Furthermore, the lack of a control group in these studies precludes them from determining the role of COVID-19 on CV outcomes. A study comparing the 6-month outcomes of patients with COVID-19 versus controls has suggested that adverse CV outcomes are clustered within the first month, without a significant increase thereafter [11]. However, a larger study has suggested that COVID-19 patients may exhibit an increased risk of adverse CV outcomes between 30 days and 1-year follow-up [12]. Nevertheless, this study assessed administrative data, which often are collected for non-research purposes and may not be optimal for outcome ascertainment [13]. Therefore, accuracy in adjudication of the long-term CV outcomes of patients who had COVID-19 is a critical and urgent unmet clinical need.

We aimed to determine the long-term CV outcomes, independently adjudicated, in a patient-level manually abstracted cohort of consecutive patients with COVID-19 compared to a control cohort.

## Materials and methods

### Study design and oversight

The CV COVID-19 (Long-term effects of coronavirus disease 2019 on the cardiovascular system) registry (NCT04359927) is an investigator-initiated multicenter, retrospective cohort study conducted at 17 centers in Spain and Italy. The design of the study has been reported previously (S1 File) [14]. The study was conducted in compliance with the protocol, the Declaration of Helsinki, BS EN ISO 14155 Part 1 and Part 2, and applicable local requirements and was approved by all participating centers’ institutional review boards. Initial approval was given by the “Comité de Ética de la Investigación con medicamentos del Hospital Clínic de Barcelona” (Ethics Committee for Drug Research of the Hospital Clínic of Barcelona) with the registry HCB/2020/0457, on April 16, 2020. The registry did not test clinical interventions, and individual patient care was entirely at the discretion of treating clinicians. Written informed consent was waived given the registry’s anonymous characteristics and its retrospective nature.

### Patient population

The registry included consecutive in- and outpatients with or without prior cardiovascular diseases (CVD), at least 18 years old, who underwent a nasopharyngeal swab for real-time reverse transcriptase-polymerase chain reaction (RT-PCR) for severe acute respiratory syndrome coronavirus 2 (SARS-CoV-2) between February and December 2020. All inclusion and exclusion criteria were previously reported [14]. Key exclusion criteria were terminal diseases and a life expectancy <1 year prior to index SARS-CoV-2 infection, according to medical chart review (S1 File). Patients were classified into two cohorts depending on the nasopharyngeal swab results and clinical status: those with confirmed COVID-19 (positive RT-PCR for SARS-CoV-2) and those without suggestive symptoms in conjunction with negative RT-PCR for SARS-CoV-2 (i.e., control cohort). Patients in the control cohort who developed a SARS-CoV-2 infection later during follow-up were excluded from this analysis.

### Data capture and managing

An anonymized and predefined electronic Case Report Form (eCRF) developed by the investigators was filled by each participating center. Study data elements were collected and managed using Research Electronic Data Capture (REDCap) electronic data capture tools by the investigators at Hospital Clínic of Barcelona (redcap.clinic.cat). The data elements were oriented to the cardiovascular risk factors, conditions, medications, and outcomes. Specific COVID-19 variables and treatment-related data elements were also collected (S1 File). All the data were obtained from electronic records. The investigators checked the vital patient status in the national social security database if necessary. Clinical follow-up was performed at 1-year after index RT-PCR. The flowchart and study timeline are detailed in the S1 File.

### Outcomes and definitions

The primary outcome was CV death (i.e., death resulting from cardiovascular causes) at one-year follow-up, defined according to the Academic Research Consortium-2 (S1 File) [15]. The predefined secondary outcomes were all-cause death, MI [15] stroke [15] heart failure hospitalization (HFH), documented in the diagnosis of the hospitalization discharge letter; pulmonary embolism (PE), documented with a computed tomography pulmonary angiography or invasive pulmonary angiography; serious cardiac arrhythmias defined as bradycardia requiring intravenous medication or pacemaker or leading to sudden cardiac arrest, supraventricular tachycardia requiring intravenous medication or electrical cardioversion, or ventricular tachycardia requiring intravenous medication or cardioversion or leading to sudden cardiac arrest; and major bleeding defined as a type 3 of the Bleeding Academic Research Consortium or higher [16]. Exploratory endpoints assessed were individual components of secondary endpoints, red blood cell transfusion therapy, major adverse cardiovascular events (MACE, composite of CV death, MI, and stroke), ATE (composite of MI, ischemic stroke, and systemic arterial embolism), and adverse CV events (composite of CV death, VTE, ATE, HFH, or any serious arrhythmia). Charlson Comorbidity Index was used to assess the patient’s comorbidities, and the investigators computed the index through medical chart review as previously reported [17]. Functional health status (FHS) was assessed by means of the Barthel scale and collected through medical chart review. FHS was classified according to the score into independent (80–100), partially dependent (20–79), or totally dependent (<20). Participating centers collect the Barthel scale as part of their daily clinical practice.

Independent study monitors (Effice, Madrid, Spain) verified the adequacy of the follow-up and events reported, auditing a random sample of 10% of all included patients. All outcomes were adjudicated and classified by an independent event adjudication committee by reviewing source documents provided by each center (Barcicore Lab, Barcelona, Spain).

### Statistical analysis

The prespecified statistical analysis plan is reported in the S1 File. Continuous variables are presented as mean±standard deviation or median and interquartile range (IQR). Categorical variables are reported as absolute and relative frequencies. Differences in proportions were tested with the Chi-square test or Fisher’s exact test, and differences in continuous variables were tested with a Student’s t-test or U Mann-Whitney test, as appropriate. The Kaplan-Meier method was used to derive the event rates at follow-up and plot time-to-event curves. Patients not eligible for follow-up were considered at risk until the date of the last contact, at which point they were censored.

The primary outcome analysis, CV death, was the difference in time (days) from the COVID-19 swab to CV death between the COVID-19 and control cohorts assessed by means of a Cox proportional hazards regression analysis and reported as hazard ratio (HR) and 95% confidence interval (95%CI). The model was adjusted by all baseline characteristics using a backward elimination model, including clinical characteristics and baseline medications [18]. In-hospital and discharge medications were not considered for the model adjustment because the study cohort was composed of in- and outpatients. Finally, the model was adjusted by sex, age, smoking status, diabetes mellitus, previous chronic kidney disease, previous percutaneous coronary intervention, previous heart failure, dementia, cancer, and organ transplant. The intensity of anticoagulation with low-molecular-weight heparin (LMWH) was forced into the model for adjustment for major bleeding and red blood cell transfusion therapy endpoints.

A formal sample size estimation was not performed because of the lack of reported literature assessing COVID-19-associated CV death. Therefore, the investigators agreed to target the largest possible sample size within the inclusion period.

Two prespecified sensitivity analyses were performed. First, a competing risk analysis was performed by means of Gray’s test. The model was adjusted for the risk of all-cause death. Second, an assessment of the primary endpoint in the adjudicated-only population was performed. A prespecified landmark analysis was performed, analyzing two time periods, the acute phase (0–30 days) and the post-acute phase (31–365 days). HFH was only assessed during the post-acute phase.

To determine multivariable predictors of adverse cardiovascular events (composite of cardiovascular death, any venous or arterial thrombotic event, heart failure hospitalization, or any serious arrhythmia) in the COVID-19 cohort during follow-up, a Cox proportional hazards model was used together with the Wald test to compare the results between the groups (patients with adverse CV events vs. patients without adverse CV events).

All p-values were two-sided. A p-value <0.05 was considered statistically significant for the primary endpoint, and other P-values were considered hypothesis-generating. The SAS v9.4 software was used for all analyses.

## Results

### Patient characteristics

Between February 2020 and July 2021, 4,538 consecutive patients were included. Of these, 111 were excluded from the analysis because of incomplete data, possible COVID-19 false negative results, or control patients infected during the follow-up period. A total of 4,427 patients were included in the analysis: 3,578 (80.8%) in the COVID-19 cohort and 849 (19.2%) in the control cohort. In the COVID-19 cohort, 3276 (91.6%) of the patients required hospital admission, while in the control cohort, 243 (28.6%) were admitted. A 97.4% of patients were admitted due to non-CV causes: 3,214 (98.3%) in the COVID-19 cohort and 213 (87.7%) in the control cohort (**S1** Fig and S1 Table in S1 File).

Clinical baseline characteristics are shown in Table 1. Overall, there were several differences between the COVID-19 and control cohorts. Patients in the COVID-19 cohort were older, predominantly men, with a higher frequency of comorbidities, including classical cardiovascular risk factors, cardiovascular diseases (CVD), and had a lower rate of independent FHS than those in the control cohort. In contrast, patients in the control cohort were more frequently active smokers and had a previous history of active or past cancer than those in the COVID-19 cohort.

**Table 1:** Baseline characteristics and treatments.

	COVID-19	Control	P-value
	N = 3578	N = 849	
**Characteristic**			
Age (yr), mean (SD)	63.1 (17.3)	48.8 (19.1)	0.001
Sex (males)	2032 (56.8%)	354 (41.7%)	0.001
Body mass index (kg/m^2^), mean (SD)	28.4 (5.3)	26.0 (5.0)	0.001
Weight (kg), mean (SD)	78.9 (16.7)	70.7 (15.0)	0.001
Smoking status			
Active Smoker	215 (6.0%)	143 (16.8%)	0.001
Former smoker	743 (20.8%)	140 (16.5%)	
Diabetes mellitus	721 (20.2%)	63 (7.4%)	0.001
Hypertension	1617 (45.2%)	190 (22.4%)	0.001
Hypercholesterolemia	1132 (31.6%)	117 (13.8%)	0.001
Chronic kidney disease	418 (11.6%)	56 (6.6%)	0.001
Atrial fibrillation	324 (9.1%)	36 (4.2%)	0.001
Family history of premature coronary artery disease	51 (1.4%)	2 (0.2%)	0.001
Stroke or transient ischemic attack	189 (5.3%)	22 (2.6%)	0.001
Myocardial infarction	203 (5.7%)	15 (1.8%)	0.001
Percutaneous coronary intervention	219 (6.1%)	12 (1.4%)	0.001
Coronary artery bypass grafting	52 (1.5%)	6 (0.7%)	0.072
Peripheral vascular disease	183 (5.1%)	16 (1.9%)	0.001
Chronic obstructive pulmonary disease or asthma	370 (10.3%)	86 (10.1%)	0.855
History of pneumonia	148 (4.1%)	20 (2.4%)	0.015
Heart failure	164 (4.6%)	27 (3.2%)	0.070
Pulmonary hypertension	76 (2.1%)	8 (0.9%)	0.023
Venous thromboembolism	71 (2.0%)	16 (1.9%)	0.851
Major bleeding	77 (2.2%)	15 (1.8%)	0.479
Cancer	417 (11.7%)	131 (15.4%)	0.001
Organ transplant	53 (1.5%)	15 (1.8%)	0.543
Moderate or severe valvular heart disease	119 (3.3%)	15 (1.8%)	0.017
Valve repair or replacement	56 (1.6%)	8 (0.9%)	0.172
Left ventricular ejection fraction, mean (SD)	57.8 (9.6)	56.3 (9.9)	0.138
Dementia	200 (5.6%)	18 (2.1%)	0.001
Functional health status			0.001
Independent	3053 (85.4%)	806 (94.9%)	
Partially dependent	354 (9.9%)	29 (3.4%)	
Totally dependent	168 (4.7%)	14 (1.6%)	
Charlson Comorbidity index, mean (SD)	2.9 (2.6)	1.7 (2.5)	0.001
**Medical treatments**			
Aspirin	587 (16.4%)	53 (6.2%)	0.001
P2Y_12_ inhibitors	91 (2.5%)	14 (1.6%)	0.124
Angiotensin-converting-enzyme inhibitors	576 (16.1%)	80 (9.4%)	0.001
Angiotensin II receptor blockers	509 (14.2%)	60 (7.1%)	0.001
Angiotensin receptor neprilysin inhibitors	16 (0.4%)	2 (0.2%)	0.384
Statins	896 (25.0%)	101 (11.9%)	0.001
β-blockers	581 (16.2%)	61 (7.2%)	0.001
Calcium channel blockers	423 (11.8%)	44 (5.2%)	0.001
Loop diuretics	385 (10.8%)	39 (4.6%)	0.001
Mineralocorticoid receptor antagonists	90 (2.5%)	15 (1.8%)	0.197
Proton-pump inhibitors	992 (27.7%)	130 (15.3%)	0.001
Oral hypoglycemic agents	514 (14.4%)	44 (5.2%)	0.001
Insulin	197 (5.5%)	20 (2.4%)	0.001
Anticoagulant treatment	320 (8.9%)	36 (4.2%)	0.001
Vitamin K antagonists	166 (51.9%)	13 (36.1%)	0.073
Directly acting oral anticoagulants	121 (37.8%)	20 (55.6%)	0.039
Low-molecular-weight heparin	33 (10.3%)	3 (8.3%)	0.709
Nonsteroidal anti-inflammatory drugs	77 (2.1%)	10 (1.2%)	0.080
Paracetamol	548 (15.3%)	31 (3.7%)	0.001

Data are shown as n (%), unless otherwise indicated. SD = standard deviation.

### Index hospitalization characteristics

Details on index hospital admission and in-hospital treatments are shown in Table 2. Patients in the COVID-19 cohort had longer length of stay, higher rates of intensive care unit (ICU) admission, invasive mechanical ventilation, renal replacement therapy, and vasoactive drugs compared to the control cohort. Moreover, patients with COVID-19 were more frequently exposed to full-intensity regimens of LMWH but less exposed to non-antithrombotic CV medications compared to the control cohort. In-hospital COVID-19 specific medications, biomarkers, and discharge medications are shown in S1 Table in S1 File.

**Table 2:** In-hospital characteristics and treatments.

	COVID-19	Control	P-value
	N = 3578	N = 849	
**Hospital admission**	3276 (91.6%)	243 (28.6%)	0.001
Length of stay (days), median (IQR)	9.0 (5.0–17.0)	5.0 (3.0–9.0)	0.001
Intensive Care Unit	675 (20.6%)	28 (11.5%)	0.001
Length of stay (days), median (IQR)	11.0 (5.0–23.9)	4.0 (2.0–6.5)	0.001
Invasive mechanical ventilation	420 (62.2%)	10 (35.7%)	0.005
Renal replacement therapy	131 (4.0%)	2 (0.8%)	0.012
Extracorporeal membrane oxygenation	18 (2.6%)	0 (0.0%)	0.681
Left ventricular ejection fraction, mean (SD)	57.8 (9.6)	56.3 (9.8)	0.138
**In-hospital treatments**			
Vasoactive agents	340 (10.4%)	12 (4.9%)	0.006
Anticoagulant therapy	2048 (62.5%)	143 (58.8%)	0.255
Vitamin K antagonists	57 (2.8%)	4 (2.8%)	0.992
Directly acting oral anticoagulants*	63 (3.1%)	7 (4.9%)	0.232
Low-molecular-weight heparin	1919 (93.7%)	135 (94.4%)	0.737
Enoxaparin	1702 (88.9%)	129 (95.6%)	
Intensity			0.005
Standard prophylactic intensity	1240 (66.2%)	105 (79.5%)	
Intermediate intensity	242 (12.9%)	13 (9.8%)	
Full intensity	391 (20.9%)	14 (10.6%)	
Unfractionated heparin*	57 (2.8%)	4 (2.8%)	0.992
Aspirin	420 (12.8%)	34 (14.0%)	0.001
P2Y_12_ inhibitors	75 (2.3%)	17 (7.0%)	0.001
Angiotensin-converting-enzyme inhibitors	338 (10.3%)	31 (12.8%)	0.001
Angiotensin II receptor blockers	218 (6.7%)	24 (9.9%)	0.001
Statins	478 (14.6%)	50 (20.6%)	0.001
β-blockers	446 (13.6%)	31 (12.8%)	0.001
Calcium channel blockers	344 (10.5%)	37 (15.2%)	0.001
Loop diuretics	595 (18.2%)	50 (20.6%)	0.001
Mineralocorticoid receptor antagonists	94 (2.9%)	8 (3.3%)	0.001

Data are shown as n (%) unless otherwise indicated.

*Anticoagulant therapy intensity was only computed for LMWH.

IQR = Interquartile Range; SD = standard deviation.

### One-year follow-up

At one-year, clinical follow-up was obtained in 98.3% of patients in the COVID-19 cohort and in 96.7% of controls (S1 Fig in S1 File). Re-hospitalization rate was lower in the COVID-19 cohort compared to the control cohort (13.9% vs. 20.6%; p = 0.001). There was no significant difference in the vaccination rate between COVID-19 and control cohorts. In both cohorts, compared to discharge, there was a numerical increase in the use rate of renin‐angiotensin system drugs, statins, β-blockers, and oral hypoglycemic agents. In the COVID-19 cohort, there was a pronounced decrease in anticoagulation and corticosteroids use rates, with modest changes in the control cohort. Detailed vaccination status and medications at follow-up are displayed in S2 Table in S1 File.

#### Long-term outcomes

**Fig 1:**
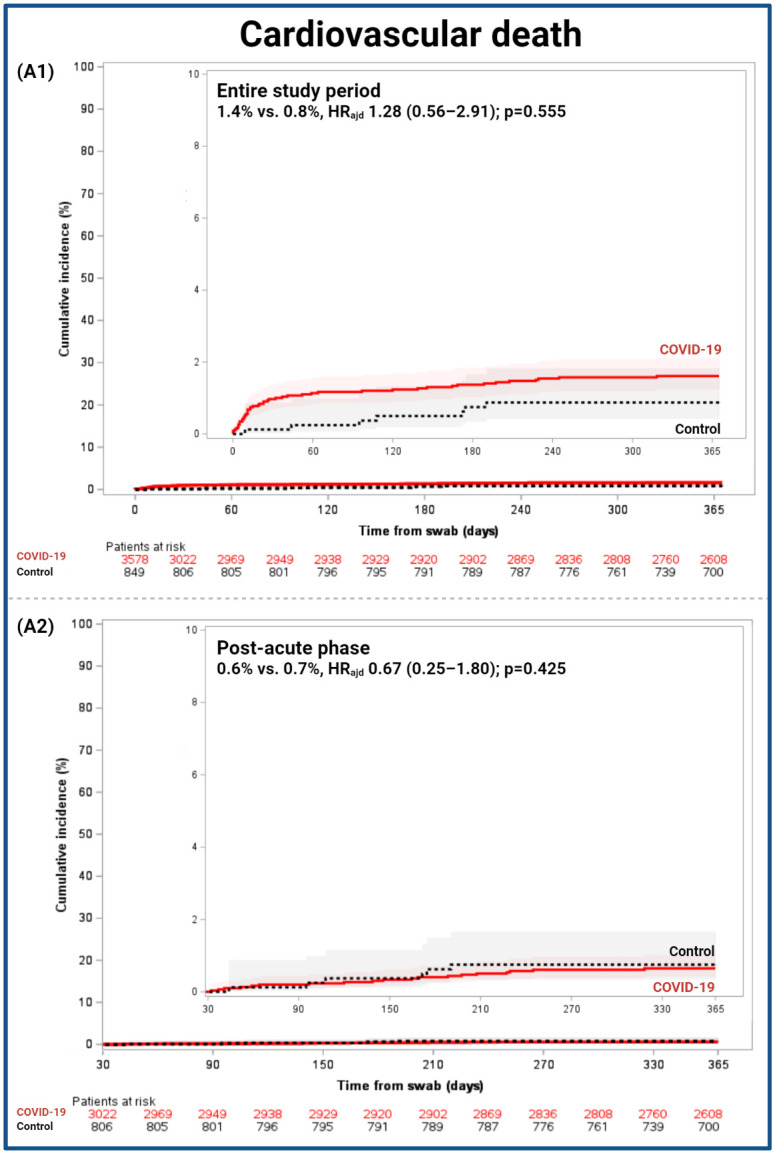
Cumulative incidence of cardiovascular death in the COVID-19 and control cohorts. Cumulative 1-year incidences are shown with Kaplan-Meier event rates with 95% CI. (A1) Entire study comprised from 0–365 days and (A2) post-acute phase from 31–365 days. Adj HR, adjusted hazard ratio; 95%CI, 95% confidence interval.

A total of 577 (84.0%) deaths were adjudicated, the remaining 16.0% were not adjudicated due to the absence of source documents. There was no difference in CV death, primary endpoint, between the COVID-19 cohort and control cohorts (1.4% vs. 0.8%; HR_adj_ 1.28 [0.56–2.91]; p = 0.555) (Fig 1A1), CV death accounted for the 8.6% of all reported deaths. The reasons for CV death are reported in S3 Table in S1 File, there were no differences between cohorts in the rates of type of CV death (p = 0.419). In the prespecified sensitivity analyses, there were no differences between the COVID-19 cohort and control in the competing risk analysis (1.4% vs. 0.8%, HR_adj_ 1.07 [0.51–2.25]; p = 0.853) and adjudicated-only population (0.7% vs. 0.8%, HR_adj_ 0.73 [0.29–1.83]; p = 0.461) (S4 Table in S1 File).

**Fig 2:**
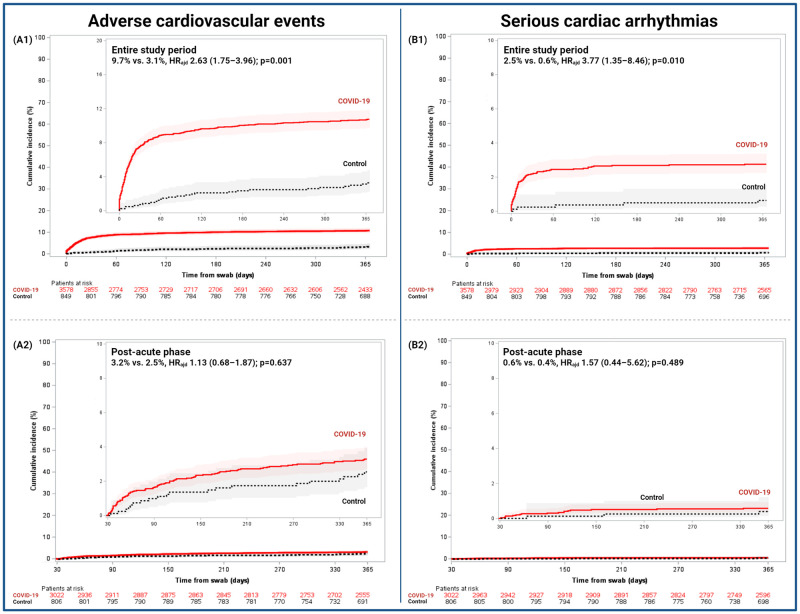
Cumulative incidence of adverse cardiovascular events and serious cardiac arrythmias in the COVID-19 and control cohorts. Cumulative 1-year incidences are shown with Kaplan-Meier event rates with 95% CI. (A1 and B1) Entire study comprised from 0–365 days and (A2 and B2) post-acute phase from 31–365 days. Adj HR, adjusted hazard ratio; 95%CI, 95% confidence interval.

Among the secondary endpoints, patients in the COVID-19 cohort had higher rate of all-cause death (17.8% vs. 4.0%, HR_adj_ 2.82 [1.99–4.00]; p = 0.001), PE (2.6% vs. 0.4%, HR_adj_ 5.96 [1.85–19.14]; p = 0.003), and serious cardiac arrhythmias (2.5% vs. 0.6%, HR_adj_ 3.37 [1.35–8.46]; p = 0.010) compared to the control cohort (Fig 2B1 and S2 Fig in S1 File). There were no differences between cohorts in rates of MI (1.6% vs. 0.8%, HR_adj_ 1.44 [0.64–3.26]; p = 0.383) or stroke (1.1% vs. 0.2%, HR_adj_ 3.27 [0.77–13.90]; p = 0.109). Similar results were found in the competing risk analysis (S4 Table in S1 File).

**Fig 3:**
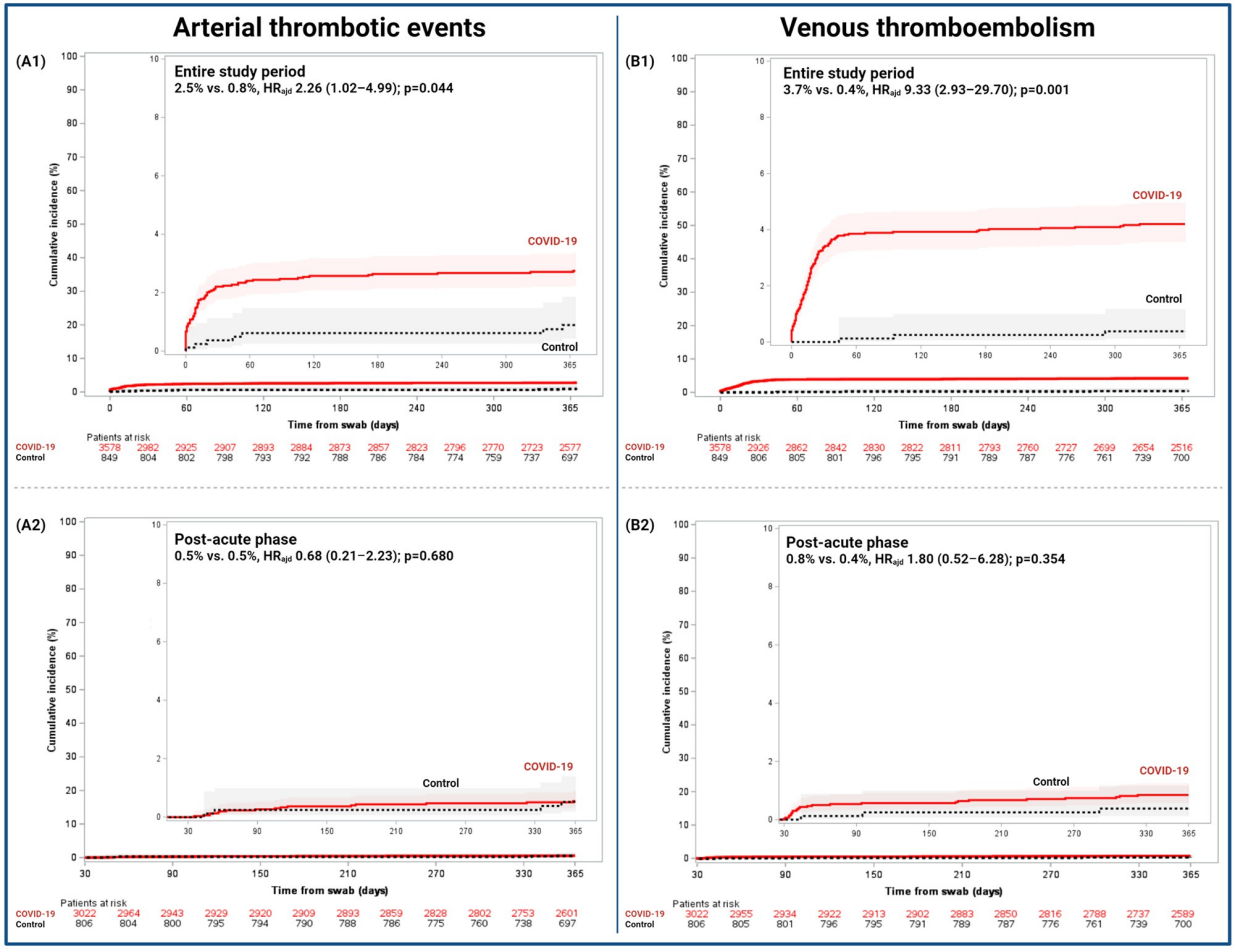
Cumulative incidence of arterial thrombotic events and venous thromboembolism in the COVID-19 and control cohorts. Cumulative 1-year incidences are shown with Kaplan-Meier event rates with 95% CI. (A1 and B1) Entire study comprised from 0–365 days and (A2 and B2) post-acute phase from 31–365 days. Adj HR, adjusted hazard ratio; 95%CI, 95% confidence interval.

Among exploratory endpoints, patients in the COVID-19 cohort had higher rate of VTE (3.7% vs. 0.4%, HR_adj_ 9.33 [2.93–29.70]; p = 0.001), deep vein thrombosis (DVT) (1.8% vs. 0.0%; p = 0.001), ischemic stroke (0.7% vs. 0.0%; p = 0.017), ATE (2.6% vs. 0.9%, HR_adj_ 2.25 [1.07–4.73]; p = 0.033), and adverse CV events (9.7% vs. 3.1%, HR_adj_ 2.63 [1.75–3.96]; p = 0.001) compared to the control cohort (Fig 3A1 and 3B1). In the COVID-19 cohort there was a numerically higher rate of MACE compared to the control (3.7% vs. 1.5%, HR_adj_ 1.74 [0.97–3.13]; p = 0.065). There were no differences between cohorts in other endpoints (Table 3 and S2 Fig in S1 File).

**Table 3:** Clinical outcomes.

	COVID-19	Control	Hazard Ratio* (95%CI)	P-value*
	N = 3578	N = 849		
**Entire study (0–365 days)**				
All-cause death	636 (17.8%)	34 (4.0%)	2.82 (1.99–4.00)	0.001
Cardiovascular death	51 (1.4%)	7 (0.8%)	1.28 (0.56–2.91)	0.555
Myocardial infarction	58 (1.6%)	7 (0.8%)	1.44 (0.64–3.26)	0.383
Stroke or transient ischemic attack	39 (1.1%)	2 (0.2%)	3.27 (0.77–13.90)	0.109
Transient ischemic attack	10 (0.3%)	1 (0.1%)	1.32 (0.16–10.81)	0.794
Ischemic stroke	24 (0.7%)	0 (0.0%)	-	0.017^¥^
Hemorrhagic stroke	5 (0.1%)	1 (0.1%)	1.02 (0.11–9.51)	0.986
Systemic arterial embolism	11 (0.3%)	0 (0.0%)	-	0.106^¥^
Arterial thrombotic event	90 (2.5%)	7 (0.8%)	2.26 (1.02–4.99)	0.044
Venous thromboembolism	134 (3.7%)	3 (0.4%)	9.33 (2.93–29.70)	0.001
Deep vein thrombosis	65 (1.8%)	0 (0.0%)	-	0.001^¥^
Pulmonary Embolism	93 (2.6%)	3 (0.4%)	5.96 (1.85–19.14)	0.003
Major bleeding or blood transfusion^£^	249 (7.0%)	16 (1.9%)	1.13 (0.58–2.17)	0.722
Major bleeding (BARC 3–5)^£^	102 (2.9%)	4 (0.5%)	0.98 (0.34–2.78)	0.967
Red blood cell transfusion^£^	227 (6.3%)	15 (1.8%)	1.16 (0.58–2.31)	0.168
Serious cardiac arrhythmias	90 (2.5%)	5 (0.6%)	3.37 (1.35–8.46)	0.010
Major adverse cardiovascular event	131 (3.7%)	13 (1.5%)	1.74 (0.97–3.13)	0.065
Adverse cardiovascular events	348 (9.7%)	26 (3.1%)	2.63 (1.75–3.96)	0.001
**Acute phase (0–30 days)**				
Hospitalization	3276 (91.6%)	243 (28.6%)	-	0.001^¥^
Length of stay (days), median (IQR)	8.9 (5.0–17.0)	4.9 (3.0–9.0)	-	0.001^±^
All-cause death	500 (14.0%)	15 (1.8%)	1.65 (0.94–2.87)	0.080
Cardiovascular death	32 (0.9%)	1 (0.1%)	1.35 (0.18–10.22)	0.770
Myocardial infarction	46 (1.3%)	3 (0.4%)	2.55 (0.77–8.45)	0.125
Stroke or transient ischemic attack	28 (0.8%)	2 (0.2%)	2.30 (0.53–9.96)	0.267
Transient ischemic attack	5 (0.1%)	1 (0.1%)	0.68 (0.07–6.34)	0.732
Ischemic stroke	21 (0.6%)	0 (0.0%)	-	0.025^¥^
Hemorrhagic stroke	2 (0.1%)	1 (0.1%)	0.35 (0.03–4.61)	0.430
Systemic arterial embolism	9 (0.3%)	0 (0.0%)	-	0.144
Arterial thrombotic event	74 (2.1%)	3 (0.4%)	4.29 (1.33–13.86)	0.015
Venous thromboembolism	109 (3.0%)	0 (0.0%)	-	0.001^¥^
Deep vein thrombosis	47 (1.3%)	0 (0.0%)	-	0.001^¥^
Pulmonary Embolism	80 (2.2%)	0 (0.0%)	-	0.001^¥^
Major bleeding or blood transfusion^£^	241 (6.7%)	16 (1.9%)	1.09 (0.56–2.11)	0.065
Major bleeding (BARC 3–5)^£^	79 (2.2%)	3 (0.4%)	0.97 (0.29–3.21)	0.954
Red blood cell transfusion^£^	227 (6.3%)	15 (1.8%)	1.16 (0.58–2.31)	0.168
Serious cardiac arrhythmias	73 (2.0%)	2 (0.2%)	6.03 (1.46–25.00)	0.013
Major adverse cardiovascular event	96 (2.7%)	4 (0.5%)	3.85 (1.39–10.63)	0.009
Adverse cardiovascular events	251 (7.0%)	6 (0.7%)	7.61 (3.36–17.24)	0.001
**Post-acute phase (31–365 days)**				
Rehospitalization	496 (13.9%)	175 (20.6%)	-	0.001^¥^
COVID-19 related	58 (11.7%)	-	-	-
Intensive care unit	34 (6.9%)	13 (7.4%)	-	0.798^¥^
COVID-19 related	6 (17.6%)	-	-	-
Invasive mechanical ventilation	10 (29.4%)	6 (46.2%)	-	0.279^¥^
COVID-19 related	2 (20.0%)	-	-	-
All-cause death	145 (4.8%)	27 (3.3%)	1.03 (0.67–1.57)	0.910
Cardiovascular death	19 (0.6%)	6 (0.7%)	0.67 (0.25–1.80)	0.425
Myocardial infarction	12 (0.4%)	4 (0.5%)	0.57 (0.17–1.96)	0.374
Stroke or transient ischemic attack	11 (0.4%)	0 (0.0%)	-	0.086^¥^
Transient ischemic attack	5 (0.2%)	0 (0.0%)	-	0.248^¥^
Ischemic stroke	3 (0.1%)	0 (0.0%)	-	1.000^¥^
Hemorrhagic stroke	3 (0.1%)	0 (0.0%)	-	1.000^¥^
Systemic arterial embolism	2 (0.1%)	0 (0.0%)	-	1.000^¥^
Arterial thrombotic event	16 (0.5%)	4 (0.5%)	0.68 (0.21–2.23)	0.680
Venous thromboembolism	25 (0.8%)	3 (0.4%)	1.80 (0.52–6.28)	0.354
Deep vein thrombosis	18 (0.6%)	0 (0.0%)	-	0.028^¥^
Pulmonary Embolism	13 (0.4%)	3 (0.4%)	0.91 (0.24–3.48)	0.886
Heart failure hospitalization	43 (1.2%)	14 (1.6%)	0.71 (0.36–1.40)	0.316
Major bleeding or blood transfusion	8 (0.3%)	0 (0.0%)	-	0.144^¥^
Major bleeding (BARC 3–5)^£^	23 (0.8%)	1 (0.1%)	1.03 (0.12–8.75)	0.977
Red blood cell transfusion	0 (0.0%)	0 (0.0%)	-	-
Serious cardiac arrhythmias	17 (0.6%)	3 (0.4%)	1.57 (0.44–5.62)	0.489
Major adverse cardiovascular event	35 (1.2%)	9 (1.1%)	0.80 (0.37–1.75)	0.581
Adverse cardiovascular events	97 (3.2%)	20 (2.5%)	1.13 (0.68–1.87)	0.637

Data are shown as n (%) unless otherwise indicated.

^¥^Fisher’s exact test.

*Cox proportional hazards regression analysis adjusted by sex, age, smoking status, diabetes, previous chronic kidney disease, previous PCI, previous heart failure, dementia, cancer, and organ transplant.

^£^Cox proportional hazards regression analysis adjusted by sex, age, smoking status, diabetes, previous chronic kidney disease, previous PCI, previous heart failure, dementia, cancer, organ transplant, and heparin dose type (prophylactic intensity vs. non-prophylactic intensity).

BARC = Bleeding Academic Research Consortium; IQR = Interquartile Range; 95%CI = 95% confidence interval.

At one year, patients in the COVID-19 cohort had a higher risk of non-CV deaths than the control (16.3% vs. 3.2%, HR_adj_ 3.22 [2.18–4.76]; p = 0.001). Among the causes of non-CV death, in the COVID-19 and control cohorts, the most frequent causes were death by infection (including sepsis) (81.0% vs. 29.6%), pulmonary (8.4% vs. 3.7%), and malignancy (4.1% vs. 51.9%) (S3 Table in S1 File).

#### Landmark analyses

Outcomes during the acute phase are reported in Table 3. There were no differences between the COVID-19 cohort and control in terms of CV death (0.9% vs. 0.1%, HR_adj_ 1.35 [0.18–10.22]; p = 0.770), neither in the two prespecified sensitivity analyses (S4 Table in S1 File). There was no significant difference in all-cause death between the COVID-19 and control cohorts (14.0% vs. 1.8%, HR_adj_ 1.65 [0.94–2.87]; p = 0.080). Patients in the COVID-19 cohort experienced a higher risk of ischemic stroke (0.6% vs. 0.0%; p = 0.025), ATE (2.1% vs. 0.4%, HR_adj_ 4.29 [1.33–13.86]; p = 0.015), VTE (3.0% vs. 0.0%; p = 0.001), serious cardiac arrhythmias (2.0% vs. 0.2%, HR_adj_ 6.03 [1.46–25.00]; p = 0.013), MACE (2.7% vs. 0.5%, HR_adj_ 3.85 [1.39–10.63]; p = 0.009), and adverse CV events (7.0% vs. 0.7%, HR_adj_ 7.61 [3.36–17.24]; p = 0.001) than those in control. There was no significant difference in major bleeding (BARC 3–5) between the COVID-19 and control cohorts (2.2% vs. 0.4%, HR_adj_ 0.97 [0.29–3.21]; p = 0.954) (Table 3).

In the post-acute phase, there were no differences in CV death between the COVID-19 cohort and control (0.6% vs. 0.7%, HR_adj_ 0.67 [0.25–1.80]; p = 0.425), neither in the two prespecified sensitivity analyses (Fig 1A2 and S4 Table in S1 File). There was no significant difference in all-cause death between the COVID-19 and control cohorts (4.8% vs. 3.3%, HR_adj_ 1.03 [0.67–1.57]; p = 0.910). Patients in the COVID-19 cohort had a higher risk of DVT (0.6% vs. 0.0%, p = 0.028) than controls, without difference in HFH (1.2% vs. 1.6%, HR_adj_ 0.71 [0.36–1.40]; p = 0.316). There was no significant difference in major bleeding (BARC 3–5) between the COVID-19 and control cohorts (0.8% vs. 0.1%, HR_adj_ 1.03 [0.12–8.75]; p = 0.977) (S3 Fig in S1 File).

#### Predictors of adverse CV outcomes during the post-acute phase in COVID-19 cohort

**Fig 4:**
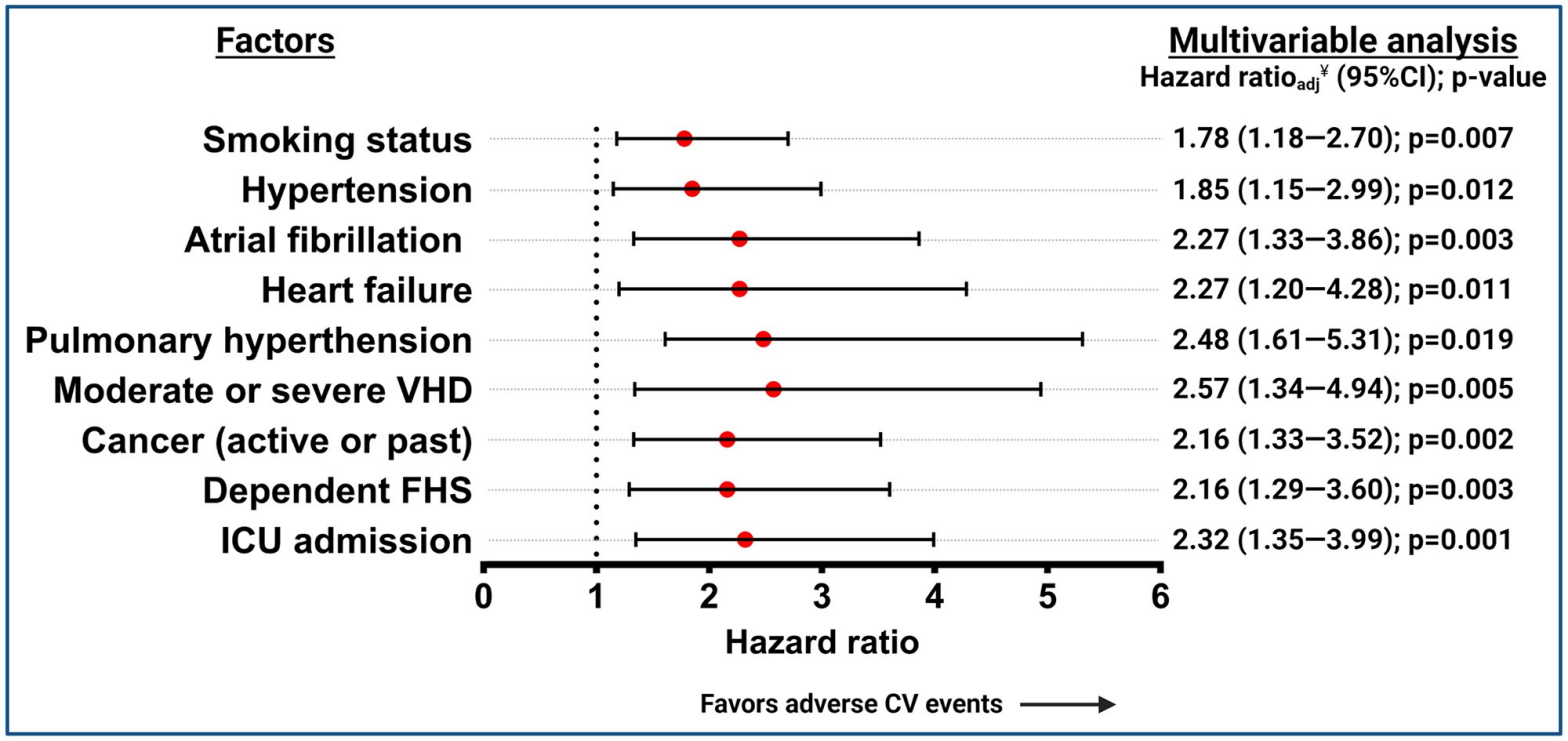
Multivariable predictors of adverse cardiovascular events during the post-acute phase in patients with COVID-19. Adverse cardiovascular events are defined as the composite of cardiovascular death, any venous or arterial thrombotic event, heart failure hospitalization, or any serious arrhythmia. Post-acute phase comprised from 31–365 days of follow-up. ^¥^Cox proportional hazards regression analysis adjusted by the significant variables in univariate analysis using a backward selection model. 95%CI, 95% confidence interval; VHD, valvular heart disease; drugs; ICU: intensive care unit; FHS, functional health status.

In the COVID-19 cohort, during the post-acute phase, the rate of adverse CV outcomes was 3.2% (97/3022). The multivariable predictors of adverse CV events were a prior history of valvular heart disease (VHD) (HR 2.57 [1.34–4.94]: p = 0.005), pulmonary hypertension (HR 2.48 [1.61–4.94], p = 0.019), atrial fibrillation (AF) (HR 2.27 [1.33–3.86]; p = 0.003), heart failure (HF) (HR 2.27 [1.20–4.28]; p = 0.011), dependent FHS (HR 2.16 [1.29–3.60]; p = 0.003), active or previous cancer (HR 2.16 [1.33–3.52]; p = 0.002), hypertension (HR 1.85 [1.15–2.99]; p = 0.012), and active or former smoker (HR 1.78 [1.18–2.70]; p = 0.007). As a factor related to hospitalization, ICU admission (HR 2.32 [1.35–3.99]; p = 0.001) was found to be a multivariable predictor of adverse CV events during the post-acute phase (Fig 4 and S5 Table in S1 File).

## Discussion

The CV COVID-19 registry, a multicenter and international study, was specifically designed to assess the long-term effects of COVID-19 on the CV system, in particular, predefined clinically relevant CV outcomes. We found that, compared with control cohort, patients with COVID-19: (1) did not experience an increased risk of CV death at one-year; (2) had an increased risk of all-cause death, ATE, VTE, and serious cardiac arrhythmias, as shown in unadjusted and multivariable-adjusted analyses; (3) a landmark analysis showed that most of the adverse CV events were clustered during the acute phase (i.e., 0–30 days), except for DVT which were increased during the post-acute phase (i.e., 31–365 days); and (4) a prior history of CV risk factors and CVD, cancer, dependent FHS, and ICU admission were among the multivariable predictors of adverse events during the post-acute phase. To the best of our knowledge, our data represent the largest patient-level manually abstracted cohort of consecutive COVID-19 patients and controls with the longest available follow-up. Moreover, our data underwent external data verification, prespecified statistical analysis, and independent event adjudication making it also of highest-quality reported in the literature.

At one-year, we did not find a higher risk of adjusted CV death in patients with COVID-19 compared to a control cohort. Notably, CV death represents a small proportion (~8%) of all deaths in the COVID-19 cohort at 1-year follow-up. However, there was a substantial impact on the CV system represented by a significantly higher risk of ATE, VTE, and serious cardiac arrhythmias. Noteworthy, the COVID-19 cohort exhibited a significantly higher burden of adverse CV events than controls. At one year, 9.7% of patients with COVID-19 had at least one serious CV event. Previous studies have reported a higher rate of VTE, acute MI, and ischemic stroke, but none reported outcomes beyond 6 months, analyzed CV death, or performed a prespecified and independent adjudication of adverse events [19–21]. Our study expands upon these findings with up to one-year follow-up. The COVID-19 cohort showed a 9-fold higher risk of VTE (both DVT and PE) and a significantly higher risk of ischemic stroke than in the control cohort. We did not find a significant difference in the risk of MI between cohorts but because of the low rate of CV events, a type II error cannot be ruled out. Moreover, when arterial events were analyzed together as ATE, there was a significantly higher rate of ATE in the COVID-19 cohort than controls, suggesting that COVID-19 could be associated with arterial thrombotic events. Ultimately, even though these adverse CV events did not translate into increased CV death, a 1.4% CV death rate at one year is a higher than expected rate in the general population but lower than previous studies focused on hospitalized patients without a control cohort [22–24].

Bleeding outcomes have been critical in patients with COVID-19 owing to the broad use of antithrombotic therapies to prevent or treat thrombotic complications [3]. A previous population study suggested that during the first 60-day of follow-up, patients who had COVID-19 had an increased bleeding risk even after adjustment of confounders [19]. We found an unadjusted higher rate of major bleeding and red blood cell transfusion in the COVID-19 cohort compared to controls. However, when adjusting for the intensity of anticoagulation with LMWH, there were no differences, suggesting that these events may be related to the treatment with intermediate- or full-intensity anticoagulation rather than to COVID-19 itself [3, 5].

In the prespecified landmark analysis, during the acute phase (0–30 days), patients with COVID-19 did not have a higher risk of CV death but had a significantly higher risk of ATE, VTE, serious cardiac arrhythmias, MACE, and any adverse CV event compared to the control. However, during the post-acute phase (31–365 days), we found only a higher risk of DVT in the COVID-19 cohort compared to control without differences in any other outcome. A study analyzing the electronic health records of 150,000 patients (85.6% outpatients) with COVID-19 from the Veteran Affairs Hospitals assessed the CV outcomes between 30 days and 1-year [12]. There was an increased risk of incident CVD, including cerebrovascular disorders, dysrhythmias, inflammatory and ischemic heart disease, HF, and VTE. Conversely, our data suggest an increased risk of ATE, VTE, and serious cardiac arrhythmias clustered during the acute phase, but if patients survive the acute phase and the thromboinflammatory states improve, the risk of adverse CV events decreases [11, 19–21]. Nevertheless, a residual VTE risk may be exhibited during the post-acute phase and mainly clustered between 30–90 days from the infection. A potential pathophysiological explanation for the increased post-acute risk could be a persistent viral replication, inflammation, prothrombotic state, and limited mobility reported in patients with post-acute COVID-19 syndrome [2, 25]. However, the underlying pathophysiological mechanisms of these conditions are not well understood and were described in high-risk patients and may not be generalizable to most patients who survived COVID-19.

In the COVID-19 cohort, we identified nine multivariable predictors of adverse CV outcomes during the post-acute phase. Of these, four are prior CVDs (VHD, pulmonary hypertension, AF, and HF), and two are CV risk factors (hypertension and smoker status), denoting that the main drivers of post-acute CV outcomes could be the prior CV history. Two variables were related to non-CV previous history (FHS and active or previous cancer), and one was related to the COVID-19 severity (ICU admission). Data from the Veteran Affairs Hospitals study also suggested that the severity of the disease (hospital admission) can be related to an increased risk of adverse CV outcomes [12]. However, up to date, there are limited data about the risk factors for long-term adverse CV outcomes [12, 26]. Ultimately, computing these factors into risk prediction tools requires further research to determine if they can adequately identify patients at higher risk who need closer follow-up.

In the CV-COVID-19 registry, most patients (~87%) were vaccinated with the BNT162b2 (BioNTech-Pfizer) and mRNA-1273 (Moderna) vaccines, which have not been related to vaccine-induced immune thrombotic thrombocytopenia (VITT). Only 44 (~2%) patients in the registry were vaccinated with ChAdOx1-S (Oxford–AstraZeneca), which has been related to VITT [27]. Considering that the average risk of these events is approximately 1 in 263,000 recipients, we believe that the occurrence of vaccine-related events (i.e., thrombotic events) in this cohort was unlikely [27].

At one-year follow-up, there was a lower readmission rate in the COVID-19 cohort compared to the control. Previous reports analyzing short-term readmission rates also found that COVID-19 patients had a lower readmission rate at 60 days compared to matched patients with pneumonia or heart failure [28]. However, dedicated analyses should be undertaken to determine the underlying reasons, timing, and outcomes of these readmissions and the potential differences between patients with and without COVID-19.

### Limitations

This study has some limitations that should be acknowledged. First, the data was not prospectively collected, increasing the risk of biases. However, our registry prespecified several approaches to minimize biases. These approaches included a dedicated and prespecified eCRF, consecutive patient inclusion, external source documents verification, independent event adjudication, and prespecified statistical plan analysis. Second, there were several baseline characteristics differences between cohorts. Nevertheless, all the reported outcomes were adjusted according to baseline characteristics. Third, due to the lack of source documents, only 84% of deaths were able to be independently adjudicated. However, all participating centers are highly experienced in CV research and adverse outcomes reporting. Furthermore, before beginning patient enrollment, centers were trained for data collection, study definitions, and outcomes reporting, including CV death. Ultimately, a worst-case scenario analysis was not performed (i.e., imputing all undetermined deaths as cardiovascular deaths) because of the extensive imbalance in the unadjudicated death rates between the COVID-19 and control cohorts. We considered this approach reasonable as the reasons leading to the imbalance were more likely related to the healthcare crisis (i.e., deaths at home or nursing auspices) during the study execution rather than COVID-19 itself. Notably, the primary outcome result was consistent in the two prespecified sensitivity analyses (competing risk and adjudicated-only analyses), denoting the robustness of the findings.

## Conclusions

At 1-year, patients with COVID-19 did not have a higher risk of CV death than controls. Nevertheless, they experienced an increased risk of all-cause death and adverse CV events, including ATE, VTE, and serious cardiac arrhythmias. Adverse events were clustered within the acute phase (0–30 days). However, during the post-acute phase (31–365 days), there was an increased risk of DVT. A follow-up extension is needed to assess the relationship between with post-acute COVID-19 syndrome, very-late adverse outcomes (i.e., >1-year), and identify potential underlying pathophysiological mechanisms.

## Supporting information

S1 File (PDF)

## Abbreviations

95%CI: 95% confidence interval
AF: atrial fibrillation
ATE: arterial thrombotic event
COVID-19: Coronavirus disease 2019
CV: cardiovascular
CVD: cardiovascular disease
DVT: deep vein thrombosis
eCRF: electronic Case Report Form
FHS: Functional health status
HF: heart failure
HFH: heart failure hospitalization
HR: hazard ratio
ICU: intensive care unit
IQR: interquartile range
LMWH: low-molecular-weight heparin
MACE: major adverse cardiovascular event
MI: myocardial infarction
PE: pulmonary embolism
RT-PCR: real-time reverse transcriptase-polymerase chain reaction
SARS-CoV-2: severe acute respiratory syndrome coronavirus 2
VTE: venous thromboembolism
