# Supplementary material for: One-year cardiovascular outcomes after coronavirus disease 2019: The cardiovascular COVID-19 registry
Source: PLoS One. 2022 Dec 30;17(12):e0279333. doi: 10.1371/journal.pone.0279333 (PMC9803130; doi:10.1371/journal.pone.0279333)
Supplement: S1 File — (PDF) [file pone.0279333.s001.pdf]

**Supplementary Material**

# One-year cardiovascular outcomes after coronavirus disease 2019: The Cardiovascular COVID-19 registry

Ortega-Paz et al.

TABLE OF CONTENTS

**CV COVID-19 registry organization** .......... 2

**Funding** .......... 2

**Manuscript Responsibility** .......... 3

**STROBE statement checklist** .......... 4

**CV COVID-19 registry inclusion and exclusion criteria** .......... 6

**CV COVID-19 registry design** .......... 7

**CV COVID-19 registry outcome definitions.** .......... 8

- Death .......... 8
- Cardiovascular death .......... 8
- Myocardial Infarction .......... 9
- Cerebrovascular accident (Stroke or TIA) .......... 10
- Heart failure hospitalization .......... 11
- Pulmonary embolism .......... 11
- Serious cardiac arrhythmias .......... 11
- Major bleeding .......... 12
- Charlson comorbidity index and Functional health status .......... 13

**Table S1. In-hospital characteristics, treatments, biomarkers, and discharge treatments** .......... 14

**Table S2. One-year follow-up, vaccination status, and treatments.** .......... 16

**Table S3. Types of cardiovascular death according to the Academic Research Consortium-2.** .......... 17

**Table S4. Pre-specified sensibility analyses** .......... 19

**Table S5. Multivariable predictors of adverse cardiovascular events during the post-acute phase in patients with COVID-19.** .......... 21

**Figure S1. CV- COVID 19 registry flow chart** .......... 22

**Figure S2. Cumulative incidence of cardiovascular outcomes in the COVID-19 and control cohorts during the entire study period** .......... 23

**Figure S3. Cumulative incidence of cardiovascular outcomes in the COVID-19 and control cohorts during the entire post-acute phase** .......... 26

**References** .......... 29

**Annex I. Electronic case report form** ..........

**Annex II. Statistical analysis plan** ..........

**CV COVID-19 registry organization.**

**Study Sponsor:** August Pi i Sunyer Biomedical Research Institute (IDIBAPS), Barcelona, *Spain*

**Data Monitoring:** Effice, Madrid, *Spain*

**Data Management**: Effice, Madrid, Spain, Enrique Conde, PhD. MB

**Statistical Committee:** José Montes (trial statistician), *Spain*

**Centralized Laboratories:** Barcicore Lab, *Barcelona, Spain*.

**Participating countries:** Spain and Italy.

**Steering Committee:** Luis Ortega-Paz, MD, PhD, (Principal Investigator [PI]), Salvatore Brugaletta (Study director), Manel Sabaté (Study Chair) Hospital Clínic of Barcelona; José María de la Torre, Hospital Marqués de Vadecilla, Santander, Spain.

**Clinical adjudication committee:** Josep Goméz-Lara, MD, PhD (Chair); Lara Fuentes, MD.

**Investigators and Clinical Sites:** Hospital Clínic, Barcelona, Spain: Luis Ortega-Paz (PI), Victor Arévalos, Salvatore Brugaletta, Manel Sabaté, Josep Rodés-Cabau, Juan José Rodríguez-Arias, Margarita Calvo, Leticia Castrillo, Anthony Salazar, Marta Sabaté Tormos, and Francesco Spione. Hospital Universitari Arnau de Vilanova, Lleida, Spain: Diego Fernandez-Rodríguez (PI), Pablo Pastor Pueyo. Complejo Hospitalario Universitario de Vigo, Vigo, Spain: Víctor Alfonso Jiménez-Díaz, (PI), Antonio Espino, María Pena, Ubaldo Hernández, Halley Moya, Carlos Enrique Ochoa, and Pablo Juan-Salvadores. Hospital Vall' de Hebron, Barcelona, Spain: Jordi Bañeras Rius (PI), Ignacio Ferreira González, Pablo Jordán, Montse Bach, and Eduard Rodenas. Azienda Ospedaliero-Universitaria di Ferrara, Ferrara, Italy: Gianluca Campo (PI), Ottavio Zuchetti. Hospital Universitario de León, León, Spain: Miguel Rodríguez-Santamarta (PI), Julio Echarte Morales, Samuel del Castillo García, and Carlos Minguito Carazo. Hospital Juan Ramón Jiménez, Huelva, Spain: José Francisco Díaz Fernández (Co-PI), Antonio Gómez-Menchero (Co-PI), and Josefa García. Hospital Universitari Joan XXIII, Tarragona, Spain: Claudia Scardino (PI). Hospital Clínico San Carlos, Madrid, Spain: Nieves Gonzalo (PI), Zaira Gómez and Teresa Romero. Hospital de Tortosa Verge de la Cinta, Tarragona, Spain: Alberto Pernigotti (PI). Hospital Universitario La Princesa, Madrid, Spain: Fernando Alfonso (PI). Hospital Clínico Universitario de Valladolid, Valladolid, Spain: Ignacio Jesús Amat-Santos (PI), Alvaro Aparisi. Ospedale Bolognini di Seriate, Bérgamo, Italy: Antonio Silvestro (PI). Istituto Clinico Sant'Ambrogio, Milano, Italy: Alfonso Ielasi (PI), Lorenzo Rampa. Hospital Marqués de Valdecilla, Santander, Spain: José María de la Torre (PI), Manuel Lozano and Miguel Molina. Hospital Universitari Sagrat Cor, Barcelona, Spain: Gabriela Bastidas (PI). Hospital de Bellvitge, Barcelona, Spain: Loreto Oyarzabal (PI).

**Funding**
The study sponsor August Pi i Sunyer Biomedical Research Institute, a non-profit organization, received grant support for the conduct of the study from the Carlos III health Institute (Madrid, Spain) and co-funded by the European Union (grant number COV20/00040) The funder and sponsor were not involved with the study processes, including site selection and management, and data collection and analysis.

**Manuscript Responsibility**

Luis Ortega-Paz, MD, PhD and Salvatore Brugaletta, MD, PhD wrote the first draft of the manuscript, which was critically reviewed and checked for consistency by the members of the statistical committee. All remaining authors critically reviewed the manuscript.
Dr. Ortega-Paz submitted the manuscript for publication on behalf of the authors.

## STROBE statement checklist

| | Item No | Recommendation | Page No |
|---|---|---|---|
| **Title and abstract** | 1 | (*a*) Indicate the study's design with a commonly used term in the title or the abstract | 1 |
| | | (*b*) Provide in the abstract an informative and balanced summary of what was done and what was found | 3 |
| **Introduction** | | | |
| Background/rationale | 2 | Explain the scientific background and rationale for the investigation being reported | 7 |
| Objectives | 3 | State specific objectives, including any prespecified hypotheses | 7-8 |
| **Methods** | | | |
| Study design | 4 | Present key elements of study design early in the paper | 8 |
| Setting | 5 | Describe the setting, locations, and relevant dates, including periods of recruitment, exposure, follow-up, and data collection | 8-9 |
| Participants | 6 | (*a*) Give the eligibility criteria, and the sources and methods of selection of participants. Describe methods of follow-up | 8 |
| | | (*b*) For matched studies, give matching criteria and number of exposed and unexposed | - |
| Variables | 7 | Clearly define all outcomes, exposures, predictors, potential confounders, and effect modifiers. Give diagnostic criteria, if applicable | |
| Data sources/ measurement | 8* | For each variable of interest, give sources of data and details of methods of assessment (measurement). Describe comparability of assessment methods if there is more than one group | 9 |
| Bias | 9 | Describe any efforts to address potential sources of bias | 10 |
| Study size | 10 | Explain how the study size was arrived at | 11 |
| Quantitative variables | 11 | Explain how quantitative variables were handled in the analyses. If applicable, describe which groupings were chosen and why | 10-11 |
| Statistical methods | 12 | (*a*) Describe all statistical methods, including those used to control for confounding | 10-11 |
| | | (*b*) Describe any methods used to examine subgroups and interactions | |
| | | (*c*) Explain how missing data were addressed | |
| | | (*d*) If applicable, explain how loss to follow-up was addressed | |
| | | (*e*) Describe any sensitivity analyses | |
| **Results** | | | |
| Participants | 13* | (a) Report numbers of individuals at each stage of study—eg numbers potentially eligible, examined for eligibility, confirmed eligible, included in the study, completing follow-up, and analysed | 12 Fig. S1 |
| | | (b) Give reasons for non-participation at each stage | |
| | | (c) Consider use of a flow diagram | |
| Descriptive data | 14* | (a) Give characteristics of study participants (eg demographic, clinical, social) and information on exposures and potential confounders | 12 Table 1 and S1 |
| | | (b) Indicate number of participants with missing data for each variable of interest | |
| | | (c) Summarise follow-up time (eg, average and total amount) | |
| Outcome data | 15* | Report numbers of outcome events or summary measures over time | |
| Main results | 16 | (*a*) Give unadjusted estimates and, if applicable, confounder-adjusted estimates and their precision (eg, 95% confidence interval). Make clear which confounders were adjusted for and why they were included | 12-14 Figure 1-3 Table 3, S4, S5. |
| | | (*b*) Report category boundaries when continuous variables were categorized | |
| | | (*c*) If relevant, consider translating estimates of relative risk into absolute risk for a meaningful time period | |
| Other analyses | 17 | Report other analyses done—eg analyses of subgroups and interactions, and sensitivity analyses | 12-13, Table S4 |
| **Discussion** | | | |
| Key results | 18 | Summarise key results with reference to study objectives | 15 |

| | | | |
|---|---|---|---|
| Limitations | 19 | Discuss limitations of the study, taking into account sources of potential bias or imprecision. Discuss both direction and magnitude of any potential bias | 18 |
| Interpretation | 20 | Give a cautious overall interpretation of results considering objectives, limitations, multiplicity of analyses, results from similar studies, and other relevant evidence | 15-17 |
| Generalisability | 21 | Discuss the generalisability (external validity) of the study results | 15-17 |
| **Other information** | | | |
| Funding | 22 | Give the source of funding and the role of the funders for the present study and, if applicable, for the original study on which the present article is based | 19 |

**Note:** An Explanation and Elaboration article discusses each checklist item and gives methodological background and published examples of transparent reporting. The STROBE checklist is best used in conjunction with this article (freely available on the Web sites of PLoS Medicine at http://www.plosmedicine.org/, Annals of Internal Medicine at http://www.annals.org/, and Epidemiology at http://www.epidem.com/). Information on the STROBE Initiative is available at http://www.strobe-statement.org.

**CV COVID-19 registry inclusion and exclusion criteria.**

| **Inclusion criteria** |
|---|
| 1. At least 18 years of age |
| 2. Patient who underwent a nasopharyngeal swab for real-time reverse transcriptase-polymerase chain reaction (RT-PCR) for SARS-CoV2 between March 2020 and December 2020. |
| **Exclusion criteria** |
| 1. Terminal diseases and a life expectancy <1 year before the diagnosis will be excluded. |

## CV COVID-19 registry design.

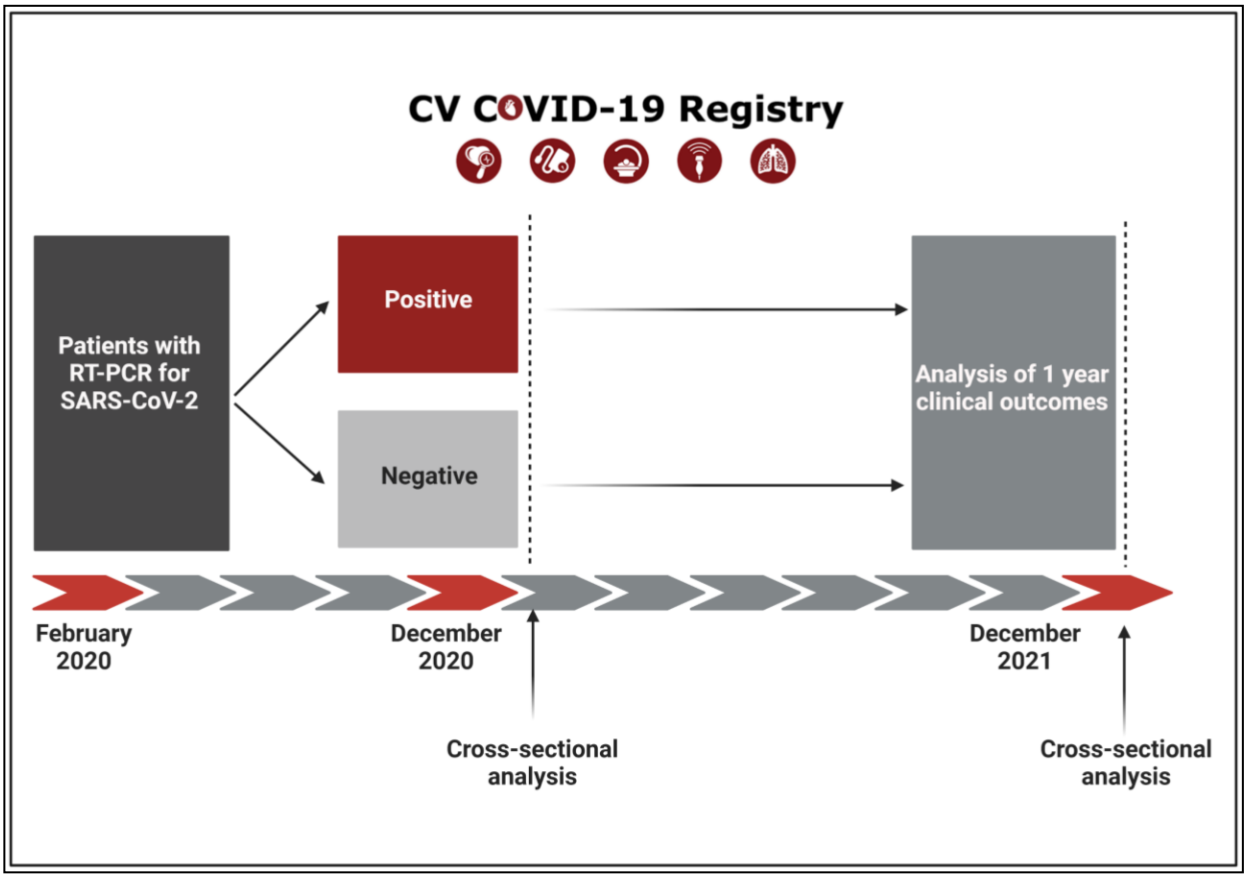


Rationale and design paper previously published.[1] CV=cardiovascular; RT-PCR= real-time reverse transcriptase-polymerase chain reaction; SARS-CoV-2=severe acute respiratory syndrome coronavirus 2

**CV COVID-19 registry outcome definitions.**
The following adverse events will be evaluated at each follow-up: death; myocardial infarction; stroke; transient ischemic attack; bleeding; coronary stent thrombosis; urgent target vessel coronary revascularization.[2]

**Death[2]**
Death from any cause (all-cause death).

**Cardiovascular death[2]**
Death due to cardiovascular causes, such as myocardial infarction, heart failure, cardiac arrhythmias, sudden death or all deaths of unknown cause.

| Type of Death | Definition |
|---|---|
| Cardiovascular death | Cardiovascular death is defined as death resulting from cardiovascular causes. The following categories may be collected: |
| | 1. Death caused by acute MI |
| | 2. Death caused by sudden cardiac, including unwitnessed, death |
| | 3. Death resulting from heart failure |
| | 4. Death caused by stroke |
| | 5. Death caused by cardiovascular procedures |
| | 6. Death resulting from cardiovascular hemorrhage |
| | 7. Death resulting from other cardiovascular cause |
| Noncardiovascular death | Noncardiovascular death is defined as any death that is not thought to be the result of a cardiovascular cause. The following categories may be collected: |
| | 1. Death resulting from malignancy |
| | 2. Death resulting from pulmonary causes |
| | 3. Death caused by infection (includes sepsis) |
| | 4. Death resulting from gastrointestinal causes |
| | 5. Death resulting from accident/trauma |
| | 6. Death caused by other noncardiovascular organ failure |
| | 7. Death resulting from other noncardiovascular cause |
| Undetermined | Undetermined cause of death is defined as a death not attributable to any other category because of the absence of any relevant source documents. Such deaths will be classified as cardiovascular for end point determination. |

MI indicates myocardial infarction.

**Myocardial Infarction**
As per the "Fourth Universal Definition of Myocardial Infarction" (Thygesen K et al. Eur Heart J 2019; 40(3): 237-269).[3]

The definition of a new myocardial infarction (Type 1 MI) is as follows:
Detection of rise and/or fall of cardiac troponin level, with at least one level above the 99th percentile upper reference limit (URL) for assay used, and at least one of the following:

- Symptoms of acute myocardial ischaemia
- New ischaemic ECG changes
- Development of pathological Q-waves
- Imaging evidence of new loss of viable myocardium, or new regional wall motion abnormality in a pattern consistent with an ischaemic aetiology
- Identification of coronary thrombus by angiography including intracoronary imaging or autopsy

The definition of a PCI-related Myocardial infarction <48hr after the index procedure (Type 4a MI) is as follows:
Elevation of cardiac troponin levels greater than 5 times the 99th percentile URL with normal baseline values. [*If elevated baseline value of cardiac troponin which is stable (<20% variation) or falling, there should be a greater than 20% rise in the post-procedure cTn level and the absolute cTn value should be greater than 5 times the 99th percentile URL.]*
In addition, there must be at least one of the following:

- New ischaemic ECG changes
- Development of new pathological Q-waves
- Imaging evidence of new loss of viable myocardium, or new regional wall motion abnormality in a pattern consistent with an ischaemic aetiology
- Angiographic findings consistent with a flow limiting procedural complication such as a coronary dissection, occlusion of a major epicardial coronary artery or a side branch occlusion/thrombus, disruption of collateral flow or distal embolization.

The definition of a CABG-related Myocardial infarction <48hr after the index procedure (Type 5 MI) is as follows:
Elevation of cardiac troponin levels greater than 10 times the 99th percentile URL with normal baseline values. [*If elevated baseline value of cardiac troponin which is stable (<20% variation) or falling, there should be a greater than 20% rise in the post-procedure cTn level and the absolute cTn value should be greater than 10 times the 99th percentile URL.]*
In addition, there must be at least one of the following:

- Development of new pathological Q-waves
- Angiographic documented new graft occlusion or new native coronary artery occlusion.
- Imaging evidence of new loss of viable myocardium, or new regional wall motion abnormality in a pattern consistent with an ischaemic aetiology

In addition to the above, other types of MI are defined as below:

| | Definition |
|---|---|
| Type 2 MI | As per the definition of Type 1 MI, however also with evidence of an imbalance between myocardial oxygen supply and demand unrelated to acute coronary athero-thombosis (eg: anaemia, non-cardiogenic shock, hypotension, sepsis, coronary vasospasm, respiratory failure, sustained tachyarrhythmia, severe hypertension +/- left ventricular hypertrophy). |
| Type 3 MI | Patients who suffer a cardiac death with symptoms suggestive of myocardial ischaemia accompanied with presumed new ischaemic ECG changes or ventricular fibrillation, but die before blood biomarkers can be obtained, or before increases in serial cardiac biomarkers can be identified, or MI is detected by autopsy examination. |
| Type 4b MI | Stent/scaffold thrombosis, as documented by angiography or at autopsy, with same criteria as described for Type 1 MI.<br>Timing in relation to index PCI procedure should be categorised as below:<br>• Acute: 0-24hr<br>• Subacute: >24hr to 30 days<br>• Late: 30 days to 1 year<br>• Very Late: >1 year |
| Type 4c MI | Evidence of focal or diffuse in-stent restenosis at angiography taken at time of presenting MI, diagnosed using the criteria as described for Type 1 MI. |

**Cerebrovascular accident (Stroke or TIA)**

Cerebrovascular accidents are composed by stroke and transient ischemic attacks (TIA).
A **stroke** is defined as a sudden, focal neurological defect resulting from a cerebrovascular cause, resulting in death or lasting greater than 24 hours, that is not due to a readily identifiable cause such as a tumor, infection or trauma. All suspected strokes will be classified as:

- Hemorrhagic stroke – a stroke with focal collections of intracranial blood
- Ischemic stroke – a stroke without focal collections of intracranial blood
- Unknown – no imaging or autopsy data are available

A **TIA** is defined as a new, transient episode of neurological dysfunction (usually 1 to 2 h), always within 24 h caused by focal brain, spinal cord, or retinal ischemia, *without* acute infarction at neuroimaging.

**Stent Thrombosis**

Stent Thrombosis should be reported as a cumulative value at the different time points and with the different separate time points. Time 0 is defined as the time point after the guiding catheter has been removed.

**Timing**:

- Acute stent thrombosis: 0-24 hours post-stent implantation
- Subacute stent thrombosis: >24 hours-30 days post stent implantation
- Late stent thrombosis: >30 days-1-year post stent implantation

**Likelihood of an accurate diagnosis:**
We recognize three categories of evidence in defining stent thrombosis: definite, probable, and possible.
Definite (is considered either by angiographic or pathologic confirmation).
Angiographic confirmation of stent thrombosis is considered to have occurred if:
1) Thrombolysis In Myocardial Infarction (TIMI) flow is:
a) TIMI flow grade 0 with occlusion originating in the stent or in the segment 5 mm proximal or distal to the stent region in the presence of a thrombus(*).
b) TIMI flow grade 1, 2, or 3 originating in the stent or in the segment 5m proximal or distal to the stent region in the presence of a thrombus(*).
AND at least one of the following criteria, has been fulfilled within a 48-hour time window:
2) new onset of ischemic symptoms at rest (typical chest pain with duration >20 minutes or requiring medical treatment)
3) new ischemic ECG changes suggestive of acute ischemia
4) typical rise and fall in cardiac biomarkers
The incidental angiographic documentation of stent occlusion in the absence of clinical signs or symptoms is not considered a confirmed stent thrombosis (silent occlusion).
(*) Intracoronary thrombus [Ellis et al., Mabin et al., Capone et al.]
*Non-occlusive thrombus*:
Intracoronary thrombus is defined as a (spheric, ovoid or irregular) non-calcified filling defect or lucency surrounded by contrast material (on three sides or within a coronary stenosis) seen in multiple projections, or persistence of contrast material within the lumen, or a visible embolization of intraluminal material downstream.
*Occlusive thrombus*:
A TIMI 0 or TIMI 1 intra-stent or proximal to a stent up to the most adjacent proximal side branch or main branch (if originating from the side branch).
Pathologic confirmation of stent thrombosis.
Evidence of recent thrombus within the stent determined at autopsy.
Probable:
Clinical definition of probable stent thrombosis is considered to have occurred after intracoronary stenting in the following cases:
1) Any unexplained death within the first 30 days.
2) Irrespective of the time after the index procedure any myocardial infarction (MI), which is related to documented acute ischemia in the territory of the implanted stent without angiographic confirmation of stent thrombosis and in the absence of any other obvious cause.
Possible:
Clinical definition of possible stent thrombosis is considered to have occurred with any unexplained death following intracoronary stenting until the end of the follow-up period.

**Heart failure hospitalization**
Must be documented in the diagnosis of the hospitalization discharge letter.

**Pulmonary embolism**
Must be documented with a computed tomography pulmonary angiography or invasive pulmonary angiography)

**Serious cardiac arrhythmias**
Bradycardia requiring intravenous medication or pacemaker or leading to sudden cardiac arrest, supraventricular tachycardia requiring intravenous medication or electrical

cardioversion, or ventricular tachycardia requiring intravenous medication or cardioversion or leading to sudden cardiac arrest.

**Major bleeding**
The primary bleeding classification used in the study will be the Bleeding Academic Research Consortium (BARC).

**BARC Classification**[4]
**Type 0:** No evidence of bleeding.
**Type 1:** Bleeding that is NOT ACTIONABLE and does not cause the patient to seek unscheduled performance of studies, hospitalization, or treatment by a healthcare professional. Examples include, but are not limited to, bruising, hematoma, nosebleeds, or hemorrhoidal bleeding for which the patient does not seek medical attention. Type I bleeding may include episodes that lead to discontinuation of medications by the patient because of bleeding without visiting a healthcare provider.
**Type 2:** Any clinically overt sign of hemorrhage (e.g., more bleeding than would be expected for a clinical circumstance; including bleeding found by imaging alone) that is actionable, but does not meet criteria for Type 3 BARC bleeding, Type 4 BARC bleeding (CABG-related), or Type 5 BARC bleeding (fatal bleeding). The bleeding must require diagnostic studies, hospitalization or treatment by a healthcare professional. In particular, the bleeding must meet at least one of the following criteria:

1) Requiring intervention: defined as a healthcare professional-guided medical treatment or percutaneous intervention to stop or treat bleeding, including temporarily or permanently discontinuing a medication or study drug. Examples include, but are not limited to, coiling, compression, use of reversal agents (e.g. vitamin K, protamine), local injections to reduce oozing, or a temporary/ permanent cessation of antiplatelet, antithrombin, or fibrinolytic therapy;
2) Leading to hospitalization or an increased level of care: defined as leading to or prolonging hospitalization or transfer to a hospital unit capable of providing a higher level of care or;
3) Prompting evaluation: defined as leading to an unscheduled visit to a healthcare professional resulting in diagnostic testing (laboratory or imaging). Examples include, but are not limited to, hematocrit testing, hem occult testing, endoscopy, colonoscopy, computed tomography scanning, or urinalysis. A visit or phone call to a healthcare professional where neither testing nor treatment is undertaken does not constitute Type 2 bleeding.

**Type 3:** Clinical, laboratory, and/or imaging evidence of bleeding with specific healthcare provider responses, as listed below:

**Type 3a**

- Any transfusion with overt bleeding
- Overt bleeding plus hemoglobin drop ≥3 to <5 g/ld.* (provided hemoglobin drop is related to bleeding)

**Type 3b**

- Overt bleeding plus hemoglobin drop ≥ 5 g/dL* (provided hemoglobin drop is related to bleeding)
- Cardiac tamponade
- Bleeding requiring surgical intervention for control (excluding dental/ nasal/skin/hemorrhoid)
- Bleeding requiring intravenous vasoactive drugs

**Type 3c**

- Intracranial hemorrhage (does not include microbleeds or hemorrhagic transformation; does include intarsia).
- Subcategories; Confirmed by autopsy or imaging or lumbar puncture (LP)
- Intra-ocular bleed compromising vision

*Hb drop should be corrected for intracurrent transfusion, where 1 unit of packed red blood cells or
1 unit of whole blood would be expected to increase Hb by 1g/dl

**Type 4:** CABG-Related Bleeding.
• Perioperative intracranial bleeding within 48 hours
• Reoperation following closure of sternotomy for the purpose of controlling bleeding
• Transfusion of ≥ 5 units of whole blood or packed red blood cells within a 48-hour period**
• Chest tube output ≥ 2L within a 24-hour period.

Note: If a CABG-related bleed is not adjudicated as at least a Type 3 severity event, it will be classified as 'not a bleeding event'.

** Only allogeneic transfusions are considered as transfusions for CABG-related bleeds. Cell saver products will not be counted.

**Type 5:** Fatal Bleeding. Fatal bleeding is bleeding that directly causes death with no other explainable cause. BARC Fatal Bleeding is categorized as either definite or probable as follows:

**Type 5a – Probable fatal bleeding**: Bleeding that is clinically suspicious as the cause of death, but the bleeding is not directly observed and there is no autopsy or confirmatory imaging.

**Type 5b** – **Definite fatal bleeding**: Bleeding that is directly observed (either by clinical specimen – blood, emesis, stool, etc.- or by imaging) or confirmed on autopsy.

The site of fatal bleeding is further categorized as intracranial, gastrointestinal, retroperitoneal, pulmonary, pericardial, gastrourinary, or other. BARC fatal bleeding is meant to capture deaths that are directly due to bleeding with no other cause. The time interval from the bleeding event to the death should be considered with respect to likely causality, but there is no specific time limit proposed. Bleeding that is contributory but not directly causal to death is not classified as fatal bleeding but may be categorized as other forms of bleeding. Bleeding that leads to cessation of antithrombotic or other therapies may be contributory, but again, would not be classified as fatal bleeding. Bleeding associated with trauma or with surgery may be fatal, depending on whether it was determined to be directly causal or not.

**Charlson comorbidity index and Functional health status**

Charlson Comorbidity Index was used to assess the patient's comorbidities, and the investigators computed the index through medical chart review as previously reported.[5] Functional health status (FHS) was assessed by means of the Barthel scale and collected through medical chart review. FHS was classified according to the score into independent (80–100), partially dependent (20–79), or totally dependent (<20).[6] Participating centers collect the Barthel scale as part of their daily clinical practice.

**S1 Table. In-hospital characteristics, treatments, biomarkers, and discharge treatments.**

| | COVID-19 N=3578 | Control N=849 | P-value |
|---|---|---|---|
| **Hospital admission cause** | 3276 (91.6%) | 243 (28.6%) | 0.001 |
| CV cause* | 62 (1.7%) | 30 (12.3%) | 0.001 |
| Ischemic heart disease | 16 (0.5%) | 8 (0.9%) | |
| Heart failure | 17 (0.5%) | 7 (0.8%) | 0.932 |
| Any arrhythmia, syncope, or sudden cardiac death | 12 (0.3%) | 3 (0.4%) | |
| Other | 17 (0.5%) | 12 (1.4%) | |
| Non-CV cause* | 3214 (98.3%) | 213 (87.7%) | 0.001 |
| COVID-19 | 3054 (93.2%) | 0 | |
| Respiratory (non-COVID-19) | 41 (1.2%) | 30 (3.5%) | |
| Cancer | 25 (0.8%) | 33 (3.9%) | |
| Endocrine and vascular | 10 (0.5%) | 15 (1.8%) | |
| Digestive | 21 (0.6%) | 35 (4.1%) | |
| Renal | 19 (0.6%) | 31 (3.7%) | 0.001 |
| Infectious disease (non-COVID-19) | 12 (0.4%) | 22 (2.6%) | |
| Trauma and/or poisoning | 10 (0.5%) | 18 (2.1%) | |
| Blood or autoimmune | 14 (0.4%) | 23 (2.7) | |
| Other | 15 (0.5%) | 6 (0.7) | |
| **Hospital admission details** | | | |
| Length of stay (days), median (IQR) | 9.0 (5.0–17.0) | 5.0 (3.0–9.0) | 0.001 |
| Intensive Care Unit | 675 (20.6%) | 28 (11.5%) | 0.001 |
| Length of stay (days), median (IQR) | 11.0 (5.0-23.9) | 4.0 (2.0-6.5) | 0.001 |
| Invasive mechanical ventilation | 420 (62.2%) | 10 (35.7%) | 0.005 |
| Renal replacement therapy | 131 (4.0%) | 2 (0.8%) | 0.012 |
| Extracorporeal membrane oxygenation | 18 (2.6%) | 0 (0.0%) | 0.681 |
| Left ventricular ejection fraction, mean (SD) | 57.8 (9.6) | 56.3 (9.8) | 0.138 |
| **In-hospital treatments** | | | |
| Vasoactive agents | 340 (10.4%) | 12 (4.9%) | 0.006 |
| Anticoagulant therapy | 2048 (62.5%) | 143 (58.8%) | 0.255 |
| Vitamin K antagonists | 57 (2.8%) | 4 (2.8%) | 0.992 |
| Directly acting oral anticoagulants† | 63 (3.1%) | 7 (4.9%) | 0.232 |
| Low-molecular-weight heparin | 1919 (93.7%) | 135 (94.4%) | 0.737 |
| Enoxaparin | 1702 (88.9%) | 129 (95.6%) | |
| Intensity | | | 0.005 |
| Standard prophylactic intensity | 1240 (66.2%) | 105 (79.5%) | |
| Intermediate intensity | 242 (12.9%) | 13 (9.8%) | |
| Full intensity | 391 (20.9%) | 14 (10.6%) | |
| Unfractionated heparin† | 57 (2.8%) | 4 (2.8%) | 0.992 |
| Aspirin | 420 (12.8%) | 34 (14.0%) | 0.001 |
| $P2Y_{12}$ inhibitors | 75 (2.3%) | 17 (7.0%) | 0.001 |
| Angiotensin-converting-enzyme inhibitors | 338 (10.3%) | 31 (12.8%) | 0.001 |
| Angiotensin II receptor blockers | 218 (6.7%) | 24 (9.9%) | 0.001 |
| Statins | 478 (14.6%) | 50 (20.6%) | 0.001 |
| β-blockers | 446 (13.6%) | 31 (12.8%) | 0.001 |
| Calcium channel blockers | 344 (10.5%) | 37 (15.2%) | 0.001 |
| Loop diuretics | 595 (18.2%) | 50 (20.6%) | 0.001 |
| Mineralocorticoid receptor antagonists | 94 (2.9%) | 8 (3.3%) | 0.001 |
| **COVID specific treatments** | | | |
| Lopinavir/Ritonavir | 1884 (52.7%) | - | - |
| Hydroxychloroquine | 2758 (77.1%) | - | - |
| Azithromycin | 1934 (54.1%) | - | - |
| Tocilizumab | 503 (14.1%) | - | - |
| Second monoclonal antibody | 60 (1.7%) | - | - |
| Corticosteroids | 1064 (29.7%) | - | - |
| Remdesivir | 120 (3.4%) | - | - |
| NSAIDs | 101 (2.8%) | - | - |
| Paracetamol | 2244 (62.7%) | - | - |

| | | | |
|---|---|---|---|
| **Biomarkers** | | | |
| Cardiac (peak value) | | | |
| Troponin I or T >ULN, n (%) | 435 (12.2%) | 12 (1.4%) | 0.001 |
| NT-proBNP or BNP >ULN, n (%) | 571 (15.9%) | 2 (0.2%) | 0.001 |
| Coagulation | | | |
| D-Dimer >ULN, n (%) | 2022 (56.5%) | 38 (4.5%) | 0.001 |
| Increased > 4x ULN | 933 (26.1%) | 14 (1.6%) | 0.001 |
| Prolonged PT at least 25%, | 62 (1.7%) | 8 (0.9%) | 0.097 |
| Fibrinogen lower than 2 g/dL | 172 (4.8%) | 12 (1.4%) | 0.632 |
| Blood count (lowest value) | | | |
| Hemoglobin (g/dL) | 12.3 (10.6-13.5) | 12.9 (10.7-13.9) | 0.084 |
| Platelets count lower than 100 × 109/L | 300 (8.4%) | 11 (1.3%) | 0.001 |
| Lymphocyte count ($10^9$/L) | 0.82 (0.5-1.3) | 1.00 (0.7-1.8) | 0.001 |
| Biochemistry (peak value) | | | |
| Creatinine (mg/dL) | 0.94 (0.76-1.26) | 0.86 (0.71-1.11) | 0.037 |
| C-reactive protein (mg/dL) | 14.6 (6.0-33.1) | 8.1 (1.5-16.2) | 0.001 |
| **Discharge medical treatments** | | | |
| Aspirin | 397 (11.1%) | 39 (4.6%) | 0.001 |
| $P2Y_{12}$ inhibitor | 69 (1.9%) | 17 (2.0%) | 0.888 |
| Angiotensin-converting-enzyme inhibitors | 421 (11.8%) | 37 (4.4%) | 0.001 |
| Angiotensin II receptor blockers | 318 (8.9%) | 26 (3.1%) | 0.001 |
| Angiotensin receptor neprilysin inhibitors | 11 (0.3%) | 1 (0.1%) | 0.339 |
| Statins | 625 (17.5%) | 60 (7.1%) | 0.001 |
| β-blocker | 471 (13.2%) | 41 (4.8%) | 0.001 |
| Calcium channel blockers | 331 (9.3%) | 24 (2.8%) | 0.001 |
| Loop diuretic | 272 (7.6%) | 28 (3.3%) | 0.001 |
| Mineralocorticoid receptor antagonists | 62 (1.7%) | 9 (1.1%) | 0.136 |
| Proton-pump inhibitors | 891 (24.9%) | 96 (11.3%) | 0.001 |
| Oral hypoglycemic agents | 342 (9.6%) | 26 (3.1%) | 0.001 |
| Insulin | 180 (5.0%) | 13 (1.5%) | 0.001 |
| Anticoagulant treatment | 946 (26.4%) | 48 (5.7%) | 0.001 |
| Nonsteroidal anti-inflammatory drugs | 50 (1.4%) | 47 (5.5%) | 0.001 |
| Corticosteroids | 336 (9.4%) | 30 (3.5%) | 0.001 |

[*]Patients in the COVID-19 cohort admitted for a CV or non-CV cause other than COVID-19 were diagnosed with COVID-19 during the hospitalization. [†]Anticoagulant therapy intensity was only computed for low-molecular-weight heparin. Data are shown as n (%), unless otherwise indicated. IQR=Interquartile Range; SD=standard deviation; BNP, B-type natriuretic peptide; ULN, Upper Limit of Normal. ULNs: Troponin I=40 ng/L or <45.2 ng/L according to essay type; Troponin T=14 ng/L; NT-proBNP and BNP= 84 pg/ml (males) and 146 pg/ml (females); D-dimer=500 ng/mL.

## S2 Table. One-year follow-up, vaccination status, and treatments.

| | COVID-19<br>N=3578 | Control<br>N=849 | P-value |
|---|---|---|---|
| In-hospital mortality | 521 (14.6%) | 11 (1.3%) | |
| Lost to follow-up | 59 (1.6%) | 28 (3.3%) | 0.014 |
| Follow-up done | 2998 (83.8%) | 810 (95.4%) | |
| **Vaccination status** | **N=1152** | **N=796** | |
| Yes | 950 (82.5%) | 664 (83.4%) | 0.584 |
| BNT162b2 | 567 (59.7%) | 455 (68.5%) | |
| mRNA-1273 | 228 (24.0%) | 147 (22.1%) | |
| ChAdOx1-S | 130 (13.7%) | 42 (6.3%) | |
| Ad26.COV.2 | 24 (2.5%) | 20 (3.0%) | |
| No | 202 (17.5%) | 132 (16.6%) | |
| **Follow-up treatments** | **N=2874** | **N=779** | |
| Aspirin | 424 (14.8%) | 51 (6.5%) | 0.001 |
| $P2Y_{12}$ Inhibitor | 56 (1.9%) | 12 (1.5%) | 0.454 |
| Angiotensin-converting-enzyme inhibitors | 443 (15.4%) | 64 (8.2%) | 0.001 |
| Angiotensin II receptor blockers | 361 (12.6%) | 49 (6.3%) | 0.001 |
| Angiotensin receptor neprilysin inhibitors | 26 (0.9%) | 5 (0.6%) | 0.477 |
| Statins | 715 (24.9%) | 94 (12.1%) | 0.001 |
| β-blocker | 471 (16.4%) | 63 (8.1%) | 0.001 |
| Calcium channel blocker | 344 (12.0%) | 32 (4.1%) | 0.001 |
| Loop diuretic | 276 (9.6%) | 36 (4.6%) | 0.001 |
| Mineralocorticoid receptor antagonists | 64 (2.2%) | 16 (2.1%) | 0.765 |
| Oral hypoglycemic agents | 380 (13.2%) | 43 (5.5%) | 0.001 |
| Insulin | 145 (5.0%) | 22 (2.8%) | 0.008 |
| Anticoagulant treatment | 263 (9.2%) | 31 (4.0%) | 0.001 |
| Corticosteroids | 134 (4.7%) | 25 (3.2%) | 0.077 |

Data are shown as n (%)

**S3 Table. Types of cardiovascular death according to the Academic Research Consortium-2.**

| | COVID-19 N=3578 | Control N=849 | Hazard ratio* (95%CI) | P-value* |
|---|---|---|---|---|
| **Entire study (0–365 days)** | | | | |
| Cardiovascular death | 51 (1.4) | 7 (0.8) | 1.28 (0.56-2.91) | 0.555 |
| Death caused by acute myocardial infarction | 5 (9.8) | 1 (14.3) | - | |
| Death caused by sudden cardiac | 3 (5.9) | 2 (28.6) | - | |
| Death resulting from heart failure | 16 (31.4) | 4 (57.1) | - | 0.419[¥] |
| Death caused by stroke | 3 (5.9) | 0 | - | |
| Death caused by CV procedures | 1 (2.0) | 0 | - | |
| Death result from other CV causes | 23 (45.1) | 0 | - | |
| Arterial ischemia | 2 (8.3) | 0 | - | |
| Non-cardiovascular death | 585 (16.3) | 27 (3.2) | 3.22 (2.18-4.76) | 0.001 |
| Death caused by infection (includes sepsis) | 474 (81.0) | 8 (29.6) | - | |
| Death caused by other non-CV organ failure | 5 (0.9) | 2 (7.4) | - | |
| Death resulting from gastrointestinal causes | 4 (0.7) | 0 (0.0) | - | |
| Death resulting from malignancy | 24 (4.1) | 14 (51.9) | - | 0.001[¥] |
| Death resulting from other non-CV cause | 4 (0.7) | 0 (0.0) | - | |
| Death resulting from pulmonary causes | 49 (8.4) | 1 (3.7) | - | |
| Death resulting from accident/trauma | 1 (0.2) | 0 | | |
| Undetermined cause of death | 24 (4.1) | 2 (7.4) | - | |
| **Acute phase (0–30 days)** | | | | |
| Cardiovascular death | 32 (0.9) | 1 (0.1) | 1.35 (0.18-10.22) | 0.770 |
| Death caused by acute myocardial infarction | 4 (12.5) | 1 (100.0) | | |
| Death caused by sudden cardiac | 1 (3.1) | | | |
| Death resulting from heart failure | 5 (15.6) | 0 | - | |
| Death caused by stroke | 2 (6.3) | 0 | - | 0.364[¥] |
| Death result from other CV causes | 20 (62.5) | 0 | - | |
| Arterial ischemia | 2 (8.3) | - | - | |
| Non-cardiovascular death | 468 (13.1) | 14 (1.6) | 1.67 (0.93-2.97) | 0.085 |
| Death caused by infection (includes sepsis) | 414 (88.5) | 7 (50.0) | - | |
| Death caused by other non-CV organ failure | 1 (0.2) | 1 (7.1) | | |
| Death resulting from gastrointestinal causes | 2 (0.4) | 0 | | |
| Death resulting from malignancy | 1 (0.2) | 5 (35.7) | | 0.001[¥] |
| Death resulting from other non-CV | 1 (0.2) | 0 | - | |
| Death resulting from pulmonary causes | 44 (9.4) | 0 | - | |
| Death resulting from accident/trauma | 1 (0.2) | 0 | - | |
| Undetermined cause of death | 4 (0.9) | 1 (7.1) | - | |
| **Post-acute phase (31–365 days)** | | | | |
| Cardiovascular death | 19 (0.6) | 6 (0.7) | 0.67 (0.25-1.80) | 0.425 |
| Death caused by acute myocardial infarction | 1 (5.3) | 0 (0.0) | - | |
| Death caused by sudden cardiac | 2 (10.5) | 2 (33.3) | - | |
| Death resulting from heart failure | 11 (57.9) | 4 (66.7) | - | 0.790[¥] |
| Death caused by stroke | 1 (5.3) | 0 (0.0) | - | |
| Death caused by CV procedures | 1 (5.3) | 0 (0.0) | - | |
| Death result from other CV causes | 3 (15.8) | 0 (0.0) | - | |
| Non-cardiovascular death | 117 (3.9) | 13 (1.6) | 1.72 (0.96-3.10) | 0.070 |
| Death caused by infection (includes sepsis) | 60 (51.3) | 1 (7.7) | - | |
| Death caused by other non-CV organ failure | 4 (3.4) | 1 (7.7) | - | |
| Death resulting from gastrointestinal causes | 2 (1.7) | 0 (0.0) | - | |
| Death resulting from malignancy | 23 (19.7) | 9 (69.2) | - | 0.003[¥] |
| Death resulting from other non-CV cause | 3 (2.6) | 0 (0.0) | - | |
| Death resulting from pulmonary causes | 5 (4.3) | 1 (7.7) | - | |
| Undetermined cause of death | 20 (17.1) | 1 (7.7) | - | |

Data are shown as n (%), unless otherwise indicated. *Cox proportional hazards regression analysis adjusted by sex, age, smoking status, diabetes, previous chronic kidney disease, previous PCI, previous heart failure, dementia, cancer, and organ transplant. [¥]Fisher's exact test. CV=cardiovascular; 95%CI=95% confidence interval.

**S4 Table. Pre-specified sensibility analyses.**

| | COVID-19 N=3578 | Control N=849 | Hazard Ratio* (95%CI) | P-value |
|---|---|---|---|---|
| **Competing risk analysis[1]** | | | | |
| **Entire study (0–365 days)** | N=3578 | N=849 | | |
| All-cause death | 636 (17.8) | 34 (4.0) | | |
| Cardiovascular death | 51 (1.4) | 7 (0.8) | 1.07 (0.51-2.25) | 0.853 |
| Myocardial infarction | 58 (1.6) | 7 (0.8) | 1.35 (0.57-3.18) | 0.494 |
| Stroke or transient ischemic attack | 39 (1.1) | 2 (0.2) | 3.09 (0.71-13.41) | 0.132 |
| Transient ischemic attack | 10 (0.3) | 1 (0.1) | 1.17 (0.16-8.69) | 0.875 |
| Ischemic stroke | 24 (0.7) | 0 | - | - |
| Hemorrhagic stroke | 5 (0.1) | 1 (0.1) | 0.97 (0.09-10.74) | 0.980 |
| Systemic arterial embolism | 11 (0.3) | 0 | - | - |
| Arterial thrombotic event | 90 (2.5) | 7 (0.8) | 2.13 (0.94-4.85) | 0.071 |
| Venous thromboembolism | 134 (3.7) | 3 (0.4) | 5.75 (1.84-17.95) | 0.003 |
| Deep vein thrombosis | 65 (1.8) | 0 | - | - |
| Pulmonary Embolism | 93 (2.6) | 3 (0.4) | 9.02 (2.89-28.17) | 0.001 |
| Major bleeding or blood transfusion | 249 (7.0) | 16 (1.9) | 1.12 (0.59-2.10) | 0.733 |
| Major bleeding | 102 (2.9) | 4 (0.5) | 0.90 (0.32-2.51) | 0.842 |
| Red blood cell transfusion | 227 (6.3) | 15 (1.8) | 1.15 (0.60-2.23) | 0.673 |
| Serious cardiac arrhythmias | 90 (2.5) | 5 (0.6) | 3.16 (1.24-8.05) | 0.016 |
| Major adverse cardiovascular event | 131 (3.7) | 13 (1.5) | 1.60 (0.90-2.84) | 0.110 |
| Adverse cardiovascular events | 348 (9.7) | 26 (3.1) | 2.45 (1.65-3.64) | 0.001 |
| **Acute phase (0–30 days)** | | | | |
| All-cause death | 500 (14.0) | 15 (1.8) | | |
| Cardiovascular death | 32 (0.9) | 1 (0.1) | 1.36 (0.17-10.71) | 0.773 |
| Myocardial infarction | 46 (1.3) | 3 (0.4) | 2.43 (0.70-8.51) | 0.164 |
| Stroke or transient ischemic attack | 28 (0.8) | 2 (0.2) | 2.22 (0.50-9.79) | 0.292 |
| Transient ischemic attack | 5 (0.1) | 1 (0.1) | 0.65 (0.09-4.87) | 0.673 |
| Ischemic stroke | 21 (0.6) | 0 | - | - |
| Hemorrhagic stroke | 2 (0.1) | 1 (0.1) | 0.35 (0.02-5.91) | 0.465 |
| Systemic arterial embolism | 9 (0.3) | 0 | - | - |
| Arterial thrombotic event | 74 (2.1) | 3 (0.4) | 4.10 (1.22-13.78) | 0.022 |
| Venous thromboembolism | 109 (3.0) | 0 | - | - |
| Deep vein thrombosis | 47 (1.3) | 0 | - | - |
| Pulmonary Embolism | 80 (2.2) | 0 | - | - |
| Major bleeding or blood transfusion | 241 (6.7) | 16 (1.9%) | 1.08 (0058-2.03) | 0.806 |
| Major bleeding | 79 (2.2) | 3 (0.4%) | 0.90 (0.28-2.83) | 0.854 |
| Red blood cell transfusion | 227 (6.3) | 15 (1.8%) | 1.15 (0.60-2.23) | 0.673 |
| Serious cardiac arrhythmias | 73 (2.0) | 2 (0.2%) | 5.75 (1.32-25.11) | 0.020 |
| Major adverse cardiovascular event | 96 (2.7) | 4 (0.5%) | 3.65 (1.28-10.36) | 0.015 |
| Adverse cardiovascular events | 251 (7.0) | 6 (0.7%) | 7.29 (3.16-16.78) | 0.001 |
| **Post-acute phase (31–365 days)** | **N=3022** | **N=806** | | |
| All-cause death | 145 (4.8) | 27 (3.3) | | |
| Cardiovascular death | 19 (0.6) | 6 (0.7) | 0.53 (0.25-1.12) | 0.098 |
| Myocardial infarction | 12 (0.4) | 4 (0.5) | 0.57 (0.15-2.14) | 0.409 |
| Stroke or transient ischemic attack | 11 (0.4) | 0 | - | - |
| Transient ischemic attack | 5 (0.2) | 0 | - | - |
| Ischemic stroke | 3 (0.1) | 0 | - | - |
| Hemorrhagic stroke | 3 (0.1) | 0 | - | - |
| Systemic arterial embolism | 2 (0.1) | 0 | - | - |
| Arterial thrombotic event | 16 (0.5) | 4 (0.5) | 0.68 (0.19-2.41) | 0.554 |
| Venous thromboembolism | 25 (0.8) | 3 (0.4) | 1.79 (0.56-5.66) | 0.321 |
| Deep vein thrombosis | 18 (0.6) | 0 | - | - |
| Pulmonary Embolism | 13 (0.4) | 3 (0.4) | 0.90 (0.26-3.15) | 0.871 |
| Heart failure hospitalization | 43 (1.2) | 14 (1.6) | 0.67 (0.34-1.33) | 0.254 |

| | COVID-19 N=3578 | Control N=849 | Hazard Ratio* (95%CI) | P-value |
|---|---|---|---|---|
| Major bleeding or blood transfusion | 8 (0.3) | 0 | - | - |
| Major bleeding | 23 (0.8) | 1 (0.1) | 1.07 (0.11-10.3) | 0.954 |
| Red blood cell transfusion | 0 | 0 | - | - |
| Serious cardia arrhythmias | 17 (0.6) | 3 (0.4) | 1.58 (0.46-5.44) | 0.467 |
| Major adverse cardiovascular event | 35 (1.2) | 9 (1.1) | 0.81 (0.40-1.64) | 0.551 |
| Adverse cardiovascular events | 97 (3.2) | 20 (2.5) | 1.12 (0.69-1.81) | 0.657 |
| **Adjudicated-only population[2]** | | | | |
| Adjudicated deaths, n (%) | 531 (83.5) | 33 (97.1) | - | 0.001 |
| **Entire study (0–365 days)** | **N=3473** | **N=848** | | |
| Cardiovascular death | 25 (0.7) | 7 (0.8) | 0.73 (0.29-1.83) | 0.461 |
| **Acute phase (0–30 days)** | | | | |
| Cardiovascular death | 14 (0.4) | 1 (0.1) | 0.56 (0.07-4.82) | 0.276 |
| **Post-acute phase (31–365 days)** | **N=2917** | **N=805** | | |
| Cardiovascular death | 11 (0.4) | 6 (0.7) | 0.36 (0.12-1.07) | 0.066 |

[1]Competing risk survival analysis methods were applied to estimate the cumulative incidence of the reported outcome with all-cause death as competing risk event.
[2]Adjudicated-only population was defined as the population with available source documents that underwent independent event adjudication.
*Cox proportional hazards regression analysis adjusted by sex, age, smoking status, diabetes, previous chronic kidney disease, previous PCI, previous heart failure, dementia, cancer, and organ transplant.

## S5 Table. Multivariable predictors of adverse cardiovascular events during the post-acute phase in patients with COVID-19.

| Long-term adverse cardiovascular events Events=97 and N=3022 | Events n (%) | Events n (%) | Univariate* HR (95%CI); P | Multivariate¥ HR (95%CI); P |
|---|---|---|---|---|
| **Baseline characteristics** | | | | |
| Age (yr) | - | - | 1.04 (1.02-1.05); 0.001 | Removed |
| Sex (males vs. females) | 66 (3.9) | 31 (2.4) | 1.67 (1.09-2.56); 0.019 | Removed |
| Body mass index (kg/m$^2$) | - | - | 1.03 (0.98-1.09); 0.237 | - |
| Smoking status (active or former vs. never) | 44 (5.7) | 53 (2.4) | 2.48 (1.66-3.70); 0.001 | 1.78 (1.18-2.70); 0.007 |
| Diabetes mellitus | 32 (5.8) | 65 (2.6) | 2.31 (1.51-3.52); 0.001 | Removed |
| Hypertension | 69 (5.5) | 28 (1.6) | 3.60 (2.32-5.59); 0.001 | 1.85 (1.15-2.99); 0.012 |
| Hypercholesterolemia | 41 (4.5) | 56 (2.7) | 1.75 (1.17-2.62); 0.006 | Removed |
| Chronic Kidney Disease | 20 (7.4) | 77 (2.8) | 2.81 (1.72-4.59); 0.001 | Removed |
| Atrial fibrillation | 28 (12.0) | 69 (2.5) | 5.19 (3.34-8.05); 0.001 | 2.27 (1.33-3.86); 0.003 |
| Premature Coronary Artery Disease | 2 (4.8) | 95 (3.2) | 1.56 (0.39-6.34); 0.532 | - |
| Stroke or transient ischemic attack | 10 (8.1) | 87 (3.0) | 2.94 (1.53-5.66); 0.001 | Removed |
| Myocardial infarction | 13 (9.1) | 84 (2.9) | 3.29 (1.83-5.90); 0.001 | Removed |
| Percutaneous coronary intervention | 10 (6.3) | 87 (3.0) | 2.16 (1.12-4.15); 0.021 | Removed |
| Coronary artery bypass grafting | 2 (5.4) | 95 (3.2) | 1.73 (0.43-7.01); 0.444 | - |
| Peripheral vascular disease | 6 (5.0) | 91 (3.1) | 1.70 (0.74-3.88); 0.210 | - |
| Chronic obstructive pulmonary disease or asthma | 15 (5.3) | 82 (3.0) | 1.82 (1.05-3.15); 0.034 | Removed |
| History of pneumonia | 3 (2.7) | 94 (3.2) | 0.86 (0.27-2.71); 0.800 | - |
| Heart Failure | 19 (18.1) | 78 (2.7) | 7.79 (4.72-12.87); 0.001 | 2.27 (1.20-4.28); 0.011 |
| Pulmonary hypertension | 9 (17.0) | 88 (3.0) | 6.62 (3.33-13.15); 0.001 | 2.48 (1.61-5.31); 0.019 |
| Venous thromboembolism | 2 (4.3) | 95 (3.2) | 1.39 (0.34-5.64); 0.644 | - |
| Major bleeding | 3 (5.5) | 94 (3.2) | 1.80 (0.57-5.67); 0.318 | - |
| Dementia | 4 (5.1) | 93 (3.2) | 1.69 (0.62-4.60); 0.303 | - |
| Cancer (active or past vs. no history) | 23 (7.2) | 74 (2.7) | 2.74 (1.71-4.37); 0.001 | 2.16 (1.33-3.52); 0.002 |
| Organ transplant | 3 (6.8) | 94 (3.2) | 2.31 (0.73-7.30); 0.153 | - |
| Moderate or severe valvular heart disease | 16 (18.4) | 81 (2.8) | 7.8 (4.56-13.34); 0.001 | 2.57 (1.34-4.94); 0.005 |
| Valve repair or replacement | 5 (11.9) | 92 (3.1) | 4.21 (1.71-10.35); 0.002 | Removed |
| Functional health status (dependent vs. independent) | 22 (7.3) | 75 (2.8) | 2.85 (1.77-4.59); 0.001 | 2.16 (1.29-3.60); 0.003 |
| **Previous medical treatments** | | | | |
| Aspirin | 19 (4.4) | 78 (3.0) | 1.50 (0.91-2.47); 0.115 | - |
| P2Y$_{12}$ inhibitor | 3 (5.3) | 94 (3.2) | 1.69 (0.54-5.34); 0.370 | - |
| Angiotensin-converting-enzyme inhibitors | 19 (4.1) | 78 (3.1) | 1.36 (0.83-2.25); 0.227 | - |
| Angiotensin II receptor blockers | 18 (4.5) | 79 (3.0) | 1.52 (0.91-2.53); 0.111 | - |
| Angiotensin receptor neprilysin inhibitors | 1 (8.3) | 96 (3.2) | 2.64 (0.37-18.92); 0.335 | - |
| Statins | 33 (4.6) | 64 (2.8) | 1.71 (1.13-2.61); 0.012 | Removed |
| β-blocker | 29 (6.7) | 68 (2.6) | 2.61 (1.69-4.03); 0.001 | Removed |
| Calcium channel blockers | 18 (5.4) | 79 (2.9) | 1.89 (1.13-3.15); 0.015 | Removed |
| Loop diuretic | 28 (10.5) | 69 (2.5) | 4.59 (2.95-7.11); 0.001 | Removed |
| Mineralocorticoid receptor antagonists | 4 (6.3) | 93 (3.1) | 2.14 (0.79-5.83); 0.136 | - |
| Proton-pump inhibitors | 40 (5.2) | 57 (2.5) | 2.16 (1.44-3.24); 0.001 | Removed |
| Oral hypoglycemic agents | 24 (6.0) | 73 (2.8) | 2.22 (1.40-3.52); 0.001 | Removed |
| Insulin | 8 (5.8) | 89 (3.1) | 1.98 (0.96-4.08); 0.065 | - |
| Anticoagulant treatment | 25 (11.1) | 72 (2.6) | 4.71 (2.98-7.42); 0.001 | Removed |
| Nonsteroidal anti-inflammatory drugs | 3 (4.8) | 94 (3.2) | 0.67 (0.21-2.11); 0.490 | - |
| Paracetamol | 22 (5.1) | 75 (2.9) | 1.82 (1.13-2.92); 0.014 | Removed |
| **Hospitalization characteristics** | | | | |
| Hospitalization | 93 (3.4) | 4 (1.4) | 2.53 (0.93-6.87); 0.069 | - |
| Length of stay (days) | - | - | 1.02 (1.01-1.03); 0.001 | Removed |
| Intensive Care Unit | 33 (6.1) | 64 (2.6) | 2.50 (1.64-3.81); 0.001 | 2.32 (1.35-3.99); 0.001 |
| Length of stay (days) | - | - | 1.03 (1.02-1.03); 0.001 | Removed |
| Invasive mechanical ventilation | 22 (6.8) | 75 (2.8) | 2.64 (1.64-4.25); 0.001 | Removed |

Adverse cardiovascular events are defined as the composite of cardiovascular death, any venous or arterial thrombotic event, heart failure hospitalization, or any serious arrhythmia. Post-acute phase comprised from 31-365 days of follow-up.

*Cox proportional hazards regression analysis adjusted by each variable.

¥Cox proportional hazards regression analysis adjusted by the significant variables in univariate analysis using a backward selection model.

HR=hazard ratio; 95%CI=95% confidence interval, P=p-value.

**S1 Fig. CV- COVID 19 registry flow chart.**

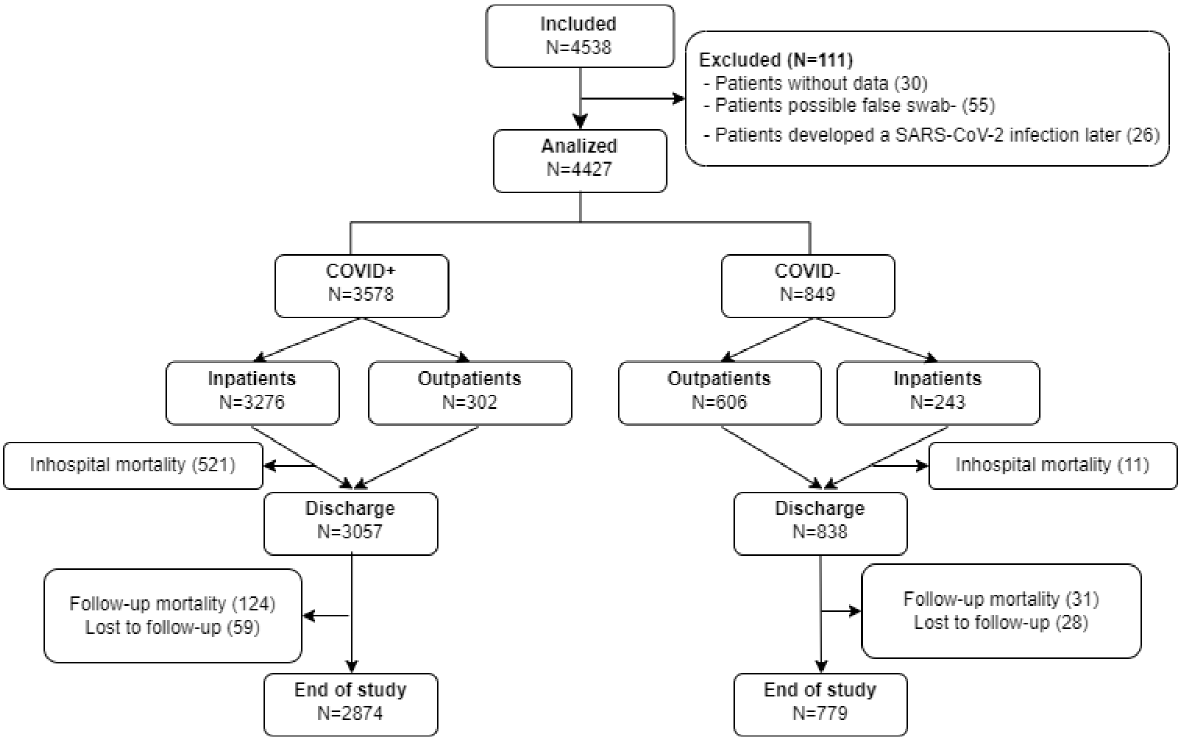


SARS-CoV-2: severe acute respiratory syndrome coronavirus 2
COVID-19: Coronavirus disease 2019

**S2 Fig. Cumulative incidence of cardiovascular outcomes in the COVID-19 and control cohorts during the entire study period (0–365 days).**

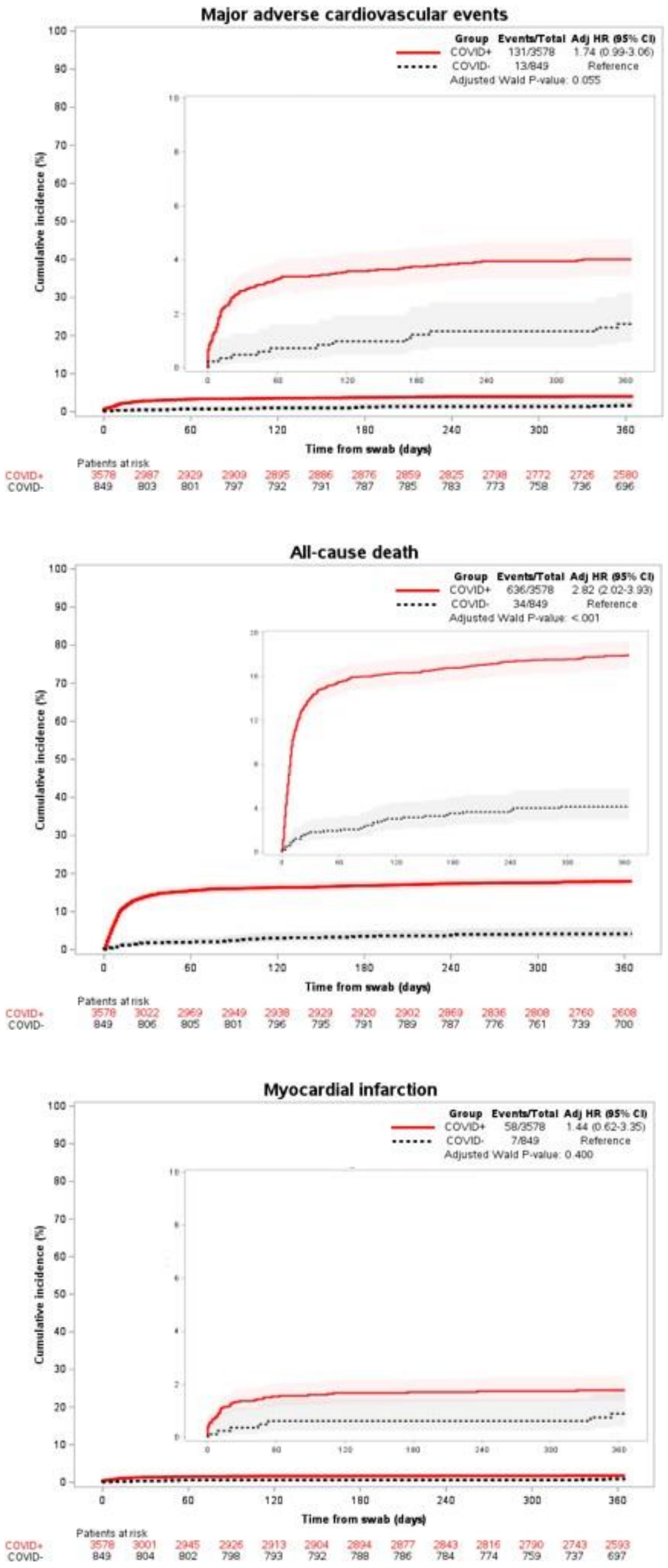

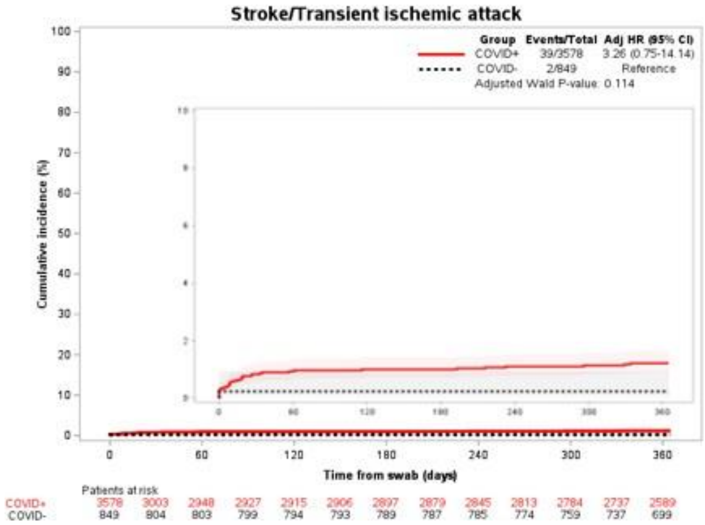

Stroke/Transient ischemic attack
Group Events/Total Adj HR (95% CI)
COVID+ 39/3578 3.26 (0.75-14.14)
COVID- 2/849 Reference
Adjusted Wald P-value: 0.114
Cumulative incidence (%)
Time from swab (days)
Patients at risk
COVID+ 3578 3003 2948 2927 2915 2906 2897 2879 2845 2813 2784 2737 2589
COVID- 849 804 803 799 794 793 789 787 785 774 759 737 699


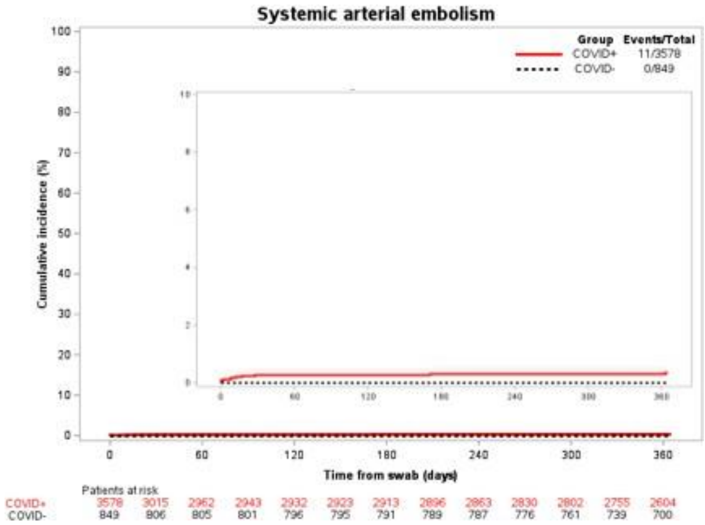

Systemic arterial embolism
Group Events/Total
COVID+ 11/3578
COVID- 0/849
Cumulative incidence (%)
Time from swab (days)
Patients at risk
COVID+ 3578 3015 2962 2943 2932 2923 2913 2896 2863 2830 2802 2755 2604
COVID- 849 806 805 801 796 795 791 789 787 776 761 739 700


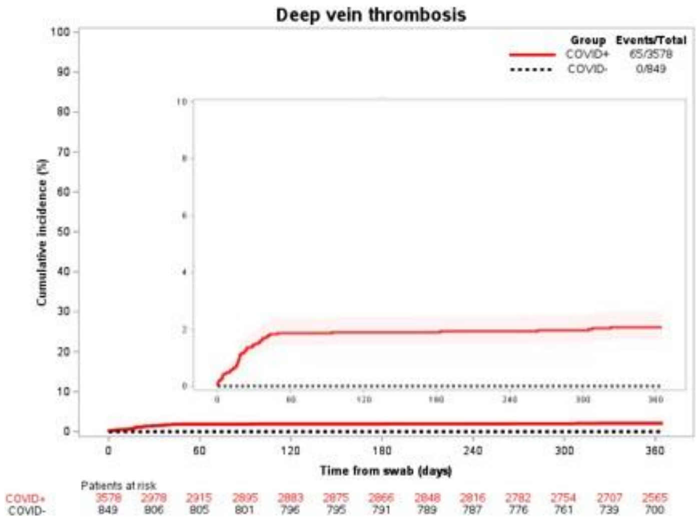

Deep vein thrombosis
Group Events/Total
COVID+ 65/3578
COVID- 0/849
Cumulative incidence (%)
Time from swab (days)
Patients at risk
COVID+ 3578 2978 2915 2895 2883 2875 2866 2848 2816 2782 2754 2707 2565
COVID- 849 806 805 801 796 795 791 789 787 776 761 739 700

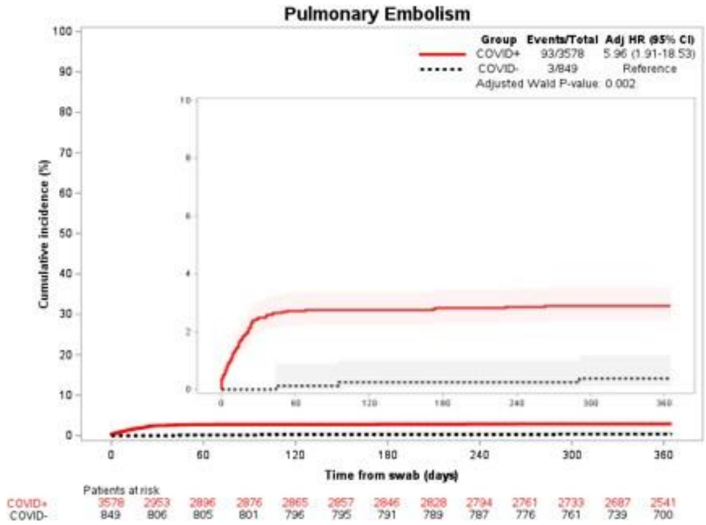
Pulmonary Embolism
Group Events/Total Adj HR (95% CI)
COVID+ 93/3578 5.96 (1.91-18.53)
COVID- 3/849 Reference
Adjusted Wald P-value: 0.002
Cumulative incidence (%)
Time from swab (days)
Patients at risk
COVID+ 3578 2953 2896 2876 2865 2857 2846 2828 2794 2761 2733 2687 2541
COVID- 849 806 805 801 796 795 791 789 787 776 761 739 700

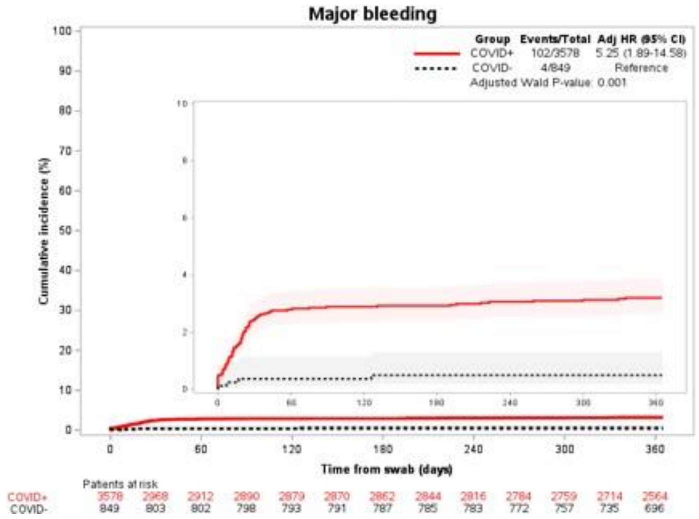
Major bleeding
Group Events/Total Adj HR (95% CI)
COVID+ 102/3578 5.25 (1.89-14.58)
COVID- 4/849 Reference
Adjusted Wald P-value: 0.001
Cumulative incidence (%)
Time from swab (days)
Patients at risk
COVID+ 3578 2968 2912 2890 2879 2870 2862 2844 2816 2784 2759 2714 2564
COVID- 849 803 802 798 793 791 787 785 783 772 757 735 696

**S3 Fig. Cumulative incidence of cardiovascular outcomes in the COVID-19 and control cohorts during the entire post-acute phase (31–365 days).**

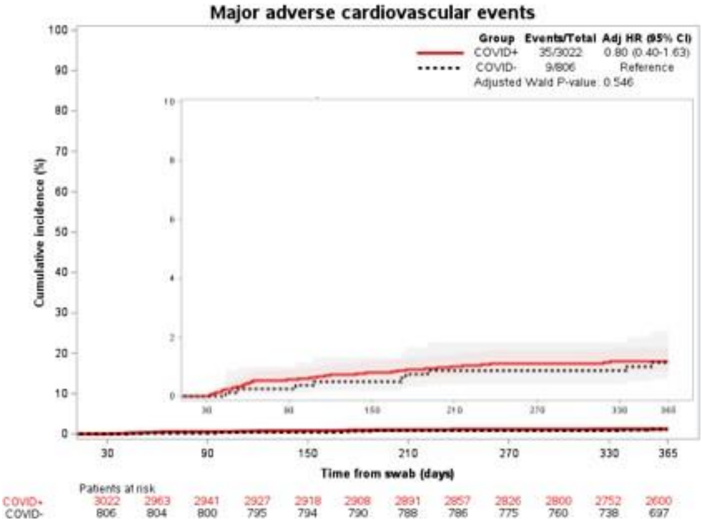


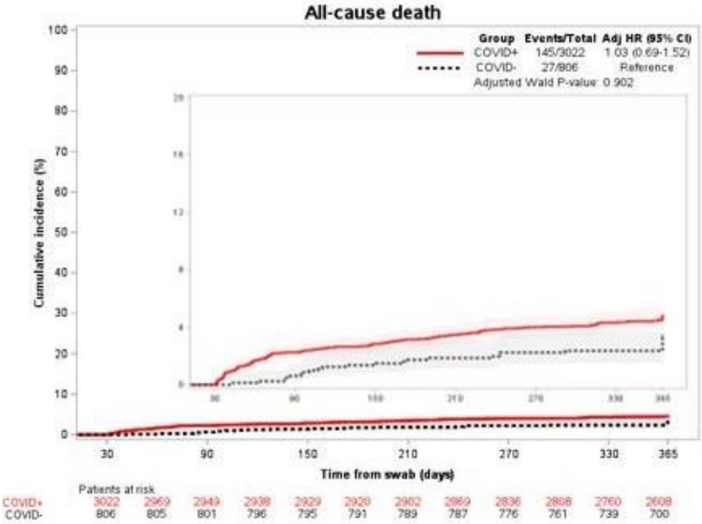


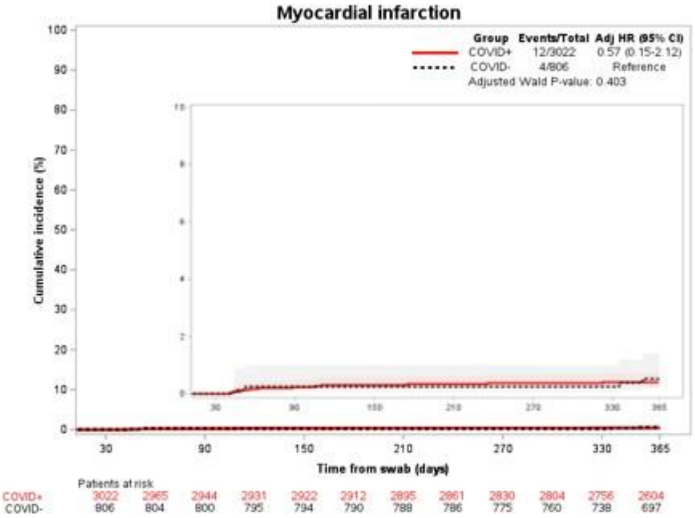

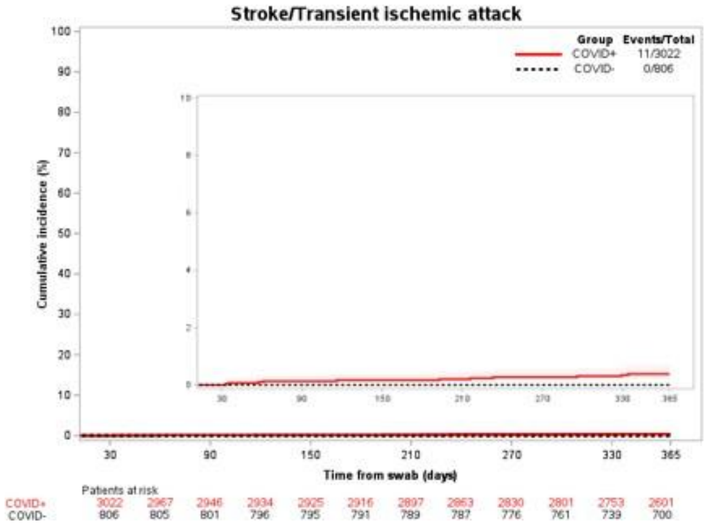
Stroke/Transient ischemic attack
Group Events/Total
COVID+ 11/3022
COVID- 0/806
Cumulative incidence (%)
Time from swab (days)
Patients at risk
COVID+ 3022 2967 2946 2934 2925 2916 2897 2863 2830 2801 2753 2601
COVID- 806 805 801 796 795 791 789 787 776 761 739 700

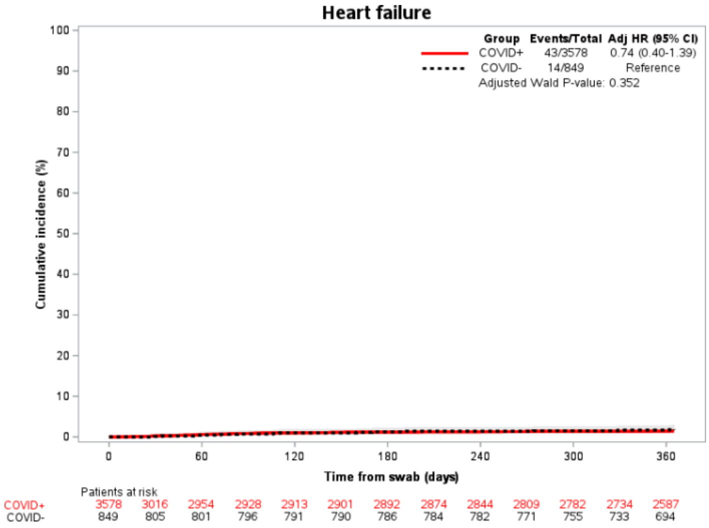
Heart failure
Group Events/Total Adj HR (95% CI)
COVID+ 43/3578 0.74 (0.40-1.39)
COVID- 14/849 Reference
Adjusted Wald P-value: 0.352
Cumulative incidence (%)
Time from swab (days)
Patients at risk
COVID+ 3578 3016 2954 2928 2913 2901 2892 2874 2844 2809 2782 2734 2587
COVID- 849 805 801 796 791 790 786 784 782 771 755 733 694

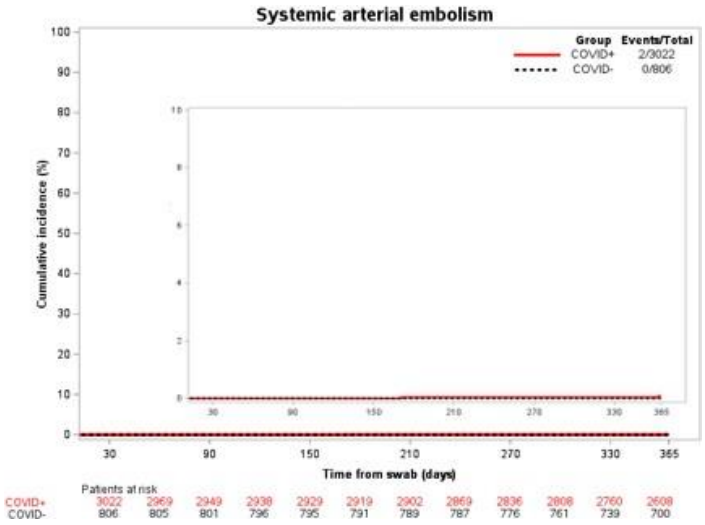
Systemic arterial embolism
Group Events/Total
COVID+ 2/3022
COVID- 0/806
Cumulative incidence (%)
Time from swab (days)
Patients at risk
COVID+ 3022 2969 2949 2938 2929 2919 2902 2869 2836 2808 2760 2608
COVID- 806 805 801 796 795 791 789 787 776 761 739 700

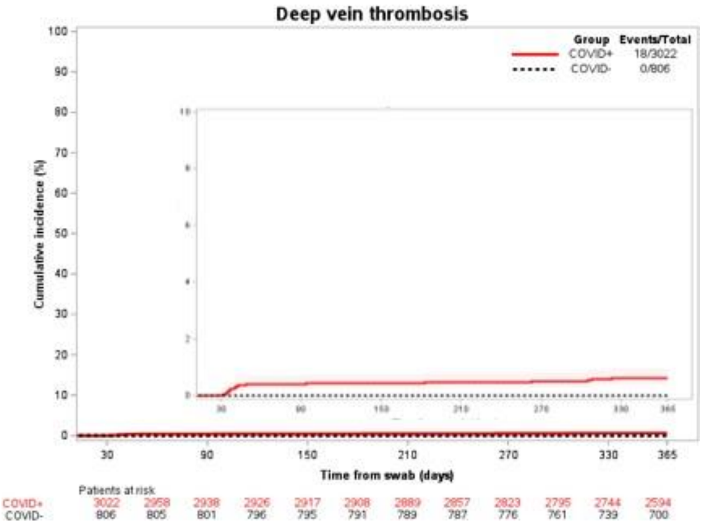
Deep vein thrombosis
Group Events/Total
COVID+ 18/3022
COVID- 0/806
Cumulative incidence (%)
Time from swab (days)
Patients at risk
COVID+ 3022 2958 2938 2926 2917 2908 2889 2857 2823 2795 2744 2594
COVID- 806 805 801 796 795 791 789 787 776 761 739 700

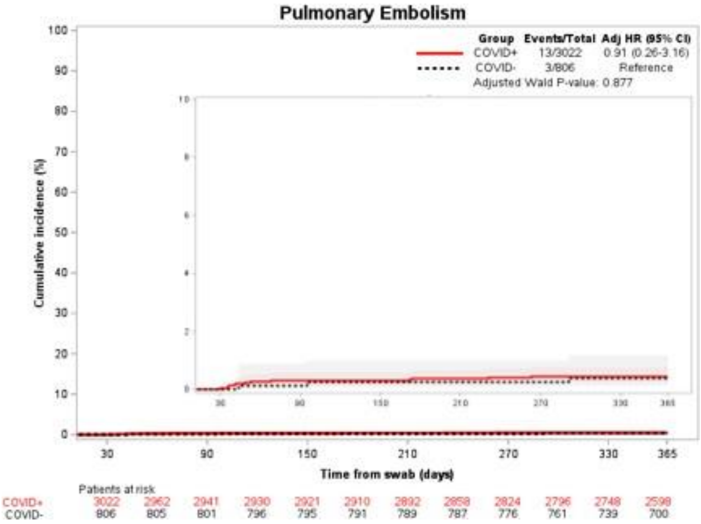
Pulmonary Embolism
Group Events/Total Adj HR (95% CI)
COVID+ 13/3022 0.91 (0.26-3.16)
COVID- 3/806 Reference
Adjusted Wald P-value: 0.877
Cumulative incidence (%)
Time from swab (days)
Patients at risk
COVID+ 3022 2962 2941 2930 2921 2910 2892 2858 2824 2796 2748 2598
COVID- 806 805 801 796 795 791 789 787 776 761 739 700

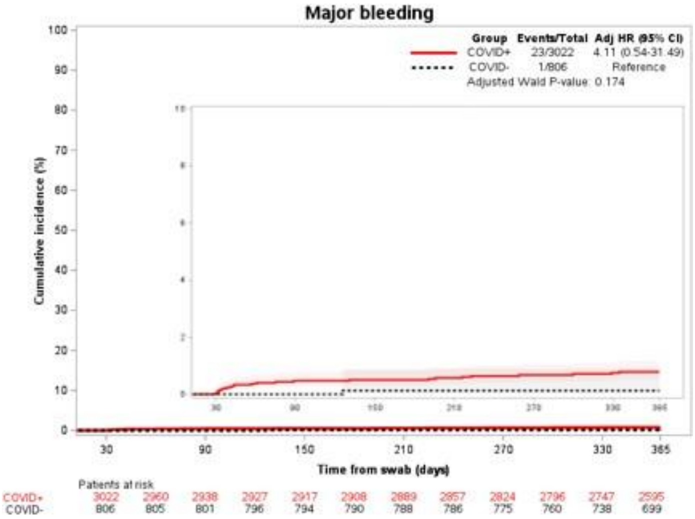
Major bleeding
Group Events/Total Adj HR (95% CI)
COVID+ 23/3022 4.11 (0.54-31.49)
COVID- 1/806 Reference
Adjusted Wald P-value: 0.174
Cumulative incidence (%)
Time from swab (days)
Patients at risk
COVID+ 3022 2960 2938 2927 2917 2908 2889 2857 2824 2796 2747 2595
COVID- 806 805 801 796 794 790 788 786 775 760 738 699

**References**


1. Arevalos V, Ortega-Paz L, Fernandez-Rodriguez D, Alfonso Jimenez-Diaz V, Rius JB, Campo G, Rodriguez-Santamarta M, de Prado AP, Gomez-Menchero A, Diaz Fernandez JF, et al. Long-term effects of coronavirus disease 2019 on the cardiovascular system, CV COVID registry: A structured summary of a study protocol. *PLoS One*. 2021;16:e0255263. doi: 10.1371/journal.pone.0255263
2. Garcia-Garcia HM, McFadden EP, Farb A, Mehran R, Stone GW, Spertus J, Onuma Y, Morel MA, van Es GA, Zuckerman B, et al. Standardized End Point Definitions for Coronary Intervention Trials: The Academic Research Consortium-2 Consensus Document. *Circulation*. 2018;137:2635-2650. doi: 10.1161/CIRCULATIONAHA.117.029289
3. Thygesen K, Alpert JS, Jaffe AS, Chaitman BR, Bax JJ, Morrow DA, White HD, Executive Group on behalf of the Joint European Society of Cardiology /American College of Cardiology /American Heart Association /World Heart Federation Task Force for the Universal Definition of Myocardial I. Fourth Universal Definition of Myocardial Infarction (2018). *Circulation*. 2018;138:e618-e651. doi: 10.1161/CIR.0000000000000617
4. Mehran R, Rao SV, Bhatt DL, Gibson CM, Caixeta A, Eikelboom J, Kaul S, Wiviott SD, Menon V, Nikolsky E, et al. Standardized bleeding definitions for cardiovascular clinical trials: a consensus report from the Bleeding Academic Research Consortium. *Circulation*. 2011;123:2736-2747. doi: 10.1161/CIRCULATIONAHA.110.009449
5. Charlson M, Szatrowski TP, Peterson J, Gold J. Validation of a combined comorbidity index. *J Clin Epidemiol*. 1994;47:1245-1251. doi: 10.1016/0895-4356(94)90129-5
6. Mahoney FI, Barthel DW. Functional Evaluation: The Barthel Index. *Md State Med J*. 1965;14:61-65.

CV COVID-19 Registry PID 162

Codebook

## Data Dictionary Codebook

25.08.2022 13:18

Collapse all instruments

| # | Variable / Field Name | Field Label<br>*Field Note* | Field Attributes (Field Type, Validation, Choices, Calculations, etc.) |
|---|---|---|---|
| Instrument: **Center & Patient ID** (center_patient_id) | | | Collapse |
| 1 | record_id | Record ID | text |
| 2 | nhc | Number of clinical history<br>*Of the hospitalization center* | text, Required, Identifier |
| 3 | dob | Date of Birth | text (date_dmy, Max: 2020-02-24), Required |
| 4 | sex | Gender | radio, Required<br>1 Men<br>2 Women |
| 5 | coordinating_center | Did the patient was enrolled in the coordinating center? | yesno, Required<br>1 Yes<br>0 No |
| 6 | postal_code | Postal code (código postal)<br>*ONLY FOR BARCELONA CENTERS (SOLO PARA CENTROS DE BARCELONA)* | text (number) |
| 7 | center_patient_id_complete | Section Header: *Form Status*<br>Complete? | dropdown<br>0 Incomplete<br>1 Unverified<br>2 Complete |
| Instrument: **Baseline** (baseline) | | | Collapse |
| 8 | base_height_cm | Section Header: *BASELINE - CLINICAL CHARACTERISTICS (If any characteristic is unknown, use the NO option)*<br>Height (cm)<br>*If unknown use 999.* | text (number, Min: 50, Max: 999), Required |
| 9 | base_weight_kg | Weight (Kg)<br>*If unknown use 999.* | text (number, Min: 40, Max: 999), Required |
| 10 | base_smoking | Previous smoking status | radio, Required<br>0 Never<br>1 Current<br>2 Former |
| 11 | base_diabetes | Previous Diabetes mellitus | yesno, Required<br>1 Yes<br>0 No |
| 12 | base_hypertension | Previous Hypertension | yesno, Required<br>1 Yes<br>0 No |
| 13 | base_hypercholesterolemia | Previous Hypercholesterolemia | yesno, Required<br>1 Yes<br>0 No |
| 14 | base_ckd | Previous Chronic Kidney Disease<br>*For eGFR calculation: https://www.mdcalc.com/ckd-epi-equations-glomerular-filtration-rate-gfr* | radio, Required<br>1 No<br>2 Yes - eGFR < 60 ml/min/1.73m2<br>3 Dialysis |

| | | | |
|---|---|---|---|
| 15 | base_atrial_fib | Previous Atrial fibrillation or Atrial flutter | yesno, Required<br>1 Yes<br>0 No |
| 16 | base_family_hist_cad | Family History of Premature Coronary Artery Disease<br>*< 55 years in first-degree male relatives and < 65 years in female relatives.* | yesno, Required<br>1 Yes<br>0 No |
| 17 | base_stroke_tia | Previous Stroke or transient ischemic attack | yesno, Required<br>1 Yes<br>0 No |
| 18 | base_mi | Previous Myocardial infarction | yesno, Required<br>1 Yes<br>0 No |
| 19 | base_pci | Previous Percutaneous coronary intervention | yesno, Required<br>1 Yes<br>0 No |
| 20 | base_cabg | Previous Coronary artery bypass grafting (CABG) | yesno, Required<br>1 Yes<br>0 No |
| 21 | base_pvd | Previous Peripheral Vascular Disease | yesno, Required<br>1 Yes<br>0 No |
| 22 | base_copd_asthma | Previous Chronic obstructive pulmonary disease (COPD) or Asthma | yesno, Required<br>1 Yes<br>0 No |
| 23 | base_pneumonia | Previous history of pneumonia<br>*Any type* | yesno, Required<br>1 Yes<br>0 No |
| 24 | baseline_hf | Previous Heart Failure | yesno, Required<br>1 Yes<br>0 No |
| 25 | base_hf_nhya<br>Show the field ONLY if:<br>[baseline_hf] = '1' | If the patient has previous heart failure, specify the New York Heart Association (NYHA) classification.<br>*https://es.wikipedia.org/wiki/Clasificación_funcional_New_York_Heart_Association* | radio, Required<br>1 I<br>2 II<br>3 III<br>4 IV |
| 26 | base_pul_hyper | Pulmonary hypertension<br>*Define by clinical history or PA systolic pressure >30 mmHg by echocardiogram.* | yesno, Required<br>1 Yes<br>0 No |
| 27 | base_vte_pe | Previous Venous Thromboembolism/Pulmonary Embolism | yesno, Required<br>1 Yes<br>0 No |
| 28 | base_bleeding | Previous Major bleeding<br>*Any history of bleeding requiring medical attention (i.e. gastrointestinal bleeding, intracranial bleeding, or hemorrhagic stroke, etc.)* | yesno, Required<br>1 Yes<br>0 No |
| 29 | base_dementia | Any dementia or cognitive impairment? | radio, Required<br>1 No<br>2 Any cognitive impairment<br>3 Any dementia |

| | | | |
|---|---|---|---|
| 30 | base_cancer | Previous Cancer | radio, Required<br>0 No<br>1 Previous<br>2 Active no treatment<br>3 Active under treatment |
| 31 | base_cancer_type<br>Show the field ONLY if:<br>[base_cancer] = '1' or [base_cancer] = '2' or [base_cancer] = '3' | If yes, specify the cancer type | text, Required |
| 32 | base_transplantation | The patient has any type of organ transplant? | yesno, Required<br>1 Yes<br>0 No |
| 33 | base_transplantation_type<br>Show the field ONLY if:<br>[base_transplantation] = '1' | Which type of organ transplant? | dropdown, Required<br>1 Renal<br>2 Liver<br>3 Heart<br>4 Bone narrow<br>5 Lung<br>6 Pancreatic<br>7 Intestinal<br>8 Renal and pancreatic<br>9 Lung and Heart<br>10 Liver and Heart<br>11 Renal and liver<br>12 Liver, heart and pancreas |
| 34 | other_disease_base | If any other relevant disease (i.e. immunosuppression)<br>*Use a comma to separate the diagnoses. Example: lupus, gastrinoma.* | notes |
| 35 | base_aspirin | Section Header: *BASELINE - TREATMENT*<br>Aspirin | yesno, Required<br>1 Yes<br>0 No |
| 36 | base_p2y12 | P2Y12 inhibitor<br>*Example: clopidogrel, prasugrel, Ticagrelor* | yesno, Required<br>1 Yes<br>0 No |
| 37 | base_p2y12_type<br>Show the field ONLY if:<br>[base_p2y12] ='1' | P2Y12 inhibitor | radio, Required<br>1 Clopidogrel<br>2 Prasugrel<br>3 Ticagrelor |
| 38 | base_ace_inh | ACE inhibitor<br>*Angiotensin-converting-enzyme inhibitors (ACE inhibitors)* | yesno, Required<br>1 Yes<br>0 No |
| 39 | base_ace_type<br>Show the field ONLY if:<br>[base_ace_inh] = '1' | ACE inhibitor type | radio, Required<br>1 Lisinopril<br>2 Enalapril<br>3 Perindopril<br>4 Other |
| 40 | base_ace_other<br>Show the field ONLY if:<br>[base_ace_type] = '4' | If other ACE inhibitor, specify which | text, Required |

| | | | |
|---|---|---|---|
| 41 | base_ace_dose<br><br>Show the field ONLY if:<br>[base_ace_inh] = '1' | ACE inhibitor TOTAL daily dose (mg)<br>*If unknown use 999.* | text (number, Min: 1, Max: 999) |
| 42 | base_arbs | ARBs<br>*Angiotensin II receptor blockers* | yesno, Required<br>1 Yes<br>0 No |
| 43 | base_arbs_type<br><br>Show the field ONLY if:<br>[base_arbs] = '1' | ARBs type | radio, Required<br>1 Losartan<br>2 Candesartan<br>3 Irbesartan<br>4 Telmisartan<br>5 Valsartan<br>6 Olmesartan<br>7 Other |
| 44 | base_arbs_other<br><br>Show the field ONLY if:<br>[base_arbs_type] = '7' | If other ARBs, specify which | text |
| 45 | base_abrs_dose<br><br>Show the field ONLY if:<br>[base_arbs] = '1' | ARBs TOTAL daily dose (mg)<br>*If unknown use 999.* | text (number, Min: 0, Max: 999), Required |
| 46 | base_arni | Angiotensin receptor neprilysin inhibitor (ARNI)<br>*Sacubitril/valsartan (Entresto)* | yesno, Required<br>1 Yes<br>0 No |
| 47 | base_arni_dose<br><br>Show the field ONLY if:<br>[base_arni] = '1' | Angiotensin receptor neprilysin inhibitor (ARNI) TOTAL daily dose (mg)<br>*Sacubitril/valsartan (Entresto). If unknown use 999.* | text (number, Min: 0, Max: 999) |
| 48 | base_statins | Statins | yesno, Required<br>1 Yes<br>0 No |
| 49 | base_statin_type<br><br>Show the field ONLY if:<br>[base_statins] = '1' | Statin type | radio, Required<br>1 Atorvastatin<br>2 Rosuvastatin<br>3 Simvastatin<br>4 Other |
| 50 | base_statin_other<br><br>Show the field ONLY if:<br>[base_statin_type] = '4' | If other statin, specify which | text, Required |
| 51 | base_statin_dose<br><br>Show the field ONLY if:<br>[base_statins] = '1' | Statin TOTAL daily dose (mg)<br>*If unknown use 999.* | text (number, Min: 0, Max: 999) |
| 52 | base_beta_block | Beta Blocker | yesno, Required<br>1 Yes<br>0 No |
| 53 | base_beta_block_type<br><br>Show the field ONLY if:<br>[base_beta_block] = '1' | Beta Blocker type | radio, Required<br>1 Bisoprolol<br>2 Atenolol<br>3 Metoprolol<br>4 Carvedilol<br>5 Other |

| | | | |
|---|---|---|---|
| 54 | base_beta_block_other<br><br>Show the field ONLY if:<br>[base_beta_block_type] = '5' | If other Beta blocker, specify which | text, Required |
| 55 | base_beta_block_dose<br><br>Show the field ONLY if:<br>[base_beta_block] = '1' | Beta Blocker TOTAL daily dose (mg)<br>*If unknown use 999.* | text (number, Min: 0, Max: 999) |
| 56 | base_ccb | Calcium channel blocker | yesno, Required<br>1 Yes<br>0 No |
| 57 | base_ccb_type<br><br>Show the field ONLY if:<br>[base_ccb] = '1' | Calcium channel blockers type | radio, Required<br>1 Amlodipine<br>2 Diltiazem<br>3 Nicardipine<br>4 Nifedipine<br>5 Verapamil<br>6 Other |
| 58 | base_ccb_other<br><br>Show the field ONLY if:<br>[base_ccb_type] = '6' | If other calcium channel blocker, specify which | text, Required |
| 59 | base_ccb_dose<br><br>Show the field ONLY if:<br>[base_ccb] = '1' | Calcium channel blocker TOTAL daily dose (mg)<br>*If unknown use 999.* | text (number, Min: 0, Max: 999), Required |
| 60 | base_loop_diuretics | Loop diuretic<br>*Example: Furosemide, Torsemide, etc.* | yesno, Required<br>1 Yes<br>0 No |
| 61 | base_loop_diuretic_dose<br><br>Show the field ONLY if:<br>[base_loop_diuretics] = '1' | Loop diuretic TOTAL daily dose (mg)<br>*If unknown use 999.* | text (number, Min: 0, Max: 999), Required |
| 62 | base_mras | Mineralocorticoid receptor antagonists (MRAs)<br>*Spironolactone or eplerenone* | yesno, Required<br>1 Yes<br>0 No |
| 63 | base_mras_type<br><br>Show the field ONLY if:<br>[base_mras] ='1' | Mineralocorticoid receptor antagonists type | radio, Required<br>1 Spironolactone<br>2 Eplerenone |
| 64 | base_mras_dose<br><br>Show the field ONLY if:<br>[base_mras] ='1' | Mineralocorticoid receptor antagonists (MRAs) TOTAL daily dose (mg)<br>*If unknown use 999.* | text (number, Min: 0, Max: 999), Required |
| 65 | base_ppis | Proton-pump inhibitors | yesno, Required<br>1 Yes<br>0 No |
| 66 | base_oral_hypo_glc | Oral hypoglycemic agents | yesno, Required<br>1 Yes<br>0 No |
| 67 | base_insulin | Insulin | yesno, Required<br>1 Yes<br>0 No |
| 68 | base_anticoagulant | Anticoagulant treatment<br>*Example: sintrom, ACODs, enoxaparin, etc.* | yesno, Required<br>1 Yes<br>0 No |

| | | | |
|---|---|---|---|
| 69 | base_anticoagulant_tx<br><br>Show the field ONLY if:<br>[base_anticoagulant] = '1' | Which anticoagulant treatment<br>**Example: Rivaroxaban, dabigatran, apixaban, or edoxaban.* | radio, Required<br>1 Vitamin K antagonists (acenocoumarol)<br>2 Directly acting oral anticoagulants*<br>3 Low-molecular-weight heparin (enoxaparin, etc.) |
| 70 | base_noac_type<br><br>Show the field ONLY if:<br>[base_anticoagulant_tx] = '2' | If Directly acting oral anticoagulants was used which one: | radio, Required<br>1 Rivaroxaban (Xarelto)<br>2 Dabigatran (Pradaxa)<br>3 Apixaban (Eliquis)<br>4 Edoxaban (Lixiana) |
| 71 | base_noac_dose<br><br>Show the field ONLY if:<br>[base_anticoagulant_tx] = '2' | Specify the Directly acting oral anticoagulants TOTAL Daily dose (mg)<br>*If unknown use 999.* | text (number, Min: 0, Max: 999), Required |
| 72 | base_nsaids | Nonsteroidal anti-inflammatory drugs (NSAIDs) | radio, Required<br>0 No<br>1 Yes - Chronic use<br>2 Yes - Only recent use |
| 73 | base_nsaids_type<br><br>Show the field ONLY if:<br>[base_nsaids] = '1' or [base_nsaids] = '2' | NSAIDs type | radio<br>1 Ibuprofen<br>2 Diclofenac<br>3 Naproxen<br>4 Dexketoprofen or ketoprofen<br>5 Celecoxib<br>6 Other |
| 74 | base_nsaids_other<br><br>Show the field ONLY if:<br>[base_nsaids_type] = '6' | If other Nonsteroidal anti-inflammatory drugs (NSAIDs), specify which | text, Required |
| 75 | base_nsaids_dose<br><br>Show the field ONLY if:<br>[base_nsaids] = '1' or [base_nsaids] = '2' | NSAIDs total daily dose (mg)<br>*If unknown use 9999.* | text (number, Min: 0, Max: 9999), Required |
| 76 | base_paracetamol | Paracetamol | yesno, Required<br>1 Yes<br>0 No |
| 77 | base_other_tx | If any other relevant medication (i.e. Immunosuppressive therapy,) specify which<br>*Use a comma to separate the diagnoses. Example: ezetimibe, levothyroxine.* | notes |
| 78 | base_lvef | Left ventricular ejection fraction (LVEF) (%)<br>Assessed by any method.<br>*If unknow use 999.* | text (number, Min: 0, Max: 999), Required |
| 79 | base_vhd_mod_sev | Did the patient have any Moderate or Severe valvular heart disease?<br>*Example: aortic stenosis, aortic regurgitation, Mitral regurgitation, Tricuspid regurgitation, etc.* | yesno, Required<br>1 Yes<br>0 No |
| 80 | base_vhd_mod_sev_1<br><br>Show the field ONLY if:<br>[base_vhd_mod_sev] = '1' | Specify which type of Moderate or severe valvular heart disease | radio, Required<br>1 Aortic stenosis<br>2 Aortic regurgitation<br>3 Mitral regurgitation<br>4 Mitral Stenosis<br>5 Tricuspid regurgitation |

| | | | |
|---|---|---|---|
| 81 | base_vhd_mod_sev_2<br><br>Show the field ONLY if:<br>[base_vhd_mod_sev] = '1' | If the patient had a second Moderate or severe valvular heart disease, specify which type | radio<br>1 Aortic stenosis<br>2 Aortic regurgitation<br>3 Mitral regurgitation<br>4 Mitral Stenosis<br>5 Tricuspid regurgitation |
| 82 | base_valve_replacement | Did the patient have any type of previous valve repair or replacement?<br>*Surgical or transcatheter valve replacement.* | yesno, Required<br>1 Yes<br>0 No |
| 83 | base_valve_replace_type<br><br>Show the field ONLY if:<br>[base_valve_replacement] = '1' | Which type of valve repair or replacement? | radio, Required<br>1 Transcatheter valve replacement<br>2 Surgical valve replacement<br>3 Surgical valve repair<br>4 Transcatheter and Surgical valve replacement |
| 84 | base_valve_replaced<br><br>Show the field ONLY if:<br>[base_valve_replacement] = '1' | Which cardiac valve was repair or replaced? | radio, Required<br>1 Aortic<br>2 Mitral<br>3 Tricuspid<br>4 Aortic and Mitral<br>5 Aortic, Mitral, and Tricuspid<br>6 Other |
| 85 | base_valve_replaced_other<br><br>Show the field ONLY if:<br>[base_valve_replaced] = '6' | If other cardiac valve was replaced, specify which | text, Required |
| 86 | base_cci | Section Header: *BASELINE - COMORBIDITY AND FRAILTY*<br>Calculate the Charlson Comorbidity Index (CCI) and provide the numerical value (minimum 0 maximum 37) | text (number, Min: 0, Max: 50), Required |
| 87 | base_cci_weblink | Online Charlson Comorbidity index calculator (https://www.mdcalc.com/charlson-comorbidity-index-cci) | descriptive |
| 88 | base_frailty | Patient functional health status | radio, Required<br>0 Independent<br>1 Partially dependent<br>2 Totally dependent |
| 89 | baseline_complete | Section Header: *Form Status*<br>Complete? | dropdown<br>0 Incomplete<br>1 Unverified<br>2 Complete |

Instrument: **COVID-19 status & Symptoms** (covid19_status_symptoms) Collapse

| | | | |
|---|---|---|---|
| *90* | covid_swab | Section Header: *COVID-19 STATUS*<br>Coronavirus swab result | radio, Required<br>0 Negative<br>1 Positive |
| 91 | covid_swab_date | Coronavirus swab date<br>*Date in which the coronavirus swab was performed.* | text (date_dmy, Min: 2020-02-01), Required |
| 92 | covid_other_virus<br><br>Show the field ONLY if:<br>[covid_swab] = 0 | If swab negative for coronavirus, specify if there was any other respiratory virus. | radio, Required<br>0 No<br>1 Influenza A<br>2 Influenza B<br>3 Human respiratory syncytial virus |

| | | | |
|---|---|---|---|
| 93 | hospitalization | Section Header: *NEED OF HOSPITAL ADMISSION*<br>Did the patient require hospital admission?<br>*In any moment.* | radio, Required<br>0 No - (Ambulatory care in subjects home)<br>1 Yes - (Emergency department, hospital admission, health hotel, etc.) |
| 94 | covid_antibody_test | Section Header: *COVID-19 ANTIBODY TEST*<br>Did the patient perform a Coronavirus antibody test?<br>*Serology test, any type.* | yesno, Required<br>1 Yes<br>0 No |
| 95 | covid_antibody_date<br>Show the field ONLY if:<br>[covid_antibody_test] = '1' | If the patient performed a Coronavirus antibody test, indicate the date. | text (date_dmy, Min: 2020-02-01), Required |
| 96 | covid_antibody_igg<br>Show the field ONLY if:<br>[covid_antibody_test] = '1' | If the patient performed an antibody test, provide the result of IgG. | radio<br>0 Negative<br>1 Positive |
| 97 | covid_antibody_igm<br>Show the field ONLY if:<br>[covid_antibody_test] = '1' | If the patient performed an antibody test, provide the result of IgM. | radio<br>0 Negative<br>1 Positive |
| 98 | covid_antibody_iga<br>Show the field ONLY if:<br>[covid_antibody_test] = '1' | If the patient performed an antibody test, provide the result of IgA. | radio<br>0 Negative<br>1 Positive |
| 99 | covid_symptons | Section Header: *PATIENT SYMPTOMS*<br>Did the patient present any suspected viral disease symptoms?<br>*Example: fever, dyspnea, cough, diarrhea, anosmia/ageusia, sore throat, cephalea, etc. Choose NO only when the patient was completely asymptomatic.* | yesno, Required<br>1 Yes<br>0 No |
| 100 | covid_symptons_date<br>Show the field ONLY if:<br>[covid_symptons] = '1' | Beginning of symptoms | text (date_dmy, Min: 2020-01-01), Required |
| 101 | symp_fever<br>Show the field ONLY if:<br>[covid_symptons] = '1' | Fever (>37.3ºC) | yesno, Required<br>1 Yes<br>0 No |
| 102 | symp_dysnea<br>Show the field ONLY if:<br>[covid_symptons] = '1' | Dyspnea | yesno, Required<br>1 Yes<br>0 No |
| 103 | symp_cough<br>Show the field ONLY if:<br>[covid_symptons] = '1' | Cough | radio, Required<br>1 No<br>2 Yes without sputum<br>3 Yes with sputum |
| 104 | symp_diarrhea<br>Show the field ONLY if:<br>[covid_symptons] = '1' | Diarrhea | yesno, Required<br>1 Yes<br>0 No |
| 105 | symp_anosmia<br>Show the field ONLY if:<br>[covid_symptons] = '1' | Anosmia or Ageusia | yesno, Required<br>1 Yes<br>0 No |
| 106 | covid19_status_symptoms_complete | Section Header: *Form Status*<br>Complete? | dropdown<br>0 Incomplete<br>1 Unverified<br>2 Complete |

Instrument: **Hospitalization** (hospitalization) Collapse

| | | | |
|---|---|---|---|
| 107 | inhosp_adm_date<br><br>Show the field ONLY if:<br>[hospitalization] = '1' | Section Header: *HOSPITALIZATION (including (Emergency department, hospital admission, and health hotel).*<br><br>Hospital admission date | text (date_dmy, Min: 2020-02-01), Required |
| 108 | inhosp_covid_admission<br><br>Show the field ONLY if:<br>[hospitalization] = '1' | Was hospital admission related to COVID-19?<br>*Including emergency department, hospital admission, health hotel, etc.* | yesno, Required<br>1 Yes<br>0 No |
| 109 | inhosp_cv_disease<br><br>Show the field ONLY if:<br>[inhosp_covid_admission] = '0' | Was the patient admitted to the hospital because of cardiovascular disease? | yesno, Required<br>1 Yes<br>0 No |
| 110 | inhosp_cv_diag<br><br>Show the field ONLY if:<br>[inhosp_cv_disease] = '1' | Specify which cardiovascular disease (including pulmonary embolism)? | dropdown, Required<br>1 STEMI<br>2 NSTEMI<br>3 Chest pain/angor (negative troponin)<br>4 Heart failure<br>5 Cardiogenic Shock<br>6 Supraventricular Arrhythmia<br>7 Ventricular Arrhythmia<br>8 Syncope<br>9 Cardiac arrest<br>10 Pulmonary embolism<br>11 Aortic dissection<br>12 Endocarditis<br>13 Other |
| 111 | inhosp_cv_diag_other<br><br>Show the field ONLY if:<br>[inhosp_cv_diag] = '13' | If other, specify which cardiovascular disease. | text, Required |
| 112 | inhosp_noncv_adm_specify<br><br>Show the field ONLY if:<br>[inhosp_cv_disease] = '0' | If the patient was the patient admitted with a non-cardiovascular diagnosis, specify which (DIAGNOSIS, SPECIALTY) and if any SURGERY was needed?<br>*Use a comma to separate. Example: hip fracture, trauma, hip replacement.* | notes |
| 113 | inhosp_icu_semi<br><br>Show the field ONLY if:<br>[hospitalization] = '1' | Did the patient need intensive or semi-intensive care unit admission? | yesno, Required<br>1 Yes<br>0 No |
| 114 | inhosp_icu_semi_admin<br><br>Show the field ONLY if:<br>[inhosp_icu_semi] = '1' | If intensive or semi-intensive was needed, specify where.<br>*If the patient was admitted in both, choose the Intensive care Unit.* | radio, Required<br>1 Semi-Intensive Care Units<br>2 Intensive care Unit |
| 115 | inhosp_icu_adm_date<br><br>Show the field ONLY if:<br>[inhosp_icu_semi] = '1' | Intensive Care Unit admission date | text (date_dmy, Min: 2020-02-01), Required |
| 116 | inhosp_icu_disch_date<br><br>Show the field ONLY if:<br>[inhosp_icu_semi] = '1' | Intensive care Unit discharge date | text (date_dmy, Min: 2020-02-01), Required |
| 117 | inhosp_imv<br><br>Show the field ONLY if:<br>[inhosp_icu_semi] = '1' | Invasive mechanical ventilation | yesno, Required<br>1 Yes<br>0 No |
| 118 | inhosp_ecmo<br><br>Show the field ONLY if:<br>[inhosp_icu_semi] = '1' | Extracorporeal membrane oxygenation (ECMO) | radio, Required<br>0 No<br>1 ECMO V-V<br>2 ECMO V-A |

| | | | |
|---|---|---|---|
| 119 | inhosp_rrt<br><br>Show the field ONLY if:<br>[hospitalization] = '1' | Renal replacement therapy | yesno, Required<br>1 Yes<br>0 No |
| 120 | inhosp_vasodrug<br><br>Show the field ONLY if:<br>[hospitalization] = '1' | Need of vasoactive agents<br>*Example: dopamine, dobutamine, noradrenaline* | yesno, Required<br>1 Yes<br>0 No |
| 121 | inhosp_lvef<br><br>Show the field ONLY if:<br>[hospitalization] = '1' | Left-ventricular ejection fraction (%)<br>*Assessed by any method. If unknown use 999.* | text (number, Min: 0, Max: 999), Required |
| 122 | inhosp_cath_available<br><br>Show the field ONLY if:<br>[hospitalization] = '1' | Section Header: *CARDIAC CATHETERIZATION FINDINGS*<br>Was a cardiac catheterization performed?<br>*PCI: percutaneous cardiac intervention* | yesno, Required<br>1 Yes<br>0 No |
| 123 | inhosp_cath_pain_date<br><br>Show the field ONLY if:<br>[inhosp_cath_available] = '1' | Date of the cardiac catheterization | text (date_dmy, Min: 2020-02-01), Required |
| 124 | inhosp_cath_indication<br><br>Show the field ONLY if:<br>[inhosp_cath_available] = '1' | Cardiac catheterization clinical indication | radio, Required<br>1 Angina pectoris (troponin negative)<br>2 Unstable angina (troponin negative)<br>3 NSTEMI<br>4 STEMI |
| 125 | inhosp_cath_culprit<br><br>Show the field ONLY if:<br>[inhosp_cath_available] = '1' | Was there a culprit lesion? | radio, Required<br>0 No<br>1 Yes - No thrombotic<br>2 Yes - Thrombotic |
| 126 | inhosp_cath_coro_seg<br><br>Show the field ONLY if:<br>[inhosp_cath_available] = '1' | Choose the culprit lesion coronary segment | radio<br>1 Left main coronary artery<br>2 Left anterior descending artery<br>3 Left circumflex artery<br>4 Right coronary artery |
| 127 | inhosp_cath_mv<br><br>Show the field ONLY if:<br>[inhosp_cath_available] = '1' | Multi-vessel disease | yesno, Required<br>1 Yes<br>0 No |
| 128 | inhosp_cath_coro_seg_non1<br><br>Show the field ONLY if:<br>[inhosp_cath_mv] = '1' | If multi-vessel disease, choose the non-culprit lesion #1 coronary segment | radio<br>1 Left main coronary artery<br>2 Left anterior descending artery<br>3 Left circumflex artery<br>4 Right coronary artery |
| 129 | inhosp_cath_coro_seg_non2<br><br>Show the field ONLY if:<br>[inhosp_cath_mv] = '1' | If multi-vessel disease, choose the non-culprit lesion #2 coronary segment | radio<br>1 Left main coronary artery<br>2 Left anterior descending artery<br>3 Left circumflex artery<br>4 Right coronary artery |
| 130 | inhosp_pci<br><br>Show the field ONLY if:<br>[hospitalization] = '1' | Was Percutaneous coronary intervention (PCI) performed?<br>*Example: thrombus aspiration, stent implantation, drug-eluting balloon.* | yesno, Required<br>1 Yes<br>0 No |
| 131 | inhosp_cath_asa_ld<br><br>Show the field ONLY if:<br>[inhosp_pci] = '1' | Aspirin loading dose? | yesno, Required<br>1 Yes<br>0 No |

| | | | |
|---|---|---|---|
| 132 | inhosp_cath_p2y12_ld<br><br>Show the field ONLY if:<br>[inhosp_pci] = '1' | P2Y12 inhibitor loading dose?<br>*If the patient was loaded with clopidogrel and thereafter was switch to Prasugrel or Ticagrelor, select the latter.* | radio, Required<br>0 No<br>1 Clopidogrel<br>2 Prasugrel<br>3 Ticagrelor |
| 133 | inhosp_cath_anticoag<br><br>Show the field ONLY if:<br>[inhosp_pci] = '1' | Anticoagulation treatment during the procedure? | radio, Required<br>0 No<br>1 Unfractioned heparin<br>2 Bivalirudin |
| 134 | inhosp_pci_gpi<br><br>Show the field ONLY if:<br>[inhosp_pci] = '1' | Was GPI used (IIB/IIIA inhibitors)? | yesno, Required<br>1 Yes<br>0 No |
| 135 | inhosp_cath_device<br><br>Show the field ONLY if:<br>[inhosp_pci] = '1' | Device type | radio, Required<br>0 Trombus aspiration only<br>1 Bare-metal stent<br>2 Drug-eluting stent<br>3 Drug-eluting balloon |
| 136 | inhosp_cath_device_brand<br><br>Show the field ONLY if:<br>[inhosp_cath_device] = '1' or [inhosp_cath_device] = '2' or [inhosp_cath_device] = '3' | Please provide the device commercial name<br>*Example: xience, onyx, orsiro, etc.* | text, Required |
| 137 | inhosp_pci_compli<br><br>Show the field ONLY if:<br>[inhosp_cath_available] = '1' | Describe any complication during the procedure:<br>1.- Site access hematoma/bleeding<br>2.- Coronary dissection/perforation<br>3.- Acute Stent thrombosis<br>*Use a comma to separate the complications.* | notes |
| 138 | inhosp_cath_ventri<br><br>Show the field ONLY if:<br>[inhosp_cath_available] = '1' | Was left ventriculography performed? | yesno, Required<br>1 Yes<br>0 No |
| 139 | inhosp_cath_ventri_tako<br><br>Show the field ONLY if:<br>[inhosp_cath_ventri] = '1' | If ventriculography performed, was there any Takotsubo pattern?<br>*Any pattern (Apical, mid ventricular, basal or focal) https://takotsubo.net/information/about-takotsubo-syndrome/* | yesno, Required<br>1 Yes<br>0 No |
| 140 | inhosp_cath_second<br><br>Show the field ONLY if:<br>[inhosp_cath_available] = '1' | If the patient had a Second cardiac catheterization (for example, stent thrombosis or revascularization), specify the following:<br>Date, diagnosis, procedure, and outcome:<br>*Use a comma to separate the date, diagnosis, procedure, and outcome.* | notes |
| 141 | inhosp_thrombo<br><br>Show the field ONLY if:<br>[hospitalization] = '1' | Section Header: *IN-HOSPITAL THROMBOLYSIS THERAPY*<br>Was thrombolysis therapy performed? | yesno, Required<br>1 Yes<br>0 No |
| 142 | inhosp_thrombo_why<br><br>Show the field ONLY if:<br>[inhosp_thrombo] = '1' | If thrombolysis was performed, which was the clinical indication? | radio, Required<br>1 ST-segment elevation Myocardial infarction<br>2 Ischemic stroke<br>3 Pulmonary embolism |
| 143 | inhosp_anticoag<br><br>Show the field ONLY if:<br>[hospitalization] = '1' | Section Header: *IN-HOSPITAL ANTICOAGULANT TREATMENT*<br>Was the patient treated with any anticoagulant therapy?<br>*Example: acenocoumarol, enoxaparin, rivaroxaban, unfractionated heparin, etc.* | yesno, Required<br>1 Yes<br>0 No |

| | | | |
|---|---|---|---|
| 144 | inhosp_anticoag_type<br>Show the field ONLY if: [inhosp_anticoag] = '1' | Which type of Anticoagulant treatment?<br>**Example: Rivaroxaban, dabigatran, apixaban, or edoxaban.* | radio, Required<br>1 Vitamin K antagonists (acenocoumarol)<br>2 Directly acting oral anticoagulants*<br>3 Low-molecular-weight heparin (Enoxaparin, etc.)<br>4 Unfractionated heparin (UFH) (Sodic) |
| 145 | inhosp_anticoag_type_2nd<br>Show the field ONLY if: [inhosp_anticoag] = '1' | If the patient was treated with a second anticoagulant treatment, specify which<br>**Example: Rivaroxaban, dabigatran, apixaban, or edoxaban.* | radio<br>1 Vitamin K antagonists (acenocoumarol)<br>2 Directly acting oral anticoagulants*<br>3 Low-molecular-weight heparin (Enoxaparin, etc.)<br>4 Unfractionated heparin (UFH) (Sodic) |
| 146 | inhosp_anticoagulant_why<br>Show the field ONLY if: [inhosp_anticoag] = '1' | Clinical indication for anticoagulant treatment | radio, Required<br>1 Prophylaxis<br>2 Atrial fibrillation<br>3 Acute coronary syndrome<br>4 Venous thromboembolism<br>5 Pulmonary embolism<br>6 Arterial embolism<br>7 Stroke<br>8 Cardiac intraventricular thrombus<br>9 Other |
| 147 | inhosp_anticoag_other<br>Show the field ONLY if: [inhosp_anticoagulant_why] = '9' | If other indication for anticoagulant treatment, specify which | text, Required |
| 148 | inhosp_anticoagulant_why_2<br>Show the field ONLY if: [inhosp_anticoag] = '1' | If there was a second clinical indication for anticoagulant treatment, specify which | radio<br>1 Prophylaxis<br>2 Atrial fibrillation<br>3 Acute coronary syndrome<br>4 Venous thromboembolism<br>5 Pulmonary embolism<br>6 Arterial embolism<br>7 Stroke<br>8 Cardiac intraventricular thrombus |
| 149 | inhosp_lwmh_tx_type<br>Show the field ONLY if: [inhosp_anticoag_type] = 3 or [inhosp_anticoag_type_2nd] = 3 | Which Low-molecular-weight heparin was used? | radio, Required<br>1 Enoxaparin<br>2 Dalteparin<br>3 Bemiparin<br>4 Tinzaparin<br>5 Fondaparinux<br>6 Nadroparin<br>7 Certoparin<br>8 Other |
| 150 | inhosp_lmwh_other<br>Show the field ONLY if: [inhosp_lwmh_tx_type] = '8' | If other, please specify the type of Low-molecular-weight heparin: | text, Required |
| 151 | inhosp_lmwh_dose<br>Show the field ONLY if: [inhosp_anticoag_type] = 3 or [inhosp_anticoag_type_2nd] = 3 | Specify the highest dose of Low-molecular-weight heparin (only numbers) that was used.<br>*Example: 40 mg or 4000 UI (only numbers).* | text (number, Min: 0, Max: 10000000), Required |

| | | | |
|---|---|---|---|
| 152 | inhosp_lmwh_unit<br><br>Show the field ONLY if:<br>[inhosp_anticoag_type] = 3 or [inhosp_anticoag_type_2nd] = 3 | Which was the Low-molecular-weight heparin UNIT? (mg or UI)<br>*mg or UI* | radio, Required<br>1 mg<br>2 UI |
| 153 | inhosp_lmwh_frequency<br><br>Show the field ONLY if:<br>[inhosp_anticoag_type] = 3 or [inhosp_anticoag_type_2nd] = 3 | Which was the frequency of administration of the Low-molecular-weight heparin? | radio, Required<br>1 Each 24 hours<br>2 Each 12 hours |
| 154 | inhosp_high_dose_date<br><br>Show the field ONLY if:<br>[coordinating_center] = '1' and [inhosp_anticoag_type] = 3 or [inhosp_anticoag_type_2nd] = 3 | What day was the highest dose of low-weight molecular heparin started? | text (date_dmy, Min: 2020-02-01), Required |
| 155 | inhosp_lmwh_days<br><br>Show the field ONLY if:<br>[coordinating_center] = '1' and [inhosp_anticoag_type] = 3 or [inhosp_anticoag_type_2nd] = 3 | TOTAL days of low-weight molecular heparin treatment | text (number, Min: 0, Max: 100), Required |
| 156 | inhosp_noac_type<br><br>Show the field ONLY if:<br>[inhosp_anticoag_type] = 2 or [inhosp_anticoag_type_2nd] = 2 | If Directly acting oral anticoagulants were used, specify which | radio, Required<br>1 Rivaroxaban (Xarelto)<br>2 Dabigatran (Pradaxa)<br>3 Apixaban (Eliquis)<br>4 Edoxaban (Lixiana) |
| 157 | inhosp_noac_dose<br><br>Show the field ONLY if:<br>[inhosp_anticoag_type] = 2 or [inhosp_anticoag_type_2nd] = 2 | Specify the Directly acting oral anticoagulants TOTAL Daily dose (mg)<br>*If unknown use 999.* | text (number, Min: 0, Max: 999), Required |
| 158 | inhosp_aspirin<br><br>Show the field ONLY if:<br>[hospitalization] = '1' | Section Header: *IN-HOSPITAL TREATMENT (If data is not available, select not available option)*<br>Aspirin | radio, Required<br>0 No<br>1 Yes<br>2 Not available |
| 159 | inhosp_p2y12<br><br>Show the field ONLY if:<br>[hospitalization] = '1' | P2Y12 inhibitor<br>*Example: clopidogrel, prasugrel, Ticagrelor* | radio, Required<br>0 No<br>1 Yes<br>2 Not available |
| 160 | inhosp_p2y12_type<br><br>Show the field ONLY if:<br>[inhosp_p2y12] = '1' | Which P2Y12 inhibitor | radio, Required<br>1 Clopidogrel<br>2 Prasugrel<br>3 Ticagrelor<br>4 Not available |
| 161 | inhosp_ace_inh<br><br>Show the field ONLY if:<br>[hospitalization] = '1' | ACE inhibitor<br>*Angiotensin-converting-enzyme inhibitors (ACE inhibitors)* | radio, Required<br>0 No<br>1 Yes<br>2 Not available |
| 162 | inhosp_arbs<br><br>Show the field ONLY if:<br>[hospitalization] = '1' | ARBs<br>*Angiotensin II receptor blockers* | radio, Required<br>0 No<br>1 Yes<br>2 Not available |

| | | | |
|---|---|---|---|
| 163 | inhosp_statins<br><br>Show the field ONLY if:<br>[hospitalization] = '1' | Statins | radio, Required<br>0 No<br>1 Yes<br>2 Not available |
| 164 | inhosp_beta_block<br><br>Show the field ONLY if:<br>[hospitalization] = '1' | Beta Blocker | radio, Required<br>0 No<br>1 Yes<br>2 Not available |
| 165 | inhosp_ccb<br><br>Show the field ONLY if:<br>[hospitalization] = '1' | Calcium channel blocker | radio, Required<br>0 No<br>1 Yes<br>2 Not available |
| 166 | inhosp_loop_diuretics<br><br>Show the field ONLY if:<br>[hospitalization] = '1' | Loop diuretic<br>*Example: Furosemide, Torsemide, etc.* | radio, Required<br>0 No<br>1 Yes<br>2 Not available |
| 167 | inhosp_mras<br><br>Show the field ONLY if:<br>[hospitalization] = '1' | Mineralocorticoid receptor antagonists (MRAs)<br>*Spironolactone or eplerenone* | radio, Required<br>0 No<br>1 Yes<br>2 Not available |
| 168 | inhosp_thrombofilia_study<br><br>Show the field ONLY if:<br>[coordinating_center] = '1' and [hospitalization] = '1' | Section Header: *IN-HOSPITAL - THROMBOPHILIA AND/OR AUTOINMMUNITY STUDY*<br>Was thrombophilia study performed to the patient? | yesno, Required<br>1 Yes<br>0 No |
| 169 | inhosp_thrombofilia_lab<br><br>Show the field ONLY if:<br>[inhosp_thrombofilia_study] = '1' | If a thrombophilia study was done, indicate the date of the laboratory. | text (date_dmy, Min: 2020-02-01), Required |
| 170 | inhosp_autoimmunity_study<br><br>Show the field ONLY if:<br>[coordinating_center] = '1' and [hospitalization] = '1' | Was autoimmunity study performed to the patient? | yesno, Required<br>1 Yes<br>0 No |
| 171 | inhosp_autoinmune_lab<br><br>Show the field ONLY if:<br>[inhosp_autoimmunity_study] = '1' | If a autoinmmunity study was done, indicate the date of the laboratory.<br>*Please anonymize the report and upload it* | text (date_dmy, Min: 2020-02-01), Required |
| 172 | hospitalization_complete | Section Header: *Form Status*<br>Complete? | dropdown<br>0 Incomplete<br>1 Unverified<br>2 Complete |

Instrument: **COVID-19 treatment** (covid19_treatment) Collapse

| | | | |
|---|---|---|---|
| 173 | covid_tx_kaletra | Section Header: *COVID-19 SPECIFIC TREATMENT (at any time or setting [ambulatory or hosptial])*<br>Kaletra (Lopinavir/Ritonavir)? | yesno, Required<br>1 Yes<br>0 No |
| 174 | covid_tx_hydroxyclor | Hydroxychloroquine? | yesno, Required<br>1 Yes<br>0 No |
| 175 | covid_tx_azithro | Azithromycin? | yesno, Required<br>1 Yes<br>0 No |

| | | | |
|---|---|---|---|
| 176 | covid_tx_toci | Tocilizumab (Actemra)? | yesno, Required<br>1 Yes<br>0 No |
| 177 | covid_tx_antibody | Did the patient was treated with a second monoclonal antibody? (Example: Canakinumab, etc.)<br>*Example: Canakinumab, Sarilumab, or Siltuximab.* | yesno, Required<br>1 Yes<br>0 No |
| 178 | covid_tx_antibody_type<br>Show the field ONLY if:<br>[covid_tx_antibody] = '1' | Which monoclonal antibody? | radio, Required<br>1 Canakinumab (Anakinra)<br>2 Sarilumab (Kevzara)<br>3 Siltuximab (Sylvant)<br>4 Other |
| 179 | covid_tx_antibody_other<br>Show the field ONLY if:<br>[covid_tx_antibody_type] = '4' | If other, specify which monoclonal antibody | text, Required |
| 180 | covid_tx_cortis | Corticosteroids<br>*Example: Methylprednisolone, prednisone, dexamethasone, etc.* | yesno, Required<br>1 Yes<br>0 No |
| 181 | covid_tx_cortis_type<br>Show the field ONLY if:<br>[covid_tx_cortis] = '1' | Specify which type of Corticosteroids | radio, Required<br>1 Methylprednisolone<br>2 Dexamethasone<br>3 Prednisone<br>4 Prednisolone<br>5 Hydrocortisone<br>6 Other |
| 182 | covid_tx_cortis_other<br>Show the field ONLY if:<br>[covid_tx_cortis_type] = '6' | If other corticosteroids, specify which: | text, Required |
| 183 | covid_tx_cortis_dose<br>Show the field ONLY if:<br>[covid_tx_cortis] = '1' | Which was the maximum used dose of corticosteroids (mg)?<br>*Example: 250 mg (only numbers).* | text, Required |
| 184 | covid_tx_remde | Remdesivir? | yesno, Required<br>1 Yes<br>0 No |
| 185 | inhosp_nsaids | Nonsteroidal anti-inflammatory drugs (NSAIDs) | yesno, Required<br>1 Yes<br>0 No |
| 186 | covid_nsaid_type<br>Show the field ONLY if:<br>[inhosp_nsaids] = '1' | If non steroidal anti inflammatory (NSAIDs) were use, specify which type | radio, Required<br>1 Ibuprofen<br>2 Diclofenac<br>3 Naproxen<br>4 Dexketoprofen or ketoprofen<br>5 Celecoxib<br>6 Other |
| 187 | inhosp_nsaids_other<br>Show the field ONLY if:<br>[covid_nsaid_type] = '6' | If other non steroidal anti inflammatory (NSAIDs), specify which | text, Required |
| 188 | covid_paracetamol | Paracetamol | yesno, Required<br>1 Yes<br>0 No |

| | | | |
|---|---|---|---|
| 189 | covid_tx_other | If there was any other COVID-19 specify treatment, specify:<br>*Use a comma to separate.* | notes |
| 190 | covid19_treatment_complete | Section Header: *Form Status*<br>Complete? | dropdown<br>0 Incomplete<br>1 Unverified<br>2 Complete |
| Instrument: **Biomarkers** (biomarkers) | | | Collapse |
| 191 | inhosp_ctni | Section Header: *INDEX EVALUATION - CARDIAC BIOMARKERS (In-hospital, emergency department, or ambulatory)*<br>Was the patient tested for Troponin I? | yesno, Required<br>1 Yes<br>0 No |
| 192 | inhosp_cnti_positive_adm<br>Show the field ONLY if:<br>[inhosp_ctni] = '1' | Was Troponin I already elevated in the first test? | yesno, Required<br>1 Yes<br>0 No |
| 193 | inhosp_ctni_peak<br>Show the field ONLY if:<br>[inhosp_ctni] = '1' | Hs-cardiac Troponin I peak<br>*Highest value (numerical) during the index hospitalization or ambulatory.* | text (number, Min: 0, Max: 10000000), Required |
| 194 | inhosp_ctni_unit<br>Show the field ONLY if:<br>[inhosp_ctni] = '1' | Hs-cTn-I - units | dropdown, Required<br>1 ng/L<br>2 ng/mL<br>3 µg/L<br>4 pg/mL<br>5 ng/dL |
| 195 | inhosp_ctni_peak_date<br>Show the field ONLY if:<br>[inhosp_ctni] = '1' | Hs-cTn-I peak date | text (date_dmy, Min: 2020-02-24), Required |
| 196 | inhosp_ctnt | Was the patient tested for Troponin T? | yesno, Required<br>1 Yes<br>0 No |
| 197 | inhosp_cntt_positive_adm<br>Show the field ONLY if:<br>[inhosp_ctnt] = '1' | Was Troponin T already elevated in the first test? | yesno, Required<br>1 Yes<br>0 No |
| 198 | inhosp_ctnt_peak<br>Show the field ONLY if:<br>[inhosp_ctnt] = '1' | Hs-cTn-T peak<br>*Highest value (numerical) during the index hospitalization or ambulatory.* | text (number, Min: 0, Max: 10000000), Required |
| 199 | inhosp_ctnt_unit<br>Show the field ONLY if:<br>[inhosp_ctnt] = '1' | Hs-cTn-T - units | dropdown, Required<br>1 ng/L<br>2 ng/mL<br>3 µg/L<br>4 pg/mL<br>5 ng/dL |
| 200 | inhosp_ctnt_peak_date<br>Show the field ONLY if:<br>[inhosp_ctnt] = '1' | Hs-cTn-T peak date | text (date_dmy, Min: 2020-02-24), Required |
| 201 | carbio_bnp | Was the patient tested for B-type natriuretic peptide (BNP) or NT-BNP? | yesno, Required<br>1 Yes<br>0 No |
| 202 | inhosp_pronbp_peak<br>Show the field ONLY if:<br>[carbio_bnp] = 1 | NT-proBNP peak (pg/mL)<br>*Highest value (numerical) during the index hospitalization or ambulatory.* | text (number, Min: 0, Max: 10000000), Required |

| | | | |
|---|---|---|---|
| 203 | inhosp_bnp_peak<br><br>Show the field ONLY if:<br>[carbio_bnp] = 1 | B-type natriuretic peptide peak (pg/mL)<br>*Highest value (numerical) during the index hospitalization or ambulatory.* | text (number, Min: 0, Max: 10000000), Required |
| 204 | inhosp_ddimer | Was the patient tested for D dimer? | yesno, Required<br>1 Yes<br>0 No |
| 205 | inhosp_ddimer_peak<br><br>Show the field ONLY if:<br>[inhosp_ddimer] = '1' | D-dimer (ng/mL) peak<br>*Highest value (numerical) during the index hospitalization or ambulatory.* | text (number, Min: 0, Max: 10000000), Required |
| 206 | inhosp_ddimer_peak_date<br><br>Show the field ONLY if:<br>[inhosp_ddimer] = '1' | D-dimer peak date | text (date_dmy, Min: 2020-02-24), Required |
| 207 | inhosp_pt<br><br>Show the field ONLY if:<br>[coordinating_center] = '1' | Was the patient tested for prothrombin time? | yesno, Required<br>1 Yes<br>0 No |
| 208 | inhosp_pt_peak<br><br>Show the field ONLY if:<br>[inhosp_pt] = '1' | Prothrombin time (seconds) peak:<br>*Highest value (numerical) during the index hospitalization or ambulatory.* | text (number, Min: 0, Max: 300), Required |
| 209 | inhosp_pt_peak_date_2<br><br>Show the field ONLY if:<br>[inhosp_pt] = '1' | Prothrombin time (seconds) peak date: | text (date_dmy, Min: 2020-02-24), Required |
| 210 | inhosp_fibri<br><br>Show the field ONLY if:<br>[coordinating_center] = '1' | Was the patient tested for fibrinogen? | yesno, Required<br>1 Yes<br>0 No |
| 211 | inhosp_fibri_lowest<br><br>Show the field ONLY if:<br>[inhosp_fibri] = '1' | Fibrinogen lowest value (g/L):<br>*Lowest value (numerical) during the index hospitalization or ambulatory.* | text (number, Min: 0, Max: 10), Required |
| 212 | inhosp_fibri_lowest_date<br><br>Show the field ONLY if:<br>[inhosp_fibri] = '1' | Fibrinogen lowest value (g/L) date: | text (date_dmy, Min: 2020-02-24), Required |
| 213 | inhosp_hb_low | Section Header: *IN-HOSPITAL - BLOOD COUNT (If Hemoglobin or platelet values are unknown use 999).*<br><br>Hemoglobin (g/L) lowest value<br>*Lowest value (numerical) during the index hospitalization or ambulatory. If unknown use 999.* | text (number, Min: 0, Max: 999), Required |
| 214 | inhosp_hb_lowest_date<br><br>Show the field ONLY if:<br>[coordinating_center] = '1' an d [inhosp_hb_low] < '999' | Hemoglobin (g/L) lowest date | text (date_dmy, Min: 2020-02-24), Required |
| 215 | inhosp_plt_low | Platelet (10x9/L) lowest value<br>*Lowest value (numerical) during the index hospitalization or ambulatory. If unknown use 999.* | text (number, Min: 0, Max: 999), Required |
| 216 | inhosp_plt_lowest_date<br><br>Show the field ONLY if:<br>[coordinating_center] = '1' an d [inhosp_plt_low] < '999' | Platelet (10x9/L) lowest date | text (date_dmy, Min: 2020-02-24), Required |
| 217 | inhosp_lymph | Lymphocyte count (10x9/L) lowest value<br>*Lowest value (numerical) during the index hospitalization or ambulatory. If unknown use 999.* | text (number, Min: 0, Max: 999), Required |
| 218 | inhosp_plt_lowest_date_2<br><br>Show the field ONLY if:<br>[coordinating_center] = '1' an d [inhosp_lymph] < '999' | Lymphocyte count (10x9/L) lowest value date | text (date_dmy, Min: 2020-02-24), Required |

| | | | |
|---|---|---|---|
| 219 | inhosp_cr_peak | Section Header: *IN-HOSPITAL - SERUM BIOCHEMISTRY (If Creatinine or C-reactive protein values are unknown use 999).*<br>Creatinine (mg/dL) highest peak<br>*Highest value (numerical) during the index hospitalization or ambulatory. If unknown use 999.* | text (number, Min: 0, Max: 999), Required |
| 220 | inhosp_cr_peak_date<br>Show the field ONLY if:<br>[coordinating_center] = '1' and [inhosp_cr_peak] < '999' | Creatinine (mg/dL) highest peak date | text (date_dmy, Min: 2020-02-24), Required |
| 221 | inhosp_crp_peak | C-reactive protein (CRP) (mg/dL) peak value<br>*Highest value (numerical) during the index hospitalization or ambulatory. If unknown use 999.* | text (number, Min: 0, Max: 999), Required |
| 222 | inhosp_crp_peak_date<br>Show the field ONLY if:<br>[inhosp_crp_peak] < '999' | C-reactive protein (CRP) (mg/dL) peak date | text (date_dmy, Min: 2020-02-24), Required |
| 223 | biomarkers_complete | Section Header: *Form Status*<br>Complete? | dropdown<br>0 Incomplete<br>1 Unverified<br>2 Complete |

Instrument: **Discharge, Medications & Outcomes** (discharge_medications_outcomes) Collapse

| | | | |
|---|---|---|---|
| 224 | dis_discharge_date<br>Show the field ONLY if:<br>[hospitalization] = '1' | Section Header: *IN-HOSPITAL OUTCOMES*<br>Hospital discharge date<br>*If the patient dies during hospitalization, use the date of death.* | text (date_dmy, Min: 2020-02-01), Required |
| 225 | inhosp_out_mortality<br>Show the field ONLY if:<br>[hospitalization] = '1' | -- IN-HOSPITAL PATIENT MORTALITY --<br>(PRIMARY ENDPOINT) | yesno, Required<br>1 Yes<br>0 No |
| 226 | inhosp_out_mortality_date<br>Show the field ONLY if:<br>[inhosp_out_mortality] = '1' | Date of death | text (date_dmy, Min: 2020-02-01), Required |
| 227 | inhosp_mort_report<br>Show the field ONLY if:<br>[inhosp_out_mortality] = '1' | Anonymize and upload the discharge (mortality) report. Document needed for death adjudication. | file |
| 228 | inhosp_out_cv_death<br>Show the field ONLY if:<br>[inhosp_out_mortality] = '1' | Suspected cardiovascular death | radio<br>1 Death caused by acute MI<br>2 Death caused by sudden cardiac, including unwitnessed<br>3 Death resulting from heart failure<br>4 Death caused by stroke<br>5 Death caused by cardiovascular procedures<br>6 Death resulting from cardiovascular hemorrhage<br>7 Death resulting from other cardiovascular cause |
| 229 | inhosp_out_noncv_death<br>Show the field ONLY if:<br>[inhosp_out_mortality] = '1' | Suspected Non-cardiovascular death | radio<br>1 Death resulting from malignancy<br>2 Death resulting from pulmonary causes<br>3 Death caused by infection (includes sepsis)<br>4 Death resulting from gastrointestinal causes<br>5 Death resulting from accident/trauma<br>6 Death caused by other noncardiovascular organ failure<br>7 Death resulting from other noncardiovascular cause<br>8 Undetermined cause of death |

| | | | |
|---|---|---|---|
| 230 | inhosp_out_acs<br><br>Show the field ONLY if:<br>[hospitalization] = '1' | IN-HOSPITAL ACS?<br>(Acute Coronary Syndrome) | yesno, Required<br>1 Yes<br>0 No |
| 231 | inhosp_out_acs_type<br><br>Show the field ONLY if:<br>[inhosp_out_acs] = '1' | Which type of Acute Coronary Syndrome? | radio, Required<br>1 Unstable angina<br>2 NSTEMI<br>3 STEMI |
| 232 | inhosp_out_acs_date<br><br>Show the field ONLY if:<br>[inhosp_out_acs] = '1' | Date of the acute coronary syndrome | text (date_dmy, Min: 2020-02-01), Required |
| 233 | inhosp_out_stroke<br><br>Show the field ONLY if:<br>[hospitalization] = '1' | IN-HOSPITAL Stroke/transient ischemic attack? | yesno, Required<br>1 Yes<br>0 No |
| 234 | inhosp_out_stroke_type<br><br>Show the field ONLY if:<br>[inhosp_out_stroke] = '1' | Which type of Stroke/transient ischemic attack? | radio, Required<br>1 Transient ischemic attack<br>2 Ischemic stroke<br>3 Hemorrhagic stroke |
| 235 | inhosp_stroke_laterality<br><br>Show the field ONLY if:<br>[inhosp_out_stroke_type] = '2' or [inhosp_out_stroke_type] = '3' | If the patient presented a stroke, specify the laterality | radio, Required<br>1 Right<br>2 left<br>3 Both |
| 236 | inhosp_out_stroke_vessel<br><br>Show the field ONLY if:<br>[inhosp_out_stroke_type] = '2' | If it was an ischemic stroke, specify the type of stroke | radio, Required<br>1 Large vessel stroke (anterior or posterior cerebral circulation, basilar artery, or vertebral artery)<br>2 Small vessel stroke (lacunar) |
| 237 | inhosp_out_stroke_bleed<br><br>Show the field ONLY if:<br>[inhosp_out_stroke_type] = '3' | If it was a hemorrhagic (intracranial bleeding), specify the type | radio<br>1 Epidural<br>2 Subdural<br>3 Subarachnoid<br>4 Intracerebral (Intraparenchymal)<br>5 Intraventricular |
| 238 | inhosp_out_stroke_date<br><br>Show the field ONLY if:<br>[inhosp_out_stroke] = '1' | Date of the stroke/transient ischemic attack | text (date_dmy, Min: 2020-02-01), Required |
| 239 | inhosp_out_vte<br><br>Show the field ONLY if:<br>[hospitalization] = '1' | IN-HOSPITAL Venous/arterial thromboembolism? | yesno, Required<br>1 Yes<br>0 No |
| 240 | inhosp_out_vte_type<br><br>Show the field ONLY if:<br>[inhosp_out_vte] = '1' | If the patient presented venous/arterial thromboembolism, specify which type | radio, Required<br>1 Deep vein thrombosis<br>2 Arterial embolism |
| 241 | inhosp_out_pe_vte_date<br><br>Show the field ONLY if:<br>[inhosp_out_vte] = '1' | Date of venous/arterial thromboembolism | text (date_dmy, Min: 2020-02-01), Required |
| 242 | inhosp_out_vte_describe<br><br>Show the field ONLY if:<br>[inhosp_out_vte] = '1' | If the patient presented a venous/arterial thromboembolism, describe the location and if invasive treatment was needed. | notes |
| 243 | inhosp_out_pe<br><br>Show the field ONLY if:<br>[hospitalization] = '1' | IN-HOSPITAL Pulmonary embolism<br>*Definition requires confirmation by Thorax CT scan.* | yesno, Required<br>1 Yes<br>0 No |

| | | | |
|---|---|---|---|
| 244 | inhosp_out_pe_laterality<br>Show the field ONLY if:<br>[inhosp_out_pe] = '1' | If the patient had a Pulmonary embolism, specify the laterality | radio, Required<br>1 Right<br>2 left<br>3 Both |
| 245 | inhosp_out_pe_vessel<br>Show the field ONLY if:<br>[inhosp_out_pe] = '1' | If the patient had a Pulmonary embolism, specify which type | radio, Required<br>1 Major vessel (Main artery, Lobar artery, Segmental)<br>2 Minor vessel (Sub-segmental) |
| 246 | inhosp_out_pe_date<br>Show the field ONLY if:<br>[inhosp_out_pe] = '1' | Date of Pulmonary embolism | text (date_dmy, Min: 2020-02-01), Required |
| 247 | inhosp_out_bleeding<br>Show the field ONLY if:<br>[hospitalization] = '1' | IN-HOSPITAL major bleeding? | yesno, Required<br>1 Yes<br>0 No |
| 248 | inhosp_out_bleeding_type<br>Show the field ONLY if:<br>[inhosp_out_bleeding] = '1' | If the patient presented a major bleeding, specify which type? | radio, Required<br>1 Bleeding with an Hb drop between 3-5 g/dL.<br>2 Bleeding requiring transfusion<br>3 Bleeding requiring surgical intervention<br>4 Non-fatal intracranial hemorrhage<br>5 Fatal bleeding without autopsy/imaging confirmation<br>6 Fatal bleeding with autopsy/imaging confirmation |
| 249 | inhosp_out_bleeding_date<br>Show the field ONLY if:<br>[inhosp_out_bleeding] = '1' | Date of major bleeding | text (date_dmy, Min: 2020-02-01), Required |
| 250 | inhosp_out_bleeding_site<br>Show the field ONLY if:<br>[inhosp_out_bleeding] = '1' | Specify the site of origin if the major bleeding: | radio, Required<br>1 Intracraneal or intraspinal<br>2 Pulmonary (hemoptysis, endotracheal tube, bronchoscopy)<br>3 Gastrointestinal (hematemesis, melena, upper endoscopy or colonoscopy)<br>4 Genito-urinary (urine, urinary catheter, cystoscopy)<br>5 Nose (Epistaxis)<br>6 Soft tissue hematoma<br>7 Solid organ hematoma<br>8 Ocular<br>9 Intra-abdominal bleeding<br>10 Retroperitoneal bleeding<br>11 Other |
| 251 | inhos_out_bleeding_other<br>Show the field ONLY if:<br>[inhosp_out_bleeding_site]=11 | If other, describe the site of origin: | text, Required |
| 252 | inhosp_bleeding_descrip<br>Show the field ONLY if:<br>[inhosp_out_bleeding] = '1' | Provide a very small description of the bleeding event | notes, Required |
| 253 | inhosp_out_transfusion<br>Show the field ONLY if:<br>[hospitalization] = '1' | IN-HOSPITAL Red blood cell transfusion<br>*At least one red blood cell transfusion* | yesno, Required<br>1 Yes<br>0 No |

| | | | |
|---|---|---|---|
| 254 | inhosp_out_arrhyth<br><br>Show the field ONLY if:<br>[hospitalization] = '1' | IN-HOSPITAL Serious arrhythmias events | yesno, Required<br>1 Yes<br>0 No |
| 255 | inhosp_out_arrhyth_type<br><br>Show the field ONLY if:<br>[inhosp_out_arrhyth] = '1' | If the patient presented serious arrhythmias events, specify which | radio, Required<br>1 Bradicardya requiring iv Medication<br>2 Bradicardya requiring pacemaker<br>3 Supraventricular tachycardia requiring iv Medication<br>4 Supraventricular tachycardia requiring Cardioversion<br>5 Ventricular tachycardia requiring iv Medication<br>6 Ventricular tachycardia requiring Cardioversion |
| 256 | inhosp_out_arrhyth_date<br><br>Show the field ONLY if:<br>[inhosp_out_arrhyth] = '1' | Date of presentation of cardiac arrhythmia | text (date_dmy, Min: 2020-02-01), Required |
| 257 | inhosp_other_events<br><br>Show the field ONLY if:<br>[hospitalization] = '1' | If there was another relevant event, specify which.<br>*Use a comma to separate the complications. Example: urgent surgery, etc.* | notes |
| 258 | dis_aspirin<br><br>Show the field ONLY if:<br>[hospitalization] = '1' and [inhosp_out_mortality] = '0' | Section Header: *DISCHARGE - TREATMENT*<br>Aspirin | yesno, Required<br>1 Yes<br>0 No |
| 259 | dis_p2y12<br><br>Show the field ONLY if:<br>[hospitalization] = '1' and [inhosp_out_mortality] = '0' | P2Y12 inhibitor<br>*Example: clopidogrel, prasugrel, Ticagrelor* | yesno, Required<br>1 Yes<br>0 No |
| 260 | dis_p2y12_type<br><br>Show the field ONLY if:<br>[dis_p2y12] = '1' | Which P2Y12 inhibitor | radio, Required<br>1 Clopidogrel<br>2 Prasugrel<br>3 Ticagrelor |
| 261 | dis_ace_inh<br><br>Show the field ONLY if:<br>[hospitalization] = '1' and [inhosp_out_mortality] = '0' | ACE inhibitor<br>*Angiotensin-converting-enzyme inhibitors (ACE inhibitors)* | yesno, Required<br>1 Yes<br>0 No |
| 262 | dis_ace_type<br><br>Show the field ONLY if:<br>[dis_ace_inh] = '1' | ACE inhibitor type | radio, Required<br>1 Lisinopril<br>2 Enalapril<br>3 Perindopril<br>4 Other |
| 263 | dis_ace_other<br><br>Show the field ONLY if:<br>[dis_ace_type] = '4' | If other ACE inhibitor, specify which | text, Required |
| 264 | dis_ace_dose<br><br>Show the field ONLY if:<br>[dis_ace_inh] = '1' | ACE inhibitor TOTAL daily dose (mg)<br>*If unknown use 999.* | text (number, Min: 1, Max: 999), Required |
| 265 | dis_arbs<br><br>Show the field ONLY if:<br>[hospitalization] = '1' and [inhosp_out_mortality] = '0' | ARBs<br>*Angiotensin II receptor blockers* | yesno, Required<br>1 Yes<br>0 No |

| | | | |
|---|---|---|---|
| 266 | dis_arbs_type<br><br>Show the field ONLY if:<br>[dis_arbs] = '1' | ARBs type | radio, Required<br>1 Losartan<br>2 Candesartan<br>3 Irbesartan<br>4 Telmisartan<br>5 Valsartan<br>6 Olmesartan<br>7 Other |
| 267 | dis_arbs_other<br><br>Show the field ONLY if:<br>[dis_arbs_type] = '7' | If other ARBs, specify which | text, Required |
| 268 | dis_abrs_dose<br><br>Show the field ONLY if:<br>[dis_arbs] = '1' | ARBs TOTAL daily dose (mg)<br>*If unknown use 999.* | text (number, Min: 0, Max: 999), Required |
| 269 | dis_arni<br><br>Show the field ONLY if:<br>[hospitalization] = '1' and [inh osp_out_mortality] = '0' | Angiotensin receptor neprilysin inhibitor (ARNI)<br>*Sacubitril/valsartan (Entresto)* | yesno, Required<br>1 Yes<br>0 No |
| 270 | dis_arni_dose<br><br>Show the field ONLY if:<br>[dis_arni] = '1' | Angiotensin receptor neprilysin inhibitor (ARNI) total daily dose (mg)<br>*Sacubitril/valsartan (Entresto). If unknown use 999.* | text (number, Min: 0, Max: 999), Required |
| 271 | dis_statins<br><br>Show the field ONLY if:<br>[hospitalization] = '1' and [inh osp_out_mortality] = '0' | Statins | yesno, Required<br>1 Yes<br>0 No |
| 272 | dis_statin_type<br><br>Show the field ONLY if:<br>[dis_statins] = '1' | Statin type | radio, Required<br>1 Atorvastatin<br>2 Rosuvastatin<br>3 Simvastatin<br>4 Other |
| 273 | dis_statin_other<br><br>Show the field ONLY if:<br>[dis_statin_type] = '4' | If other statin, specify which | text, Required |
| 274 | dis_statin_dose<br><br>Show the field ONLY if:<br>[dis_statins] = '1' | Statin TOTAL daily dose (mg)<br>*If unknown use 999.* | text (number, Min: 0, Max: 999), Required |
| 275 | dis_beta_block<br><br>Show the field ONLY if:<br>[hospitalization] = '1' and [inh osp_out_mortality] = '0' | Beta Blocker | yesno, Required<br>1 Yes<br>0 No |
| 276 | dis_beta_block_type<br><br>Show the field ONLY if:<br>[dis_beta_block] = '1' | Beta Blocker type | radio, Required<br>1 Bisoprolol<br>2 Atenolol<br>3 Metoprolol<br>4 Carvedilol<br>5 Other |
| 277 | dis_beta_block_other<br><br>Show the field ONLY if:<br>[dis_beta_block_type] = '5' | If other Beta blocker, specify which | text, Required |
| 278 | dis_beta_block_dose<br><br>Show the field ONLY if:<br>[dis_beta_block] = '1' | Beta Blocker TOTAL daily dose (mg)<br>*If unknown use 999.* | text (number, Min: 0, Max: 999), Required |

| | | | |
|---|---|---|---|
| 279 | dis_ccb<br><br>Show the field ONLY if:<br>[hospitalization] = '1' and [inhosp_out_mortality] = '0' | Calcium channel blocker | yesno, Required<br>1 Yes<br>0 No |
| 280 | dis_ccb_type<br><br>Show the field ONLY if:<br>[dis_ccb] = '1' | Calcium channel blockers type | radio, Required<br>1 Amlodipine<br>2 Diltiazem<br>3 Nicardipine<br>4 Nifedipine<br>5 Verapamil<br>6 Other |
| 281 | dis_ccb_other<br><br>Show the field ONLY if:<br>[dis_ccb_type] = '6' | If other calcium channel blocker, specify which | text, Required |
| 282 | dis_ccb_dose<br><br>Show the field ONLY if:<br>[dis_ccb] = '1' | Calcium channel blocker TOTAL daily dose (mg)<br>*If unknown use 999.* | text (number, Min: 0, Max: 999), Required |
| 283 | dis_loop_diuretics<br><br>Show the field ONLY if:<br>[hospitalization] = '1' and [inhosp_out_mortality] = '0' | Loop diuretic<br>*Example: Furosemide, Torsemide, etc.* | yesno, Required<br>1 Yes<br>0 No |
| 284 | dis_loop_diuretic_dose<br><br>Show the field ONLY if:<br>[dis_loop_diuretics] = '1' | Loop diuretic TOTAL daily dose (mg)<br>*If unknown use 999.* | text (number, Min: 0, Max: 999), Required |
| 285 | dis_mras<br><br>Show the field ONLY if:<br>[hospitalization] = '1' and [inhosp_out_mortality] = '0' | Mineralocorticoid receptor antagonists (MRAs)<br>*Spironolactone or eplerenone* | yesno, Required<br>1 Yes<br>0 No |
| 286 | dis_mras_type<br><br>Show the field ONLY if:<br>[dis_mras] = '1' | Mineralocorticoid receptor antagonists type | radio, Required<br>1 Spironolactone<br>2 Eplerenone |
| 287 | dis_mras_dose<br><br>Show the field ONLY if:<br>[dis_mras] = '1' | Mineralocorticoid receptor antagonists (MRAs) TOTAL daily dose (mg)<br>*If unknown use 999.* | text (number, Min: 0, Max: 999), Required |
| 288 | dis_ppis<br><br>Show the field ONLY if:<br>[hospitalization] = '1' and [inhosp_out_mortality] = '0' | Proton-pump inhibitors | yesno, Required<br>1 Yes<br>0 No |
| 289 | dis_oral_hypo_glc<br><br>Show the field ONLY if:<br>[hospitalization] = '1' and [inhosp_out_mortality] = '0' | Oral hypoglycemic agents | yesno, Required<br>1 Yes<br>0 No |
| 290 | dis_insulin<br><br>Show the field ONLY if:<br>[hospitalization] = '1' and [inhosp_out_mortality] = '0' | Insulin | yesno, Required<br>1 Yes<br>0 No |
| 291 | dis_anticoagulant<br><br>Show the field ONLY if:<br>[hospitalization] = '1' and [inhosp_out_mortality] = '0' | Was the patient treated with any anticoagulant therapy?<br>*Example: acenocoumarol, enoxaparin, rivaroxaban, etc.* | yesno, Required<br>1 Yes<br>0 No |
| 292 | dis_anticoagulant_type<br><br>Show the field ONLY if:<br>[dis_anticoagulant] = '1' | Which type of Anticoagulant treatment?<br>**Example: Rivaroxaban, dabigatran, apixaban, or edoxaban.* | radio, Required<br>1 Vitamin K antagonists (acenocoumarol)<br>2 Directly acting oral anticoagulants*<br>3 Low-molecular-weight heparin (Enoxaparin, etc.) |

| | | | |
|---|---|---|---|
| 293 | dis_anticoagulant_type_2nd<br><br>Show the field ONLY if:<br>[dis_anticoagulant] = '1' | If the patient was treated with a second anticoagulant treatment, specify which<br>**Example: Rivaroxaban, dabigatran, apixaban, or edoxaban.* | radio<br>1 Vitamin K antagonists (acenocoumarol)<br>2 Directly acting oral anticoagulants*<br>3 Low-molecular-weight heparin (Enoxaparin, etc.) |
| 294 | dis_anticoagulant_why<br><br>Show the field ONLY if:<br>[dis_anticoagulant] = '1' | Clinical indication for anticoagulant treatment | radio, Required<br>1 Prophylaxis<br>2 Atrial fibrillation<br>3 Venous thromboembolism<br>4 Pulmonary embolism<br>5 Arterial embolism<br>6 Stroke<br>7 Cardiac intraventricular thrombus<br>8 Other |
| 295 | dis_anticoagulant_other<br><br>Show the field ONLY if:<br>[dis_anticoagulant_why] = '8' | If other indication for anticoagulant treatment, specify which | text |
| 296 | dis_anticoagulant_why_2<br><br>Show the field ONLY if:<br>[dis_anticoagulant] = '1' | If there was a second clinical indication for anticoagulant treatment, specify which | radio<br>1 Prophylaxis<br>2 Atrial fibrillation<br>3 Venous thromboembolism<br>4 Pulmonary embolism<br>5 Arterial embolism<br>6 Stroke<br>7 Cardiac intraventricular thrombus |
| 297 | dis_lwmh_tx_type<br><br>Show the field ONLY if:<br>[dis_anticoagulant_type] = '3' or [dis_anticoagulant_type_2nd] = '3' | Which Low-molecular-weight heparin was used? | radio, Required<br>1 Enoxaparin<br>2 Dalteparin<br>3 Bemiparin<br>4 Tinzaparin<br>5 Fondaparinux<br>6 Nadroparin<br>7 Certoparin<br>8 Other |
| 298 | dis_anticoag_other<br><br>Show the field ONLY if:<br>[dis_lwmh_tx_type] = '8' | If other Low-molecular-weight heparin, specify which | text, Required |
| 299 | dis_lmwh_dose<br><br>Show the field ONLY if:<br>[dis_anticoagulant_type] = '3' or [dis_anticoagulant_type_2nd] = '3' | Specify the highest dose of Low-molecularweight heparin (only numbers) that was used.<br>*Example: 40 mg or 4000 UI (only numbers).* | text (number, Min: 0, Max: 10000000) |
| 300 | dis_lmwh_unit<br><br>Show the field ONLY if:<br>[dis_anticoagulant_type] = '3' or [dis_anticoagulant_type_2nd] = '3' | Which was the Low-molecular-weight heparin Unit? | radio, Required<br>1 mg<br>2 UI |
| 301 | dis_lmwh_frequency<br><br>Show the field ONLY if:<br>[dis_anticoagulant_type] = '3' or [dis_anticoagulant_type_2nd] = '3' | Which was the frequency of administration of the Low-molecular-weight heparin? | radio<br>1 Each 24 hours<br>2 Each 12 hours |

| | | | |
|---|---|---|---|
| 302 | dis_noac_type<br><br>Show the field ONLY if:<br>[dis_anticoagulant_type] = '2' or [dis_anticoagulant_type_2nd] = '2' | If Directly acting oral anticoagulants were used, specify which | radio, Required<br>1 Rivaroxaban (Xarelto)<br>2 Dabigatran (Pradaxa)<br>3 Apixaban (Eliquis)<br>4 Edoxaban (Lixiana) |
| 303 | dis_noac_dose<br><br>Show the field ONLY if:<br>[dis_anticoagulant_type] = '2' or [dis_anticoagulant_type_2nd] = '2' | Specify the Directly acting oral anticoagulants TOTAL Daily dose (mg)<br>*If unknown use 999.* | text (number, Min: 0, Max: 999), Required |
| 304 | dis_nsaids<br><br>Show the field ONLY if:<br>[hospitalization] = '1' and [inhosp_out_mortality] = '0' | Nonsteroidal anti-inflammatory drugs (NSAIDs) | yesno, Required<br>1 Yes<br>0 No |
| 305 | dis_nsaids_type<br><br>Show the field ONLY if:<br>[dis_nsaids] ='1' | NSAIDs type | radio, Required<br>1 Ibuprofen<br>2 Diclofenac<br>3 Naproxen<br>4 Dexketoprofen or ketoprofen<br>5 Celecoxib<br>6 Other |
| 306 | dis_nsaids_dose<br><br>Show the field ONLY if:<br>[dis_nsaids] ='1' | NSAIDs total daily dose (mg)<br>*If unknown use 9999.* | text (number, Min: 0, Max: 9999), Required |
| 307 | dis_cortis<br><br>Show the field ONLY if:<br>[hospitalization] = '1' and [inhosp_out_mortality] = '0' | Corticosteroids therapy<br>*Example: Methylprednisolone, prednisone, dexamethasone, etc.* | yesno, Required<br>1 Yes<br>0 No |
| 308 | dis_cortis_type<br><br>Show the field ONLY if:<br>[dis_cortis] ='1' | Specify which type of Corticosteroids | radio, Required<br>1 Methylprednisolone<br>2 Dexamethasone<br>3 Prednisone<br>4 Prednisolone<br>5 Hydrocortisone<br>6 Other |
| 309 | dis_cortis_other<br><br>Show the field ONLY if:<br>[dis_cortis_type] = '6' | If other corticosteroids, specify which: | text, Required |
| 310 | dis_cortis_dose<br><br>Show the field ONLY if:<br>[dis_cortis] ='1' | Specify the dose of corticosteroids (mg)?<br>*Example: 20 mg (only numbers).* | text |
| 311 | dis_another_tx<br><br>Show the field ONLY if:<br>[hospitalization] = '1' and [inhosp_out_mortality] = '0' | If there was any other relevant medication, specify which<br>*Use a comma to separate.* | notes |
| 312 | discharge_medications_outcomes_complete | Section Header: *Form Status*<br>Complete? | dropdown<br>0 Incomplete<br>1 Unverified<br>2 Complete |

Instrument: **@1-year Outcomes & Medications** (year_outcomes_medications) Collapse

| | | | |
|---|---|---|---|
| 313 | fu1y_done | Section Header: *@1-year FOLLOW-UP OUTCOMES*<br>1-year follow-up was performed | yesno, Required<br>1 Yes<br>0 No |
| 314 | lost_to_fup<br>Show the field ONLY if:<br>[fu1y_done] ='0' | Lost to follow-up | yesno, Required<br>1 Yes<br>0 No |
| 315 | lost_to_fup_vital_status<br>Show the field ONLY if:<br>[fu1y_done] ='0' | Is there is any data available? | dropdown, Required<br>1 Complete lost to follow-up<br>2 Alive but the rest of event were no assessed.<br>3 Death but the rest of event were no assessed. |
| 316 | fu1y_date<br>Show the field ONLY if:<br>[fu1y_done] ='1' | Date of Follow-up | text (date_dmy, Min: 2020-02-01), Required |
| 317 | fu1y_new_swab<br>Show the field ONLY if:<br>[fu1y_done] ='1' | Did the patient has undergone a new Coronavirus swab?<br>*Between baseline and follow-up.* | yesno, Required<br>1 Yes<br>0 No |
| 318 | fu1y_new_swab_date<br>Show the field ONLY if:<br>[fu1y_done] ='1' and [fu1y_new_swab] = '1' | If the patient performed a new Coronavirus swab, indicate the date | text (date_dmy, Min: 2020-02-01), Required |
| 319 | fu1y_new_swab_result<br>Show the field ONLY if:<br>[fu1y_done] ='1' and [fu1y_new_swab] = '1' | If the patient has undergone a new swab, specify the result. | radio, Required<br>0 Negative<br>1 Positive |
| 320 | fu1y_antibody_test<br>Show the field ONLY if:<br>[fu1y_done] ='1' | Did the patient has undergone a Coronavirus antibody test?<br>*Between baseline and follow-up.* | yesno, Required<br>1 Yes<br>0 No |
| 321 | fu1y_antibody_date<br>Show the field ONLY if:<br>[fu1y_done] ='1' and [fu1y_antibody_test] = '1' | If the patient has undergone a Coronavirus antibody test, specify the date | text (date_dmy, Min: 2020-02-01), Required |
| 322 | fu1y_antibody_igg<br>Show the field ONLY if:<br>[fu1y_done] ='1' and [fu1y_antibody_test] = '1' | If the patient has undergone an antibody test, specify the result of IgG. | radio<br>0 Negative<br>1 Positive |
| 323 | fu1y_antibody_igm<br>Show the field ONLY if:<br>[fu1y_done] ='1' and [fu1y_antibody_test] = '1' | If the patient has undergone an antibody test, specify the result of IgM. | radio<br>0 Negative<br>1 Positive |
| 324 | fu1y_antibody_iga<br>Show the field ONLY if:<br>[fu1y_done] ='1' and [fu1y_antibody_test] = '1' | If the patient has undergone an antibody test, specify the result of IgA. | radio<br>0 Negative<br>1 Positive |
| 325 | fu1y_vaccine<br>Show the field ONLY if:<br>[fu1y_done] ='1' and [coordinating_center] = '1' | Does the patient was vaccinated? | yesno, Required<br>1 Yes<br>0 No |
| 326 | fu1y_vaccine_date<br>Show the field ONLY if:<br>[fu1y_vaccine]='1' | Date of the first dose | text (date_dmy, Min: 2020-02-01), Required |

| | | | |
|---|---|---|---|
| 327 | fu1y_vaccine_type<br><br>Show the field ONLY if:<br>[fu1y_vaccine]='1' | Which vaccine was used? | radio, Required<br>1 Pfizer<br>2 Moderna<br>3 AstraZeneca<br>4 Janssen<br>5 Other |
| 328 | fu1y_vaccione_type_other<br><br>Show the field ONLY if:<br>[fu1y_vaccine_type]='5' | If other, specify | text, Required |
| 329 | fu1y_vaccine_doses<br><br>Show the field ONLY if:<br>[fu1y_vaccine]='1' | Is the vaccination regimen complete? | radio, Required<br>1 One dose (only for 2 doses vaccines: Pfizer, Moderna or AstraZeneca)<br>2 Full vaccinated |
| 330 | fu1y_rehosp<br><br>Show the field ONLY if:<br>[fu1y_done] ='1' | Section Header: *RE-HOSPITALIZATION*<br><br>RE-HOSPITALIZATION | yesno, Required<br>1 Yes<br>0 No |
| 331 | fu1y_hosp_cause<br><br>Show the field ONLY if:<br>[fu1y_rehosp] = '1' | Did the re-hospitalization was related to a COVID-19?<br>*Consider any complication or sequelae related to COVID-19.* | yesno, Required<br>1 Yes<br>0 No |
| 332 | fu1y_rehosp_specify<br><br>Show the field ONLY if:<br>[fu1y_rehosp] = '1' | Specify the diagnosis (diagnosis/specialty) of the re-hospitalization?<br>*Use a comma to separate. Example: stroke,neurology* | notes |
| 333 | fu1y_rehosp_quantity<br><br>Show the field ONLY if:<br>[fu1y_rehosp] ='1' | If the patient had more than one hospitalization, specify the number. | text (number, Min: 2, Max: 100) |
| 334 | fu1y_rehosp_icu<br><br>Show the field ONLY if:<br>[fu1y_done] ='1' and [fu1y_rehosp] = '1' | Did the patient require admission in an intensive care unit?<br>*In any re-hospitalization.* | yesno, Required<br>1 Yes<br>0 No |
| 335 | fu1y_rehosp_imv<br><br>Show the field ONLY if:<br>[fu1y_rehosp_icu] = '1' | Mechanical ventilation<br>*In any re-hospitalization.* | yesno, Required<br>1 Yes<br>0 No |
| 336 | fu1y_out_mortality<br><br>Show the field ONLY if:<br>[fu1y_done] ='1' | Section Header: *@1-YEAR OUTCOMES*<br><br>-- 1-YEAR PATIENT MORTALITY --<br>(PRIMARY ENDPOINT) | yesno, Required<br>1 Yes<br>0 No |
| 337 | fu1y_out_mortality_date<br><br>Show the field ONLY if:<br>[fu1y_out_mortality] = '1' | Day of death | text (date_dmy, Min: 2020-02-01), Required |
| 338 | fu1y_out_cv_death<br><br>Show the field ONLY if:<br>[fu1y_out_mortality] = '1' | Suspected cardiovascular death | radio<br>1 Death caused by acute myocardial infarction<br>2 Death caused by sudden cardiac, including unwitnessed<br>3 Death resulting from heart failure<br>4 Death caused by stroke<br>5 Death caused by cardiovascular procedures<br>6 Death resulting from cardiovascular hemorrhage<br>7 Death resulting from other cardiovascular cause |

| | | | |
|---|---|---|---|
| 339 | fu1y_out_noncv_death<br><br>Show the field ONLY if:<br>[fu1y_out_mortality] = '1' | Suspected Non-cardiovascular death | radio<br>1 Death resulting from malignancy<br>2 Death resulting from pulmonary causes<br>3 Death caused by infection (includes sepsis)<br>4 Death resulting from gastrointestinal causes<br>5 Death resulting from accident/trauma<br>6 Death caused by other noncardiovascular organ failure<br>7 Death resulting from other noncardiovascular cause<br>8 Undetermined cause of death |
| 340 | fu1y_death_report<br><br>Show the field ONLY if:<br>[fu1y_out_mortality] = '1' | Anonymize and upload the discharge (mortality) report. Document needed for death adjudication. | file |
| 341 | fu1y_out_acs<br><br>Show the field ONLY if:<br>[fu1y_done] ='1' | 1-YEAR FOLLOW-UP ACS<br>(Acute Coronary Syndrome) | yesno, Required<br>1 Yes<br>0 No |
| 342 | fu1y_out_acs_type<br><br>Show the field ONLY if:<br>[fu1y_out_acs] = '1' | Which type of Acute Coronary Syndrome? | radio, Required<br>1 Unstable angina<br>2 NSTEMI<br>3 STEMI |
| 343 | fu1y_out_acs_date<br><br>Show the field ONLY if:<br>[fu1y_out_acs] = '1' | Date of the ACS? | text (date_dmy, Min: 2020-02-01), Required |
| 344 | fu1y_out_revasc<br><br>Show the field ONLY if:<br>[fu1y_done] ='1' | 1-YEAR FOLLOW-UP CORONARY REVASCULARIZATION | yesno, Required<br>1 Yes<br>0 No |
| 345 | fu1y_out_revasc_type<br><br>Show the field ONLY if:<br>[fu1y_out_revasc] = '1' | Which type of coronary Revascularization? | radio, Required<br>1 PCI<br>2 CABG |
| 346 | fu1y_out_revasc_date<br><br>Show the field ONLY if:<br>[fu1y_out_revasc] = '1' | Date of revascularization | text (date_dmy, Min: 2020-02-01), Required |
| 347 | fu1y_out_tlr<br><br>Show the field ONLY if:<br>[fu1y_out_revasc] = '1' | If the patient had a revascularization, it was a target lesion revascularization?<br>*Target lesion revascularization, refers to a stent implanted during the index hospitalization* | yesno, Required<br>1 Yes<br>0 No |
| 348 | fu1y_out_stent_thrombo<br><br>Show the field ONLY if:<br>[fu1y_out_revasc] = '1' | If the patient had revascularization, it was related to a stent thrombosis? | yesno, Required<br>1 Yes<br>0 No |
| 349 | fu1y_stroke<br><br>Show the field ONLY if:<br>[fu1y_done] ='1' | 1-YEAR FOLLOW-UP STROKE/TIA<br>*TIA: transient ischemic attack* | yesno, Required<br>1 Yes<br>0 No |
| 350 | fu1y_out_stroke_type<br><br>Show the field ONLY if:<br>[fu1y_stroke] = '1' | Which type of Stroke/transient ischemic attack the patient had? | radio, Required<br>1 Ischemic stroke<br>2 Transient ischemic attack<br>3 Hemorrhagic stroke |
| 351 | fu1y_out_stroke_date<br><br>Show the field ONLY if:<br>[fu1y_stroke] = '1' | Date of the Stroke/transient ischemic attack | text (date_dmy, Min: 2020-02-01), Required |

| | | | |
|---|---|---|---|
| 352 | fu1y_out_hf<br><br>Show the field ONLY if:<br>[fu1y_done] ='1' | 1-YEAR FOLLOW-UP HEART FAILURE HOSPITALIZATION | yesno, Required<br>1 Yes<br>0 No |
| 353 | fu1y_out_hf_nyha<br><br>Show the field ONLY if:<br>[fu1y_out_hf] = '1' | Which class of New York Heart Association did the patient presented?<br>*NYHA: New York Heart Association.* | radio, Required<br>1 NYHA I<br>2 NYHA II<br>3 NYHA III<br>4 NYHA IV |
| 354 | fu1y_out_ahf_date<br><br>Show the field ONLY if:<br>[fu1y_out_hf] = '1' | Date of Heart failure hospitalization admission. | text (date_dmy, Min: 2020-02-01), Required |
| 355 | fu1y_out_vte<br><br>Show the field ONLY if:<br>[fu1y_done] ='1' | 1-YEAR FOLLOW-UP VENOUS/ARTERIAL THROMBOEMBOLISM | yesno, Required<br>1 Yes<br>0 No |
| 356 | fu1y_out_vte_type<br><br>Show the field ONLY if:<br>[fu1y_out_vte] = '1' | Which type of venous/arterial thromboembolism? | radio, Required<br>1 Deep vein thrombosis<br>2 Arterial embolism |
| 357 | fu1y_out_vte_date<br><br>Show the field ONLY if:<br>[fu1y_out_vte] = '1' | Date of Venous/arterial thromboembolism | text (date_dmy, Min: 2020-02-01), Required |
| 358 | fu1_out_vte_describe<br><br>Show the field ONLY if:<br>[fu1y_out_vte] = '1' | If the patient presented a venous/arterial thromboembolism, describe the location and if invasive treatment was needed.<br>*Use a comma to separate the diagnosis, location, and treatment.* | notes |
| 359 | fu1y_out_pe<br><br>Show the field ONLY if:<br>[fu1y_done] ='1' | 1-YEAR FOLLOW-UP PULMONARY EMBOLISM | yesno, Required<br>1 Yes<br>0 No |
| 360 | fu1y_out_pe_laterality<br><br>Show the field ONLY if:<br>[fu1y_out_pe] = '1' | If the patient had a Pulmonary embolism, specify the laterality | radio, Required<br>1 Right<br>2 left<br>3 Both |
| 361 | fu1y_out_pe_vessel<br><br>Show the field ONLY if:<br>[fu1y_out_pe] = '1' | If the patient had a Pulmonary embolism, specify which type | radio, Required<br>1 Major vessel (Main artery, Lobar artery, Segmental)<br>2 Minor vessel (Sub-segmental) |
| 362 | nhosp_out_pe_date<br><br>Show the field ONLY if:<br>[fu1y_out_pe] = '1' | Date of Pulmonary embolism | text (date_dmy, Min: 2020-02-01), Required |
| 363 | fu1y_out_bleeding<br><br>Show the field ONLY if:<br>[fu1y_done] ='1' | 1-YEAR FOLLOW-UP MAJOR BLEEDING | yesno, Required<br>1 Yes<br>0 No |
| 364 | fu1y_out_bleeding_type<br><br>Show the field ONLY if:<br>[fu1y_out_bleeding] = '1' | If the patient presented a major bleeding, specify which type? | radio, Required<br>1 Bleeding with an Hb drop between 3-5 g/dL.<br>2 Bleeding requiring transfusion<br>3 Bleeding requiring surgical intervention<br>4 Non-fatal intracranial hemorrhage<br>5 Fatal bleeding without autopsy/imaging confirmation<br>6 Fatal bleeding with autopsy/imaging confirmation |

| | | | |
|---|---|---|---|
| 365 | fu1y_out_bleeding_date<br><br>Show the field ONLY if:<br>[fu1y_out_bleeding] = '1' | Date of Major bleeding | text (date_dmy, Min: 2020-02-01), Required |
| 366 | fu1y_out_bleeding_site<br><br>Show the field ONLY if:<br>[fu1y_out_bleeding] = '1' | Specify the site of origin if the major bleeding: | radio, Required<br>1 Intracraneal or intraspinal<br>2 Pulmonary (hemoptysis, endotracheal tube, bronchoscopy)<br>3 Gastrointestinal (hematemesis, melena, upper endoscopy or colonoscopy)<br>4 Genito-urinary (urine, urinary catheter, cystoscopy)<br>5 Nose (Epistaxis)<br>6 Soft tissue hematoma<br>7 Solid organ hematoma<br>8 Ocular<br>9 Intra-abdominal bleeding<br>10 Retroperitoneal bleeding<br>11 Other |
| 367 | fu1y_out_bleeding_other<br><br>Show the field ONLY if:<br>[fu1y_out_bleeding_site]=11 | If other, describe the site of origin: | text, Required |
| 368 | fu1y_bleeding_descrip<br><br>Show the field ONLY if:<br>[fu1y_out_bleeding] = '1' | Provide a very small description of the bleeding event | notes, Required |
| 369 | fu1y_out_transfusion<br><br>Show the field ONLY if:<br>[fu1y_done] ='1' | 1-YEAR FOLLOW-UP RED BLOOD CELL TRANSFUSION | yesno, Required<br>1 Yes<br>0 No |
| 370 | fu1y_out_arrhyth<br><br>Show the field ONLY if:<br>[fu1y_done] ='1' | 1-YEAR FOLLOW-UP SERIOUS ARRHYTHMIAS EVENTS | yesno, Required<br>1 Yes<br>0 No |
| 371 | fu1y_out_arrhyth_type<br><br>Show the field ONLY if:<br>[fu1y_out_arrhyth] = '1' | If the patient presented serious arrhythmias events, specify which | radio, Required<br>1 Bradicardya requiring pacemaker<br>2 New onset of atrial fibrillation or flutter<br>3 Any episode of Ventricular tachycardia |
| 372 | fu1y_out_arrhyth_date<br><br>Show the field ONLY if:<br>[fu1y_out_arrhyth] = '1' | Date of presentation of Cardiac arrhythmia | text (date_dmy, Min: 2020-02-01), Required |
| 373 | fu1y_lvef<br><br>Show the field ONLY if:<br>[fu1y_done] = '1' | Left-Ventricular Ejection Fraction (%) (LVEF)<br>*Assessed by any method. If unknown use 999.* | text (number, Min: 0, Max: 999), Required |
| 374 | fu1y_other_events<br><br>Show the field ONLY if:<br>[fu1y_done] ='1' | If there was another relevant event, specify which.<br>*Use a comma to separate the complications. Example: urgent surgery, etc.* | notes |
| 375 | fu1y_aspirin<br><br>Show the field ONLY if:<br>[fu1y_done] ='1' and [fu1y_out_mortality] = '0' | Section Header: *@1-year FOLLOW-UP MEDICATIONS*<br>Aspirin | yesno, Required<br>1 Yes<br>0 No |
| 376 | fu1y_p2y12<br><br>Show the field ONLY if:<br>[fu1y_done] ='1' and [fu1y_out_mortality] = '0' | P2Y12 inhibitor | yesno, Required<br>1 Yes<br>0 No |

| | | | |
|---|---|---|---|
| 377 | fu1y_p2y12_type<br><br>Show the field ONLY if:<br>[fu1y_p2y12] = '1' | Which P2Y12 inhibitor | radio, Required<br>1 Clopidogrel<br>2 Prasugrel<br>3 Ticagrelor |
| 378 | fu1y_ace_inh<br><br>Show the field ONLY if:<br>[fu1y_done] ='1' and [fu1y_out_mortality] = '0' | ACE inhibitor<br>*Angiotensin-converting-enzyme inhibitors (ACE inhibitors)* | yesno, Required<br>1 Yes<br>0 No |
| 379 | fu1y_ace_type<br><br>Show the field ONLY if:<br>[fu1y_ace_inh] = '1' | ACE inhibitor type | radio, Required<br>1 Lisinopril<br>2 Enalapril<br>3 Perindopril<br>4 Other |
| 380 | fu1y_ace_inh_other<br><br>Show the field ONLY if:<br>[fu1y_ace_type] = '4' | If other ACE inhibitor specify | text, Required |
| 381 | fu1y_ace_dose<br><br>Show the field ONLY if:<br>[fu1y_ace_inh] = '1' | ACE inhibitor TOTAL daily dose (mg)<br>*If unknown use 999.* | text (number, Min: 1, Max: 999), Required |
| 382 | fu1y_arbs<br><br>Show the field ONLY if:<br>[fu1y_done] ='1' and [fu1y_out_mortality] = '0' | ARBs<br>*Angiotensin II receptor blockers* | yesno, Required<br>1 Yes<br>0 No |
| 383 | fu1y_arbs_type<br><br>Show the field ONLY if:<br>[fu1y_arbs] = '1' | ARBs type | radio, Required<br>1 Losartan<br>2 Candesartan<br>3 Irbesartan<br>4 Telmisartan<br>5 Valsartan<br>6 Olmesartan<br>7 Other |
| 384 | fu1y_arbs_other<br><br>Show the field ONLY if:<br>[fu1y_arbs_type] = '7' | If other ARBs specify | text, Required |
| 385 | fu1y_arbs_dose<br><br>Show the field ONLY if:<br>[fu1y_arbs] = '1' | ARBs TOTAL daily dose (mg)<br>*If unknown use 999.* | text (number, Min: 0, Max: 999), Required |
| 386 | fu1y_arni<br><br>Show the field ONLY if:<br>[fu1y_done] ='1' and [fu1y_out_mortality] = '0' | Angiotensin receptor neprilysin inhibitor (ARNI)<br>*Sacubitril/valsartan (Entresto)* | yesno, Required<br>1 Yes<br>0 No |
| 387 | fu1y_arni_dose<br><br>Show the field ONLY if:<br>[fu1y_arni] = '1' | Angiotensin receptor neprilysin inhibitor (ARNI) TOTAL daily dose (mg)<br>*Sacubitril/valsartan (Entresto). If unknown use 999.* | text (number, Min: 0, Max: 999), Required |
| 388 | fu1y_statins<br><br>Show the field ONLY if:<br>[fu1y_done] ='1' and [fu1y_out_mortality] = '0' | Statins | yesno, Required<br>1 Yes<br>0 No |
| 389 | fu1y_statin_type<br><br>Show the field ONLY if:<br>[fu1y_statins] = '1' | Statin type | radio, Required<br>1 Atorvastatin<br>2 Rosuvastatin<br>3 Other |

| | | | |
|---|---|---|---|
| 390 | fu1y_statin_other<br><br>Show the field ONLY if:<br>[fu1y_statin_type] = '3' | If other statin specify | text |
| 391 | fu1y_statin_dose<br><br>Show the field ONLY if:<br>[fu1y_statins] = '1' | Statin TOTAL daily dose (mg)<br>*If unknown use 999.* | text (number, Min: 0, Max: 999) |
| 392 | fu1y_betablocker<br><br>Show the field ONLY if:<br>[fu1y_done] ='1' and [fu1y_out_mortality] = '0' | Beta blocker | yesno, Required<br>1 Yes<br>0 No |
| 393 | fu1y_beta_block_type<br><br>Show the field ONLY if:<br>[fu1y_betablocker] = '1' | Beta Blocker type | radio, Required<br>1 Bisoprolol<br>2 Atenolol<br>3 Metoprolol<br>4 Carvedilol<br>5 Other |
| 394 | fu1y_beta_block_other<br><br>Show the field ONLY if:<br>[fu1y_beta_block_type] = '5' | If other beta blocker specify | text, Required |
| 395 | fu1y_beta_block_dose<br><br>Show the field ONLY if:<br>[fu1y_betablocker] = '1' | Beta Blocker TOTAL daily dose (mg)<br>*If unknown use 999.* | text (number, Min: 0, Max: 999), Required |
| 396 | fu1y_ccb<br><br>Show the field ONLY if:<br>[fu1y_done] ='1' and [fu1y_out_mortality] = '0' | Calcium channel blocker | yesno, Required<br>1 Yes<br>0 No |
| 397 | fu1y_ccb_type<br><br>Show the field ONLY if:<br>[fu1y_ccb] = '1' | Calcium channel blockers type | radio, Required<br>1 Amlodipine<br>2 Diltiazem<br>3 Nicardipine<br>4 Nifedipine<br>5 Verapamil<br>6 Other |
| 398 | fu1y_ccb_other<br><br>Show the field ONLY if:<br>[fu1y_ccb_type] = '6' | If other calcium channel blocker specify | text, Required |
| 399 | fu1y_ccb_dose<br><br>Show the field ONLY if:<br>[fu1y_ccb] = '1' | Calcium channel blocker TOTAL daily dose (mg)<br>*If unknown use 999.* | text (number, Min: 0, Max: 999), Required |
| 400 | fu1y_loop_diuretics<br><br>Show the field ONLY if:<br>[fu1y_done] ='1' and [fu1y_out_mortality] = '0' | Loop diuretic<br>*Example: Furosemide, Torsemide, etc.* | yesno, Required<br>1 Yes<br>0 No |
| 401 | fu1y_loop_diuretic_dose_2<br><br>Show the field ONLY if:<br>[fu1y_loop_diuretics] = '1' | TOTAL daily dose of loop diuretic (mg)<br>*If unknown use 999.* | text (number, Min: 0, Max: 999) |
| 402 | fu1y_mras<br><br>Show the field ONLY if:<br>[fu1y_done] ='1' and [fu1y_out_mortality] = '0' | Mineralocorticoid receptor antagonists (MRAs)<br>*Spironolactone or eplerenone* | yesno, Required<br>1 Yes<br>0 No |
| 403 | fu1y_mras_type<br><br>Show the field ONLY if:<br>[fu1y_mras] = '1' | Mineralocorticoid receptor antagonists type | radio, Required<br>1 Spironolactone<br>2 Eplerenone |

| | | | |
|---|---|---|---|
| 404 | fu1y_mras_dose<br><br>Show the field ONLY if:<br>[fu1y_mras] = '1' | Mineralocorticoid receptor antagonists (MRAs)<br>TOTAL daily dose (mg)<br>*If unknown use 999.* | text (number, Min: 0, Max: 999) |
| 405 | fu1y_oralhypoglc<br><br>Show the field ONLY if:<br>[fu1y_done] ='1' and [fu1y_out_mortality] = '0' | Oral hypoglycemic agents | yesno, Required<br>1 Yes<br>0 No |
| 406 | fu1y_insulin<br><br>Show the field ONLY if:<br>[fu1y_done] ='1' and [fu1y_out_mortality] = '0' | Insulin | yesno, Required<br>1 Yes<br>0 No |
| 407 | fu1y_anticoagulant<br><br>Show the field ONLY if:<br>[fu1y_done] ='1' and [fu1y_out_mortality] = '0' | Anticoagulant treatment | yesno, Required<br>1 Yes<br>0 No |
| 408 | fu1y_anticoagulant_type<br><br>Show the field ONLY if:<br>[fu1y_anticoagulant] = '1' | Anticoagulant treatment<br>**Example: Rivaroxaban, dabigatran, apixaban, or edoxaban.* | radio, Required<br>1 Vitamin K antagonists (acenocumarol)<br>2 Directly acting oral anticoagulants*<br>3 Low-molecular-weight heparin (Enoxaparin, etc) |
| 409 | fu1y_anticoagulant_type2<br><br>Show the field ONLY if:<br>[fu1y_anticoagulant] = '1' | If the patient had received a second anticoagulant treatment, specify which<br>**Example: Rivaroxaban, dabigatran, apixaban, or edoxaban.* | radio<br>1 Vitamin K antagonists (acenocumarol)<br>2 Directly acting oral anticoagulants*<br>3 Low-molecular-weight heparin (Enoxaparin, etc) |
| 410 | fu1y_anticoagulant_why<br><br>Show the field ONLY if:<br>[fu1y_anticoagulant] = '1' | Clinical indication for anticoagulation | radio, Required<br>1 Atrial fibrillation<br>2 Venous thromboembolism<br>3 Pulmonary embolism<br>4 Arterial embolism<br>5 Stroke<br>6 Cardiac intraventricular thrombus<br>7 Prophylaxis |
| 411 | fu1y_anticoagulant_why_2<br><br>Show the field ONLY if:<br>[fu1y_anticoagulant] = '1' | If there is a second clinical indication for anticoagulation, please detail | radio<br>1 Atrial fibrillation<br>2 Venous thromboembolism<br>3 Pulmonary embolism<br>4 Arterial embolism<br>5 Stroke<br>6 Cardiac intraventricular thrombus<br>7 Prophylaxis |
| 412 | fu1y_lwmh_tx_type<br><br>Show the field ONLY if:<br>[fu1y_anticoagulant_type] = '3' or [fu1y_anticoagulant_type2] = '3' | Please select the used Low-molecular-weight heparin | radio, Required<br>1 Enoxaparin<br>2 Dalteparin<br>3 Bemiparin<br>4 Tinzaparin<br>5 Fondaparinux<br>6 Nadroparin<br>7 Certoparin<br>8 Other |

| | | | |
|---|---|---|---|
| 413 | fu1y_anticoag_other<br><br>Show the field ONLY if:<br>[fu1y_lwmh_tx_type] = '8' | If other, please specify the type of Low-molecular-weight heparin: | text, Required |
| 414 | fu1y_lmwh_dose<br><br>Show the field ONLY if:<br>[fu1y_anticoagulant_type] = '3' or [fu1y_anticoagulant_type2] = '3' | Please provide the dose of Low-molecular-weight heparin (only numbers).<br>*Example: 40 mg or 4000 UI (only numbers). If unknown use 999.* | text (number, Min: 0, Max: 10000000), Required |
| 415 | fu1y_lmwh_unit<br><br>Show the field ONLY if:<br>[fu1y_anticoagulant_type] = '3' or [fu1y_anticoagulant_type2] = '3' | Which is the Low-molecular-weight heparin Unit<br>(mg or UI) | radio, Required<br>1 mg<br>2 UI |
| 416 | fu1y_lmwh_frequency<br><br>Show the field ONLY if:<br>[fu1y_anticoagulant_type] = '3' or [fu1y_anticoagulant_type2] = '3' | Which was the frequency of administration of the Low-molecular-weight heparin | radio, Required<br>1 Each 24 hours<br>2 Each 12 hours |
| 417 | fu1y_noac_type<br><br>Show the field ONLY if:<br>[fu1y_anticoagulant_type] = '2' or [fu1y_anticoagulant_type2] = '2' | If Novel oral anticoagulants was used which one: | radio, Required<br>1 Rivaroxaban (Xarelto)<br>2 Dabigatran (Pradaxa)<br>3 Apixaban (Eliquis)<br>4 Edoxaban (Lixiana) |
| 418 | fu1y_noac_dose<br><br>Show the field ONLY if:<br>[fu1y_anticoagulant_type] = '2' or [fu1y_anticoagulant_type2] = '2' | Specify the Directly acting oral anticoagulants TOTAL Daily dose (mg)<br>*If unknown use 999.* | text (number, Min: 0, Max: 999) |
| 419 | fu1y_cortis<br><br>Show the field ONLY if:<br>[fu1y_done] ='1' and [fu1y_out_mortality] = '0' | Corticosteroids therapy | yesno, Required<br>1 Yes<br>0 No |
| 420 | fu1y_cortis_type<br><br>Show the field ONLY if:<br>[fu1y_cortis] ='1' | Specify which type of Corticosteroids | radio, Required<br>1 Methylprednisolone<br>2 Dexamethasone<br>3 Prednisone<br>4 Prednisolone<br>5 Hydrocortisone<br>6 Other |
| 421 | fu1y_cortis_other<br><br>Show the field ONLY if:<br>[fu1y_cortis_type] = '6' | If other corticosteroids, please specify: | text, Required |
| 422 | fu1y_cortis_dose<br><br>Show the field ONLY if:<br>[fu1y_cortis] ='1' | Specify the dose of corticosteroids (mg)?<br>*Example: 20 mg (only numbers). If unknown use 999.* | text (number, Min: 0, Max: 999), Required |
| 423 | fu1y_other_tx<br><br>Show the field ONLY if:<br>[fu1y_done] ='1' and [fu1y_out_mortality] = '0' | If any other relevant therapy, specify which<br>*Use a comma to separate.* | notes |
| 424 | year_outcomes_medications_complete | Section Header: *Form Status*<br>Complete? | dropdown<br>0 Incomplete<br>1 Unverified<br>2 Complete |

Instrument: **Event adjudication** (event_adjudication) Collapse

| | | | |
|---|---|---|---|
| 425 | adjudication_performed<br>Show the field ONLY if: [inhosp_out_mortality] = '1' or [fu1y_out_mortality] = '1' | Section Header: *Event adjudication*<br>Can death be adjudicated? | yesno, Required<br>1 Yes<br>0 No |
| 426 | adjudication_type_of_death<br>Show the field ONLY if: [adjudication_performed] = '1' | It was a cardiovascula death? | yesno, Required<br>1 Yes<br>0 No |
| 427 | adjudication_cv_death_type<br>Show the field ONLY if: [adjudication_type_of_death] = '1' | Suspected cardiovascular death | radio, Identifier<br>1 Death caused by acute myocardial infarction<br>2 Death caused by sudden cardiac, including unwitnessed<br>3 Death resulting from heart failure<br>4 Death caused by stroke<br>5 Death caused by cardiovascular procedures<br>6 Death resulting from cardiovascular hemorrhage<br>7 Death resulting from other cardiovascular cause |
| 428 | adjudication_noncv_death_type<br>Show the field ONLY if: [adjudication_type_of_death] = '0' | Suspected Non-cardiovascular death | radio, Required<br>1 Death resulting from malignancy<br>2 Death resulting from pulmonary causes<br>3 Death caused by infection (includes sepsis)<br>4 Death resulting from gastrointestinal causes<br>5 Death resulting from accident/trauma<br>6 Death caused by other noncardiovascular organ failure<br>7 Death resulting from other noncardiovascular cause<br>8 Undetermined cause of death |
| 429 | adjudication_comments<br>Show the field ONLY if: [inhosp_out_mortality] = '1' or [fu1y_out_mortality] = '1' | Adjudication comments (If apply): | notes |
| 430 | event_adjudication_complete | Section Header: *Form Status*<br>Complete? | dropdown<br>0 Incomplete<br>1 Unverified<br>2 Complete |

CV COVID-19 registry

# Long-term Effects of Coronavirus Disease 2019 on the Cardiovascular System: CV COVID-19 registry

# Statistical Analysis Plan

**CONFI DENTIA L**

Version history table

| Version | Change reason | Updated | Review |
|---|---|---|---|
| 09/02/2022 | Document created | Jose Montes | Dr. Luis García Ortega-Paz |
| | | | |
| | | | |

# SIGNATURE PAGE

**IDIBAPS Staff:**

Dr. Luis García Ortega-Paz | February 9, 2022

Principal investigator | Sign | Date

**EfficeResearch Staff:**

Firmado digitalmente por José Montes
Nombre de reconocimiento (DN): cn=José Montes, o=Effice Research, ou=Head of Biometry, email=jose.montes@effice.es, c=ES
Fecha: 2022.02.09 09:40:44 +01'00'

Jose Montes Garcia ____________________ ____________________

Biostatistician | Sign | Date

# TABLE OF CONTENTS

SIGNATURE PAGE....2

TABLE OF CONTENTS....3

ABBREVIATIONS....5

1 INTRODUCTION....5

2 STUDY OBJECTIVES AND ENDPOINTS....6

2.1 Study objectives....6

2.2 Study endpoints....6

2.2.1 Primary endpoint....6

2.2.2 Secondary endpoints....6

3 STUDY METHODS....7

3.1 General study design....7

3.2 Selection criteria....7

3.2.1 Inclusion criteria....7

3.2.2 Exclusion criteria....7

3.3 Randomisation and blinding....7

3.4 Study variables....7

4 SAMPLE SIZE....8

5 GENERAL CONSIDERATIONS....9

5.1 Timing of analyses....9

5.2 Analysis populations....9

5.3 Subgroups....9

5.4 Missing data....9

5.5 Multi-centre studies....9

5.6 Multiple testing....9

6 SUMMARY OF STUDY DATA....10

6.1 Subject disposition....10

6.2 Demographic and baseline variables ..... 10
6.3 Concurrent illnesses and medical conditions ..... 10
6.4 Prior and concurrent medications ..... 11
6.5 COVID-19 status ..... 11
6.6 Treatment compliance ..... 11
7 PRINCIPAL ANALYSES ..... 12
7.1 General analysis ..... 12
7.2 Primary analysis ..... 12
7.2.1 Sensitivity analysis ..... 13
7.3 Secondary analyses ..... 14
7.4 Other pre-specified analyses ..... 15
7.4.1 Prediction Model of long-term adverse events ..... 15
7.4.2 Vaccination status and CV clinical outcomes ..... 15
7.4.3 In-hospital Major bleeding and cardiovascular outcomes ..... 15
7.4.4 In-hospital arterial and venous thrombotic events and their relationship with death 16
7.4.5 Antithrombotic therapy and clinical outcomes ..... 16
7.4.6 Cardiac injury and its relationship with long-term adverse events. ..... 17
7.4.7 The use of RAAS and clinical outcomes ..... 17
7.4.8 Severe cardiac arrhythmias ..... 18
8 FIGURES ..... 18
9 REPORTING CONVENTIONS ..... 18
10 TECHNICAL DETAILS ..... 19
11 CHANGES TO THE PROTOCOL ..... 19
12 REFERENCES ..... 19

# ABBREVIATIONS

| | |
|---|---|
| AE | Adverse Event |
| CRF | Case Report Form |
| IMP | Investigational Medical Product |
| ITT | Intention to treat |
| PPC | Per protocol |
| SAP | Statistical Analysis Plan |
| SP | Safety population |

# 1 INTRODUCTION

The objective of this statistical analysis plan (SAP) is to specify the statistical analysis in more detail than stated in the protocol for the trial. The statistical analysis plan does not change the analysis described in the protocol, but it should be precise enough to serve as a guideline for statistical programming and creation of tables.

This Statistical Analysis Plan was developed with reference to the valid protocol (Version 1.0 14/05/2020).

# 2 STUDY OBJECTIVES AND ENDPOINTS

## 2.1 Study objectives

The Cardiovascular COVID-19 (CV COVID-19) registry will aim to a better understanding of the long-term cardiovascular implications in patients who had a SARS-CoV-2 infection.

## 2.2 Study endpoints

### *2.2.1 Primary endpoint*

The primary outcome will be cardiovascular mortality at 1 year, defined according to the Academic Research Consortium-2.

### *2.2.2 Secondary endpoints*

The secondary outcomes will be the following endpoints at 1 year:

- All-cause mortality
- Myocardial infarction: defined according to the Academic Research Consortium-2.
- Stroke: defined according to the Academic Research Consortium-2.
- Heart failure hospitalization must be documented in the diagnosis of the hospitalization discharge letter.
- Pulmonary embolism must be documented with a computed tomography.
- Cardiac arrhythmias must be documented in the diagnosis of the hospitalization discharge letter. Serious cardiac arrhythmias were defined as: bradycardia requiring intravenous medication or pacemaker, supraventricular tachycardia requiring intravenous medication or cardioversion, or ventricular tachycardia requiring intravenous medication or cardioversion.
- Major bleeding was defined as a type 3 of the Bleeding Academic Research Consortium (BARC) or higher.

# 3 STUDY METHODS

## 3.1 General study design

It is a multicenter, observational, retrospective registry to be conducted at 17 centers in Spain and Italy. This study is an investigator-initiated registry, and the promoter is the August Pi i Sunyer Biomedical Research Institute (IDIBAPS).

## 3.2 Selection criteria

### *3.2.1 Inclusion criteria*

All inclusion criteria must be met:

1. At least 18 years of age.

2. Patient who underwent a nasopharyngeal swab for real-time reverse transcriptase-polymerase chain reaction (RT-PCR) for SARS-CoV2 between February 2020 and July 2021.

### *3.2.2 Exclusion criteria*

Those with terminal diseases and a life expectancy <1 year before the diagnosis will be excluded.

## 3.3 Randomisation and blinding

Not applicable

## 3.4 Study variables

Study data will be collected and managed using REDCap electronic data capture tools hosted at Hospital Clínic of Barcelona (redcap.clinic.cat).

An anonymized and predefined electronic Case Report Form (eCRF) developed by the investigators will be filled by each participating center. The selected variables are oriented to the cardiovascular risk factors, conditions, medications, and outcomes. Moreover, based on the current scientific literature, specific COVID-19 variable and treatment will be also collected. Charlson Comorbidity Index (CCI) will be used to assess patient’s comorbidities.

Independent study monitors will verify the adequacy of the follow-up and events reported, conducting auditing in a random sample of 10% of all patients included. All events will be adjudicated and classified by an independent event adjudication committee (IEAC) by reviewing source documents (including angiograms) provided by each center.

| Baseline characteristics | Acute phase | Long-term (until 1 year) |
|---|---|---|
| **Demographic data** | **Covid-19 disease** | **Outcomes** |
| Age | Symptoms | All cause death |
| Sex | Hospitalization (Length of stay) | Cardiovascular death |
| **Risk factors/baseline comorbidities** | ICU admission | ACS |
| Hypertension | Invasive mechanical ventilation | Stroke |
| Diabetes mellitus | **Drugs** | Heart failure hospitalization |
| Hypercholesterolemia | Anti-hypertensive drugs | PE |
| Smoking status | Cardiovascular medication | Cardiac arrhythmias |
| Kidney chronic disease | Anticoagulation | |
| Previous ACS | Antivirals | |
| Previous Stroke | Immunosuppressive drugs | |
| Heart failure | Vasopressors | |
| Atrial fibrillation | **Biomarkers** | |
| Previous PE and DVT | Hemoglobin* | |
| Cancer | Lymphocytes* | |
| Organ transplant | Platelets* | |
| Immunosuppression | Creatinine** | |
| Frailty | HscTnI** | |
| Charlson Comorbidity Index | NT-proBNP** | |
| **Baseline medication** | D-dimer** | |
| Anti-hypertensive drugs | Fibrinogen* | |
| Cardiovascular medication | Prothrombin time** | |
| Chronic anticoagulation | **In-hospital events** | |
| Oral hypoglycemic agents | All-cause death | |
| Insulin | Cardiovascular death | |
| Proton pump inhibitors | ACS | |
| NSAIDs | Stroke | |
| | DVT and PE | |
| | Cardiac arrhythmias | |
| | Bleeding | |
| | Red blood cell transfusion | |
| | Discharge medication | |

ACS, acute coronary syndrome; DVT, deep venous thrombosis; Hs-cTnI, high sensitivity cardiac troponin I; NSAIDs, non-steroidal anti-inflammatory drugs; PE, pulmonary embolism.

*Lowest values during hospitalization.**Highest values during hospitalization.

# 4 SAMPLE SIZE

We did not estimate a precise sample size, due to lack of literature reports and we aim to get the maximum numbers of patients possible.

# 5 GENERAL CONSIDERATIONS

## 5.1 Timing of analyses

No interim analyses will be performed.

The final analysis will be performed when all subjects have completed the follow-up visit or dropped out prior to the follow-up visit.

The data will be transferred to the file YYYYMMDD_BBDD_lock_CV_COVID.xlsx, having been documented as meeting the cleaning and approval requirements. Database finalization and locking (including re-opening) will be done after the finalisation and approval of this SAP document.

The first draft analyses will be released the following month to database closure.

## 5.2 Analysis populations

This section is designed to identify the characteristics needed for inclusion in particular populations used in the analyses and give criteria to determine if a subject or observational unit belongs to that population.

The analysis population is all eligible subjects who have at least these data available: date and result of COVID-19 swab. In addition, we will exclude all patients with negative swabs except from the ones from the Clinic Hospital (coordinating centre in charge of including the control cohort). All analysis will be realized from this population.

Patients will be classified into two groups, according to the results of the RT-PCR: COVID-19 positive or negative.

## 5.3 Subgroups

The subgroup of discharged COVID-19 patients will be defined as swab positive patients who were discharged alive. In this subgroup, the following analysis will be performed: long-term adverse events between asymptomatic vs. symptomatic patients or hospitalized vs. not hospitalized (depending on whether the sample size allows the analysis)

## 5.4 Missing data

No imputations data will be performed.

## 5.5 Multi-centre studies

No analyses by site will be performed.

## 5.6 Multiple testing

No applicable

# 6 SUMMARY OF STUDY DATA

All continuous variables will be summarised using the following descriptive statistics: n (based on non-missing sample size), mean, standard deviation, 95% confidence interval, median, maximum and minimum. The frequency and percentages (based on the non-missing sample size) of observed levels will be reported for all categorical measures. In general, all data will be listed, sorted by site and subject, and when appropriate by visit number within subject. All summary tables will be structured with a column for a total of patient and for each group (COVID-19 positive or negative) including any missing observations.

## 6.1 Subject disposition

A summary of enrolment patients (dates, number of patients, exclusions…) will be described. A flowchart of patients and Kaplan Meier curves of follow-up time will be displayed in section of subject disposition.

## 6.2 Demographic and baseline variables

Next variables will be described:

- Demographic data: age (years), gender (male, female)
- Anthropometric data: height (cm), weight (Kg)
- Toxic habits: smoking status

Next variables could be considered how a transformation of data:

- Age coarsening into 18-45 and over 65
- Body mass index defined as the body mass in kg divided by the square of the body height in meters, expressed in units of $kg/m^2$

These cut offs based on by statistical and bibliography references.

## 6.3 Concurrent illnesses and medical conditions

The following medical conditions and concurrent illnesses will be summarized using absolute and relative frequencies: diabetes, Hypertension, Hypercholesterolemia, Chronic Kidney Disease, Atrial fibrillation or Atrial flutter, Family History, Stroke or transient ischemic attack, Myocardial infarction, Percutaneous coronary intervention, Coronary artery bypass grafting, Peripheral Vascular Disease, Chronic obstructive pulmonary disease (COPD) or Asthma, history of pneumonia, Heart Failure, Pulmonary hypertension, Venous Thromboembolism/Pulmonary Embolism, Major bleeding, dementia or cognitive impairment, Cancer, organ transplant.

## 6.4 Prior and concurrent medications

The following prior and concurrent medications will be summarized using absolute and relative frequencies: Aspirin, $P2Y_{12}$ inhibitor, ACE inhibitor, ARBs, Angiotensin receptor neprilysin inhibitor, Statins, Beta Blocker, Calcium channel blocker, Loop diuretic, Mineralocorticoid receptor antagonists, Proton-pump inhibitors, Oral hypoglycemic agents, Insulin, Anticoagulant treatment, Nonsteroidal anti-inflammatory drugs, Paracetamol.

## 6.5 COVID-19 status

The following characteristics of COVID-19 will be described:

- Status of patient (positive, negative)
- Symptoms (fever, dyspnoea, cough, diarrhoea, anosmia or ageusia)
- Hospitalization (time of hospitalization, ICU, time of ICU, renal replacement therapy, vasoactive agents, cardiac catheterization, percutaneous coronary intervention, in-hospital treatments, COVID-19 specific treatments)
- Biomarkers
- Discharge treatments

## 6.6 Treatment compliance

Not applicable.

# 7 PRINCIPAL ANALYSES

## 7.1 General analysis

Differences in proportions will be tested with Chi-square test or Fishers exact test and differences in continuous variables will be tested with a student's t-test (or U Mann-Whitney). Kaplan-Meier method will be used to derive the event rates at follow-up and to plot time-to-event curves. Patients not eligible for 1-year follow-up will be considered at risk until the date of last contact, at which point they will be censored. To determine the predictors of cardiovascular death, a Cox proportional hazards model will be used together with the Wald test.

All p-values will be two-sided and a value <0.05 will be considered statistically significant.

## 7.2 Primary analysis

The primary endpoint is the time (in days) from COVID-19 swab date to the cardiovascular (CV) mortality. Primary endpoint will be compared between the two groups: COVID-19 positive or negative.

An event is defined as a CV death if adjudicated and classified by an IEAC as CV death. If the event cannot be classified and adjudicated by the IEAC, the event will be considered CV death if reported as CV death by the local site investigator. Patients without an event (e.g. due to loss to follow-up) will be censored at the last date known to be alive. Kaplan-Meier estimates for the time to event will be plotted and cumulative incidence estimates at 6 months and at 1 year with 95% confidence intervals will be performed by groups. Median event time will be determined including the corresponding 95%CI.

Univariate analysis of variables will be performed using the Log-rank test. All significant factors that influence primary endpoint will be further analysed in a multivariable analysis using Cox' regression. A backward selection procedure in a Cox' regression will be applied to identify independent prognostic factors for primary endpoint. The hazard ratios for the prognostic factors (including corresponding 95%CI) will be calculated.

### *7.2.1 Sensitivity analysis*

Two sensitivity analysis will be performed to confirm the principal results

Competing risk analysis

Competing risk survival analysis methods will be applied to estimate the cumulative incidence of developing events over time from swab date. These methods allow for the fact that a patient may experience an event which is different from cardiovascular death. These events are known as competing risk events and may preclude the onset of the event of interest or may modify the probability of the onset of that event. In particular, a patient may die from causes other than cardiovascular causes. In a Kaplan-Meier estimation approach, these individuals would be treated as censored and would be eliminated from the risk set, leading to misleading results. Using competing risks, the probability of any event happening is partitioned into the probabilities for each type of event. In our case, only all-deaths will be considered as competing risk events. This sensitivity analysis will be performed for primary endpoint.

Comparison of Investigator-reported and IEAC outcome events

The principal analysis will be repeated with three different definitions of CV death:

- An event is defined as a CV death if the event was adjudicated and classified by an IEAC. If the event could not be classified by the IEAC, patients will be censored at event date.
- An event is defined as a CV death if the event was adjudicated and classified by an IEAC. If the event could not be classified by the IEAC, all unadjudicated deaths will be classified as CV death.
- An event is defined as a CV death if the event is reported by the principal investigator as cardiovascular death.

## 7.3 Secondary analyses

The secondary clinical endpoints are all-cause mortality, acute myocardial infarction (MI), coronary revascularization, stroke, heart failure (HF) hospitalization, thromboembolism, pulmonary embolism (PE), major bleeding, and serious cardiac arrhythmias at 1 year.

In addition, the following combined endpoints will also be analysed: MACE (CV death, MI, stroke), NACE (all-cause death, MI, stroke, and bleeding BARC 3 or 5), MACE+HF (CV death, MI, stroke, and HF hospitalization), MACE + HF + serious cardiac arrhythmia, any arterial thrombotic event (any MI, ischemic stroke, any arterial embolism), any venous thrombotic event (any PE, any deep vein thrombosis) and major bleeding + blood transfusion.

The secondary outcomes are the time (in days) from COVID-19 swab date to each secondary clinical endpoint (or first event occurs in combined endpoints). Secondary outcomes will be compared between the two groups: COVID-19 positive or negative.

For these clinical secondary endpoints and combined endpoints, analysis will be similar to the primary endpoint. Patients without an event (e.g. due to loss to follow-up) will be censored at the last date known to be alive. For each secondary endpoint, Kaplan-Meier estimates for the time to event will be plotted and cumulative incidence estimates at 6 months and at 1 year with 95% confidence intervals will be performed by COVID-19 positive or negative. Median event time will be determined including the corresponding 95%CI.

Univariate analysis of variables will be performed using the Log-rank test. All significant factors that influence secondary endpoints will be further analysed in a multivariable analysis using Cox' regression. A backward selection procedure in a Cox’ regression will be applied to identify independent prognostic factors for secondary endpoints. The hazard ratios for the prognostic factors (including corresponding 95%CI) will be calculated.

These methods will be applied for the entire cohort and various subgroups defined in epigraph 5.3.

Finally, an additional analysis of intrahospital events will be performed. In hospitalization population (patients who were hospitalized), number and percentage of patients with each type of intrahospital events will be calculated and compared between the COVID-19 positive or negative patients using Chi-square test or Fishers exact test.

## 7.4 Other pre-specified analyses

### *7.4.1 Prediction Model of long-term adverse events*

A prediction model will be developed to estimate a cardiovascular event's risk during the follow-up. The data from the registry will be used for the derivation and internal validation of the model. Subsequently, the model will be externally validated in an independent dataset. The complete derivation and validation process will be done according to the Transparent reporting of a multivariable prediction model for individual prognosis or diagnosis (TRIPOD) statement. The main variable for prediction will be a major adverse cardiac event (MACE), a composite endpoint of all-cause death, any MI, ischemic stroke, any venous or arterial thrombotic event, heart failure hospitalization, or any serious arrhythmia. A stepwise approach will perform, univariate predictors of MACE will be determined, significant univariate predictors will be then analyzed is a multivariate logistic regression. The independent predictors of MACE will then use for building the prediction model.

### *7.4.2 Vaccination status and CV clinical outcomes*

The primary endpoint and secondary endpoints will be compared between the two groups: fully vaccinated patient and non-vaccinated patients. These analyses will be performed on patients who are alive at the time of discharge and their vaccination status is known. The endpoints are the time (in days) from date of COVID vaccination or date of discharge to each event.

Patients without an event (e.g. due to loss to follow-up) will be censored at the last date known to be alive. Kaplan-Meier estimates for the time to event will be plotted and cumulative incidence estimates at 6 months and at 1 year with 95% confidence intervals will be performed by groups. Median event time will be determined including the corresponding 95%CI.

Univariate analysis of variables will be performed using the Log-rank test. All significant factors that influence primary endpoint will be further analysed in a multivariable analysis using Cox' regression. A backward selection procedure in a Cox' regression will be applied to identify independent prognostic factors for primary endpoint. The hazard ratios for the prognostic factors (including corresponding 95%CI) will be calculated.

### *7.4.3 In-hospital Major bleeding and cardiovascular outcomes*

The cardiovascular outcomes will be compared between the two groups: patients with in-hospital bleeding and patients without in-hospital bleeding. These analyses will be performed on all patients. The endpoints are the time (in days) from date of discharge to each cardiovascular outcome.

Patients without an event (e.g. due to loss to follow-up) will be censored at the last date known to be alive. Kaplan-Meier estimates for the time to event will be plotted and cumulative incidence estimates at 6 months and at 1 year with 95% confidence intervals will be performed by groups. Median event time will be determined including the corresponding 95%CI.

Univariate analysis of variables will be performed using the Log-rank test. All significant factors that influence primary endpoint will be further analysed in a multivariable analysis using Cox' regression. A backward selection procedure in a Cox' regression will be applied to identify independent prognostic factors for primary endpoint. The hazard ratios for the prognostic factors (including corresponding 95%CI) will be calculated.

### *7.4.4 In-hospital arterial and venous thrombotic events and their relationship with death*

The patient survival will be compared between the two groups: patients with in-hospital arterial or venous thrombotic events and patients without in-hospital arterial or venous thrombotic events. These analyses will be performed on all patients. The endpoints are the time (in days) from date of discharge to death.

Patients without an event (e.g. due to loss to follow-up) will be censored at the last date known to be alive. Kaplan-Meier estimates for the time to event will be plotted and cumulative incidence estimates at 6 months and at 1 year with 95% confidence intervals will be performed by groups. Median event time will be determined including the corresponding 95%CI.

Univariate analysis of variables will be performed using the Log-rank test. All significant factors that influence primary endpoint will be further analysed in a multivariable analysis using Cox' regression. A backward selection procedure in a Cox' regression will be applied to identify independent prognostic factors for primary endpoint. The hazard ratios for the prognostic factors (including corresponding 95%CI) will be calculated.

### *7.4.5 Antithrombotic therapy and clinical outcomes*

The patient survival will be compared between the two groups: patients who were under antithrombotic therapy (anticoagulants and antiplatelet agents) and patients without antithrombotic therapy. These analyses will be performed on all the cohort of patients. The endpoints are the time (in days) from date of diagnosis to death. Patients without an event (e.g. due to loss to follow-up) will be censored at the last date known to be alive. Kaplan-Meier estimates for the time to event will be plotted and cumulative incidence estimates at 6 months and at 1 year with 95% confidence intervals will be performed by groups. Median event time will be determined including the corresponding 95%CI.

Univariate analysis of variables will be performed using the Log-rank test. All significant factors that influence primary endpoint will be further analysed in a multivariable analysis using Cox' regression. A backward selection procedure in a Cox' regression will be applied to identify independent prognostic factors for primary endpoint. The hazard ratios for the prognostic factors (including corresponding 95%CI) will be calculated.

### *7.4.6 Cardiac injury and its relationship with long-term adverse events.*

The patient survival will be compared between the two groups: patients who had cardiac injury and patients without cardiac injury. These analyses will be performed on all the cohort pf patients. The endpoints are the time (in days) from date of diagnosis to death. Patients without an event (e.g. due to loss to follow-up) will be censored at the last date known to be alive. Kaplan-Meier estimates for the time to event will be plotted and cumulative incidence estimates at 6 months and at 1 year with 95% confidence intervals will be performed by groups. Median event time will be determined including the corresponding 95%CI.

Univariate analysis of variables will be performed using the Log-rank test. All significant factors that influence primary endpoint will be further analysed in a multivariable analysis using Cox' regression. A backward selection procedure in a Cox' regression will be applied to identify independent prognostic factors for primary endpoint. The hazard ratios for the prognostic factors (including corresponding 95%CI) will be calculated.

### *7.4.7 The use of RAAS and clinical outcomes*

The patient survival will be compared between the two groups: patients who were under RAAS therapy and patients without RAAS therapy. These analyses will be performed on all the cohort of patients. The endpoints are the time (in days) from date of diagnosis to death. Patients without an event (e.g. due to loss to follow-up) will be censored at the last date known to be alive. Kaplan-Meier estimates for the time to event will be plotted and cumulative incidence estimates at 6 months and at 1 year with 95% confidence intervals will be performed by groups. Median event time will be determined including the corresponding 95%CI.

Univariate analysis of variables will be performed using the Log-rank test. All significant factors that influence primary endpoint will be further analysed in a multivariable analysis using Cox' regression. A backward selection procedure in a Cox' regression will be applied to identify independent prognostic factors for primary endpoint. The hazard ratios for the prognostic factors (including corresponding 95%CI) will be calculated.

### *7.4.8 Severe cardiac arrhythmias*

The patient survival will be compared between the two groups: patients who had a severe cardiac arrhythmia and patients without had a severe cardiac arrhythmia. These analyses will be performed on all the cohort of patients. The endpoints are the time (in days) from date of diagnosis to death. Patients without an event (e.g. due to loss to follow-up) will be censored at the last date known to be alive. Kaplan-Meier estimates for the time to event will be plotted and cumulative incidence estimates at 6 months and at 1 year with 95% confidence intervals will be performed by groups. Median event time will be determined including the corresponding 95%CI.

Univariate analysis of variables will be performed using the Log-rank test. All significant factors that influence primary endpoint will be further analysed in a multivariable analysis using Cox' regression. A backward selection procedure in a Cox’ regression will be applied to identify independent prognostic factors for primary endpoint. The hazard ratios for the prognostic factors (including corresponding 95%CI) will be calculated.

# 8 FIGURES

Figures will be performed according to the analysis described above.

A flow-chart of disposition of subjects will be plotted as described in section 6.1.

Kaplan Meier curves will be displayed in extent of follow-up, survival, and time to events analysis.

# 9 REPORTING CONVENTIONS

P-values will be reported to 3 decimal places (example: “0.003” or “<0.001”). The mean, standard deviation, and any other statistics other than quantiles, will be reported to one decimal place greater than the original data. Quantiles, such as median, or minimum and maximum will use the same number of decimal places as the original data.

The most commonly performed test for assessing the normality assumption is the Shapiro–Wilk test, which produces an S-W statistic together with a *P*-value for testing the null hypothesis that the data are normally distributed. If the p-value is less than 0.05, then the null hypothesis that the data are normally distributed is rejected.

# 10 TECHNICAL DETAILS

The software used to all statistical analyses, listings, tabulations, and figures will be produced using SAS® Version 9.4.

# 11 CHANGES TO THE PROTOCOL

No changes respect to the protocol will be implemented.

# 12 REFERENCES

No new reference has been used.
